# The contribution of coding variants to the heritability of multiple cancer types using UK Biobank whole-exome sequencing data

**DOI:** 10.1016/j.ajhg.2025.02.013

**Published:** 2025-03-11

**Authors:** Naomi Wilcox, Jonathan P. Tyrer, Joe Dennis, Xin Yang, John R.B. Perry, Eugene J. Gardner, Douglas F. Easton

**Affiliations:** 1 Centre for Cancer Genetic Epidemiology, Department of Public Health and Primary Care, University of Cambridge, Cambridge, UK; 2 Metabolic Research Laboratory, Wellcome-MRC Institute of Metabolic Science, University of Cambridge, Cambridge, UK; 3 MRC Epidemiology Unit, Wellcome-MRC Institute of Metabolic Science, University of Cambridge, Cambridge, UK; 4 Centre for Cancer Genetic Epidemiology, Department of Oncology, University of Cambridge, Cambridge, UK

**Keywords:** heritability, genetics, cancer, rare variants, exome, burden, protein-truncating variants

## Abstract

Genome-wide association studies have been highly successful at identifying common variants associated with cancer; however, they do not explain all the inherited risks of cancer. Family-based studies, targeted sequencing, and, more recently, exome-wide association studies have identified rare coding variants in some genes associated with cancer risk, but the overall contribution of these variants to the heritability of cancer is less clear. Here, we describe a method to estimate the genome-wide contribution of rare coding variants to heritability that fits models to the burden effect sizes using an empirical Bayesian approach. We apply this method to the burden of protein-truncating variants in over 15,000 genes for 11 cancers in the UK Biobank using whole-exome sequencing data on over 400,000 individuals. We extend the method to consider the overlap of genes contributing to pairs of cancers. We found ovarian cancer to have the greatest proportion of heritability attributable to protein-truncating variants in genes (46%). The joint cancer models highlight significant clustering of cancer types, including a near-complete overlap in susceptibility genes for breast, ovarian, prostate, and pancreatic cancer. Our results provide insights into the contribution of rare coding variants to the heritability of cancer and identify additional genes with strong evidence of susceptibility to multiple cancer types.

## Teaser

We developed a method to estimate the contribution of rare coding variants to cancer heritability and applied it to 11 cancers in the UK Biobank. Our results provide insights into the contribution of genes to multiple cancers. We identify a near-complete overlap of genes for breast, ovarian, prostate, and pancreatic cancer.

## Introduction

Genome-wide association studies (GWASs) have been highly successful at identifying common variants associated with disease. Increasingly, association studies are being extended to study rare variants using next-generation sequencing methods. For example, for breast cancer (MIM: 114480), GWASs have identified over 300 common susceptibility loci,^1^^,^^2^^,^^3^ while rare variants in *ATM* (MIM: 607585), *BARD1* (MIM: 601593), *BRCA1* (MIM: 113705), *BRCA2* (MIM: 600185), *CHEK2* (MIM: 604373), *RAD51C* (MIM: 602774), *RAD51D* (MIM: 602954), *PALB2* (MIM: 610355), and *TP53* (MIM: 191170) have been identified through linkage or targeted sequencing studies.^4^ Exome-wide analysis has recently additionally identified rare variants in *MAP3K1* (MIM: 600982) to be associated with breast cancer risk.^5^ Similarly, for bowel cancer (MIM: 114500), GWASs have identified over 200 common susceptibility loci,^6^ and rare variants have been identified in mismatch repair (MMR) genes including *MSH2* (MIM: 609309), *MSH6* (MIM: 600678), *MLH1* (MIM: 120436), and *PMS2* (MIM: 600259), as well other genes including *APC*^7^ (MIM: 611731). The increasing availability of whole-exome sequencing (WES) and whole-genome sequencing data is enabling exome- and genome-wide analysis of rare variants and the discovery of novel rare variants associated with cancer risk.

For common variants, GWAS data can be used to estimate the overall contribution to heritability, using methods including linkage disequilibrium score regression (LDSC),^8^ which uses GWAS summary results, and The Cancer Genome Atlas (TGCA).^9^ LDSC has been used to estimate that common variants explain ∼41% of the familial relative risk of breast cancer and ∼73% of colorectal cancer.^2^^,^^6^ This method has also been extended to estimate the genetic correlation between traits,^10^ which has shown significant correlations in cancer susceptibility, for example, for breast and ovarian cancer (MIM: 167000) and breast and lung cancer^11^^,^^12^ (MIM: 211980). An analogous question is what is the overall contribution of rare coding variants to cancer heritability. While some genes, such as *BRCA1* and *BRCA2*, have long been known to make a significant contribution to the familial aggregation of certain cancers,^5^^,^^13^ the more general question has not been definitively answered since most genes have not been extensively evaluated in association studies.

We previously described a method for evaluating the contribution of the gene-wise burden of rare coding variants to cancer heritability that fits models to the burden effect sizes using an empirical Bayesian approach.^5^ This approach can be implemented using gene burden summary statistics and is not computationally intensive. We previously applied this method to the burden of protein-truncating variants (PTVs) in genes and breast cancer risk using data from the UK Biobank and Breast Cancer Association Consortium (BCAC).^5^ Here, we apply this method to 11 different cancer types using data from the UK Biobank. We extend this method to consider the overlap of genes contributing to pairs of cancers and evaluate the correlation of coding variant heritability among cancers. This method can be used to derive the posterior probability that each gene is cancer associated.

## Material and methods

### Material: UK Biobank

The UK Biobank is a population-based prospective cohort study of more than 500,000 individuals. UK Biobank has approval from the North West Multi-centre Research Ethics Committee (MREC) as a Research Tissue Bank (RTB). More detailed information on the UK Biobank is given elsewhere.^14^^,^^15^ WES data for 450,000 samples were released in October 2021 and accessed via the UK Biobank DNANexus platform.^16^ Quality control (QC) metrics were applied to variant call format (VCF) files as described by Gardner et al., including genotype-level filters for depth and genotype quality.^17^ Other filters, including the exclusion of samples with disagreement between genetically determined and self-reported sex or excess relatives, were applied as described elsewhere.^5^ Analyses were restricted to individuals of European ancestry, as defined by ancestry informative principal components. The final dataset for analysis included 419,307 samples, with 227,393 females and 191,914 males.

Subjects with cancer were determined by linkage to national cancer registration data (NCRAS) and by selecting the appropriate ICD-10 codes (Table 1). For breast cancer, we also included self-reported cancer (7.8% of all subjects with breast cancer) for consistency with previous analysis and since self-reporting a breast cancer diagnosis is known to be accurate.^18^ Subjects with prevalent and incident cancer were included. Only cancers that were an individual’s first or second diagnosed cancer were included. The numbers of subjects with cancer for males and females for each cancer are provided in Table 1. The 11 cancers we considered were the most common solid tumors by incident cancer diagnoses in the UK Biobank, as per the UK Biobank Malignant Cancer Summary Report.^19^

**Table 1:** Cancer ICD10 codes, the number of subjects with and without cancer, and the model used for analysis

	**ICD10 code(s)**	**Female**		**Male**		**FH variable**	**Model**
		Subjects without cancer	Subjects with cancer	Subjects without cancer	Subjects with cancer		
Breast	C50, D05	209,435	17,958	191,820	94	yes	3
Lung	C34	225,612	1,781	189,959	1,955	yes	3
Prostate	C61	227,393	0	180,214	11,700	yes	3
Bowel	C18, C19, C20, C21	224,404	2,989	187,956	3,958	yes	3
Pancreatic	C25	226,900	493	191,326	588	no	2
Endometrial	C54	225,419	1,974	191,914	0	no	1
Ovarian	C56	226,021	1,372	191,914	0	no	1
Esophagus	C15	227,118	275	191,167	747	no	2
Kidney	C64	226,774	619	190,862	1,052	no	2
Bladder	C67	227,072	321	190,780	1,134	no	2
Malignant melanoma	C43	225,111	2,282	189,986	1,928	no	2

Cancers not previously mentioned: endometrial (MIM: 608089), esophagus (MIM: 133239), kidney (MIM: 144700), bladder (MIM: 109800), and malignant melanoma (MIM: 155600).

The Ensembl Variant Effect Predictor (VEP) was used to annotate variants, including the 1000 Genomes phase 3 allele frequency, sequence ontology variant consequences, and exon/intron numbers.^20^ Annotation files were used to identify PTVs (including predicted frameshift, stop gain, start loss, and canonical splice variants). PTVs in the last exon of each gene and the last 50 bp of the penultimate exon were excluded, as these are generally predicted to escape nonsense-mediated mRNA decay (NMD).

### Methods

#### Gene burden tests

To test for associations between rare variants in genes and cancers of interest, we performed simple burden tests where variants within genes are collapsed together. This is a powerful method if variants have similar effect sizes.^21^ We consider the simplest type of burden test where genotypes are collapsed to a 0/1 variable based on whether the samples carry a PTV, including canonical splice-site variants, in a specific gene. That is, $G_{i}=1\;\text{if}\;\sum_{j=1}^{p}g_{ij}>0\;\text{and}\;0\;\text{if}\sum_{j=1}^{p}g_{ij}=0$, where $g_{ij}$ = 0, 1, 2 is the number of minor alleles observed for sample *i* at variant *j* and *p* is the number of PTVs in the gene.

To apply the approach above, we fit logistic regression models in which the carrier status is the outcome variable and the disease phenotype a covariate. This method is described further elsewhere.^22^

When there is no family history information available and the cancer is prevalent in only one sex, e.g., ovarian cancer, the model used is

$$\;\text{model}\;1:\log\left(\frac{P\left(G=1\right)}{1-P\left(G=1\right)}\right)=\alpha +\beta_{1}Case+\beta_{4}x_{4}\ldots$$

When there is no family history information available but the cancer is prevalent in both sexes, e.g., pancreatic cancer (MIM: 260350), the model used is

$$\;\text{model}\;2:\log\left(\frac{P\left(G=1\right)}{1-P\left(G=1\right)}\right)=\alpha +\beta_{1}Case+\beta_{3}Sex+\beta_{4}x_{4}\ldots$$

When family history information is available, e.g., for breast cancer, the model used is

$$\;\text{model}\;3:\log\left(\frac{P\left(G=1\right)}{1-P\left(G=1\right)}\right)=\alpha +\beta_{1}{\left(Case+0.5FH\right)+\beta }_{3}Sex+\beta_{4}x_{4}\ldots$$

We tested for association using the Wald *p* value associated with $\beta_{1}$. Highly significant associations based on small counts may be unreliable, so we also conducted likelihood ratio tests for genes reaching exome-wide significance with ≤ 5 affected carriers. For gene-cancer associations with *p* < 0.001, we additionally performed Firth logistic regression to account for potential imprecision in the *p* values due to small carrier counts.^23^

For breast cancer, the PTV burden results were reported by Wilcox et al.^5^ For the other cancers with available family history information, i.e., bowel, lung, and prostate cancer (MIM: 176807), the results for the PTV burden association analysis are shown elsewhere.^22^ Here, we additionally present the PTV burden association results for the 7 other cancers without family history information.

### Modeling effect sizes

A method to model the effect sizes associated with PTVs, and hence estimate the contribution of PTVs to the familial relative risk of breast cancer, was described by Wilcox et al.^5^ Here, we generalize this method for any cancer in the UK Biobank, as well as extend the method to account for the joint distribution of multiple cancers. For breast cancer results, here we use the UK Biobank data only, whereas the paper by Wilcox et al.^5^ included the BCAC data in the familial relative risk (FRR) calculations.

### Individual cancer model

For individual cancers, we assume a prior distribution for effect sizes (log-odds ratios [OR]) f(β|α,η) in which a proportion, α, of genes are associated with the cancer. For genes that are risk associated, the prior distribution for the log-OR is assumed to follow a negative exponential distribution. Thus,

$$\beta ∼\left\{ \begin{array}{c} 0\;w.p\;1-\alpha \\ g\left(\beta ,\eta \right)\;w.p\;\alpha \; \end{array}\right.,\text{where}\;g\left(\beta ,\eta \right)∼\eta e^{-\eta_{\beta }}\text{.}$$

The effect size is assumed to be the same for all PTVs in a gene. Thus, the distribution is determined by the parameters α and η. These parameters can be fit, using maximum likelihood, from summary counts of the numbers of PTV carriers with and without cancer in each gene and each sex. For cancers where family history data is also available, the method can be extended to incorporate counts by family history. Details of the likelihood derivation are given in the supplemental methods and Wilcox et al.^5^ The estimates of α and η can be used to derive the posterior probability that each gene is risk associated and the median predicted effect size. It can also be used to estimate the familial relative risk to first-degree relatives (λ) attributable to PTVs in all genes under the assumption that the combined effect of PTVs in different genes is additive.^5^^,^^24^ We also express this as an estimated proportion of the overall familial relative risk under the assumption that the overall FRR for each cancer is 2 (which is approximately true for all the cancers considered here^25^^,^^26^) and that the PTVs combine multiplicatively with other common genetic or familial factors.

To model the joint effect of two cancers, we extend the model to allow four categories of gene: genes associated with cancer 1 only, cancer 2 only, both cancers, or neither cancer, with proportions $\alpha_{10}$, $\alpha_{01}$, $\alpha_{11}$, and $1-\alpha_{10}-\alpha_{01}{-\alpha }_{11}$ respectively. The effect sizes for the two cancers, when associated, are again assumed to follow negatively exponential distributions. Thus,

$$\left(\beta_{1},\beta_{2}\right)∼\left\{ \begin{array}{ccc} \begin{array}{c} \begin{array}{c} \left(0,0\right)\\ \left(g\left(\beta_{1}|\eta_{1}\right),0\right) \end{array}\\ \begin{array}{c} \left(0,g\left(\beta_{2}|\eta_{2}\right)\right)\\ \left(g\left(\beta_{1}\left|\eta_{1}),g(\beta_{2}\right|\eta_{2}\right)\right) \end{array} \end{array} & w.p. & \begin{array}{c} \begin{array}{c} \left(1-\alpha_{10}-\alpha_{01}{-\alpha }_{11}\right)\\ \alpha_{10} \end{array}\\ \begin{array}{c} \alpha_{01}\\ \alpha_{11} \end{array} \end{array} \end{array}\right.\text{,}$$

where $g\left(\beta_{1}|\eta_{1}\right)∼\eta_{1}\;\exp\left(-\eta_{1}\beta_{1}\right),g\left(\beta_{2}|\eta_{2}\right)∼\eta_{2}\;\exp\left(-\eta_{2}\beta_{2}\right).$

There are thus five parameters to estimate: $\alpha_{10}$, $\alpha_{01}$, $\alpha_{11}$, $\eta_{1}$, and $\eta_{2}$.

For simplicity, we assume that the effect sizes (when both cancers are associated) $\beta_{1}$ and $\beta_{2}$ are uncorrelated. This is motivated by the fact that the data would be too limited to estimate this correlation in addition to the other parameters. Moreover, for the strongest known genes associated with multiple cancers (e.g., *ATM*, *BRCA1*, and *BRCA2*), there is no clear correlation between the effect sizes for different cancers. To evaluate the evidence for overlap in the susceptibility genes for pairs of cancers, we performed likelihood ratio tests against the null hypothesis that the probabilities that genes are associated with each of the two cancers are independent, i.e., the OR:

$$\psi =\frac{\alpha_{11}\left(1-\alpha_{10}-\alpha_{01}{-\alpha }_{11}\right)}{\alpha_{10}\alpha_{01}}=1\text{.}$$

More details are provided in the supplemental methods.

## Results

### PTV burden results

The PTV burden results for 11 all cancers are summarized in Table 2. Association results for each cancer that were not reported previously,^5^^,^^27^ for genes reaching *p* < 0.001, can be found in Tables S1–S7. The corresponding Manhattan and quantile-quantile (QQ) plots can be found in Figures S1–S14. A comparison of Wald and likelihood ratio test (LRT) *p* values for genes reaching exome-wide significance from the Wald test and with the number of carriers with cancer ≤ 5 for each cancer are shown in Table S8.

**Table 2:** Summary of results for PTV burden tests for 11 cancers

**Cancer**	**Wald test**					**Firth regression**
	**Number of genes at a *p* value threshold**			**Genes with *p* < 2.5 × 10**^**−**^**^6^**	**COSMIC TSGs with *p* < 1 × 10**^**−**^**^4^**	**Genes with *p* < 2.5 × 10**^**−**^**^6^**
	***p* < 0.001**	***p* < 1 × 10**^**−**^**^4^**	***p* < 2.5 × 10**^**−**^**^6^**			
Breast	30	9	6	*BRCA2*, *BRCA1*, *CHEK2*, *PALB2*, *ATM*, *MAP3K1*	*BRCA2*, *BRCA1*, *CHEK2*, *PALB2*, *ATM*, *MAP3K1*, *LZTR1*, *BARD1*	*BRCA2*, *BRCA1*, *PALB2*, *CHEK2*, *ATM*, *MAP3K1*
Prostate	35	8	3	*BRCA2*, *CHEK2*, *ATM*	*BRCA2*, *CHEK2*, *ATM*	*BRCA2*, *CHEK2*, *ATM*
Bowel	42	9	5	*MSH6*, *MSH2*, *MLH1*, *APC*, *GAPDH*	*MSH6*, *MSH2*, *MLH1*, *APC*, *FLCN*, *SMAD4*	*MSH6*, *MSH2*, *MLH1*, *APC*
Lung	46	8	0	N/A	*ARHGAP35*, *BIRC3*	N/A
Pancreatic	100	38	8	*ATM*, *MEN1*, *RCN2*, *YPEL3*, *SMC2*, *SEC14L3*, *GNG10*, *ZNF461*	*ATM*, *MEN1*	*ATM*
Endometrial	80	26	5	*MSH6*, *MLH1*, *ACRV1*, *STK32C*, *PSMC6*	*MSH6*, *MLH1*, *MSH2*	*MSH6*
Ovarian	115	35	9	*BRCA2*, *BRCA1*, *IVD*, *JAML*, *KCNAB2*, *ZFP14*, *TMEM163*, *TMEM167A*, *NHEJ1*	*BRCA2*, *BRCA1*, *NFRSF14*	*BRCA2*, *BRCA1*
Esophagus	117	32	2	*KNL1*, *IRF2BP2*	*KNL1*, *ATM*, *FUS*	N/A
Kidney	89	27	6	*PKD1*, *FGL2*, *TTC9*, *EX0C7*, *NCK2*, *TMEM174*	N/A	N/A
Bladder	72	27	8	*DLX2*, *ZNF506*, *CDCP2*, *TMEM222*, *KDM1A*, *ARHGEF6*, *HR*, *NLRP10*	N/A	N/A
Malignant melanoma	45	12	4	*DCX*, *MED9*, *MRPL44*, *CDKN2A*	*CDKN2A*	*MED9*

Genes not previously mentioned: *LZTR1* (MIM: 600574), *GAPDH* (MIM: 138400), *FLCN* [MIM: 607273), *SMAD4* (MIM: 600993), *ARHGAP35* (MIM: 605277), *BIRC3* (MIM: 601721), *MEN1* (MIM: 613733), *RCN2* (MIM: 602584), *YPEL3* (MIM: 609724), *SMC2* (MIM: 605576), *SEC14L3* (MIM: 612824), *GNG10* (MIM: 604389), *ZNF461* (MIM: 608640), *ACRV1* (MIM: 102525), *STK32C* (MIM: N/A), *PSMC6* (MIM: 602708), *IVD* (MIM: 607036), *JAML* (MIM: 609770), *KCNAB2* (MIM: 601142), *ZFP15* (MIM: 620163), *TMEM163* (MIM: 618978), *TMEM167A* (MIM: 620000), *NHEJ1* (MIM: 611290), *NFRSF14* (MIM: N/A), *KNL1* (MIM: 609173), *IRF2BP2* (MIM: 615332), *FUS* (MIM: 137070), *PKD1* (MIM: 601313), *FGL2* (MIM: 605351), *TTC9* (MIM: 610488), *EX0C7* (MIM: 608163), *NCK2* (MIM: 604930), *TMEM174* (MIM: 614909), *DLX2* (MIM: 126255), *ZNF506* (MIM: N/A), *CDCP2* (MIM: 612320), *TMEM222* (MIM: 619469), *KDM1A* (MIM: 609132), *ARHGEF6* (MIM: 300267), *HR* (MIM: 602302), *NLRP10* (MIM: 609662), *DCX* (MIM: 300121), *MRPL44* (MIM: 611849), and *CDKN2A* (MIM: 600160). The table includes the number of genes reaching different significance thresholds as well as the list of genes that reach exome-wide significance and COSMIC TSGs with Wald test *p* < 1 × 10^−4^ listed in ascending *p* value order. For breast cancer, the results are from the meta-analysis of the UK Biobank and BCAC datasets as reported by Wilcox et al.,^5^ apart from for Firth regression, which was just run in the UK Biobank. The final column shows the genes that remained exome wide significant using Firth regression. N/A, not applicable.

The cancer with the most exome-wide associations by the Wald test was ovarian cancer (9 genes), followed by pancreatic (8 genes) and bladder (8 genes) cancer. We specifically examined tumor-suppressor genes (TSGs) defined by COSMIC since previous analyses of breast cancer indicated that this category was highly enriched.^5^ The cancer with the most COSMIC TSGs having *p* < 1 × 10^−4^ was breast cancer (8 genes), followed by bowel cancer (6 genes). Among TSGs, *ATM* was associated at *p* < 1 × 10^−4^ for breast, prostate, pancreatic, and esophagus cancer; *BRCA2* was associated at *p* < 1 × 10^−4^ for breast, prostate, and ovarian cancer; and *MSH6*, *MSH2*, and *MLH1* were associated at *p* < 1 × 10^−4^ for bowel and endometrial cancer. No COSMIC TSGs had *p* < 1 × 10^−4^ for kidney or bladder cancer. Cancers with >50 genes associated at *p* < 0.001 include pancreatic, endometrial, ovarian, esophagus, kidney, and bladder cancer.

We note that for that for many of the associated genes, the number of carriers with cancer was low. In this situation, the *p* values can be exaggerated and, as expected, were somewhat less significant using Firth regression or likelihood ratio tests. Of the gene-disease associations not reported previously, associations reaching exome-wide associations using all methods included *MSH6* for endometrial cancer, *ATM* for prostate cancer, and *MED9* (MIM: 609878) for melanoma.

Of the genes not listed in Table 2, of interest for ovarian cancer are associations at *p* < 0.05 for 5 putative ovarian cancer genes: *MSH6* (*p* = 0.00056), *BRIP1* (MIM: 605882) (*p* = 0.00055), *RAD51C* (*p* = 0.028), *RAD51D* (*p* = 0.00011), and *CHEK2* (*p* = 0.00049).

### Empirical Bayes modeling

**Figure 1:**
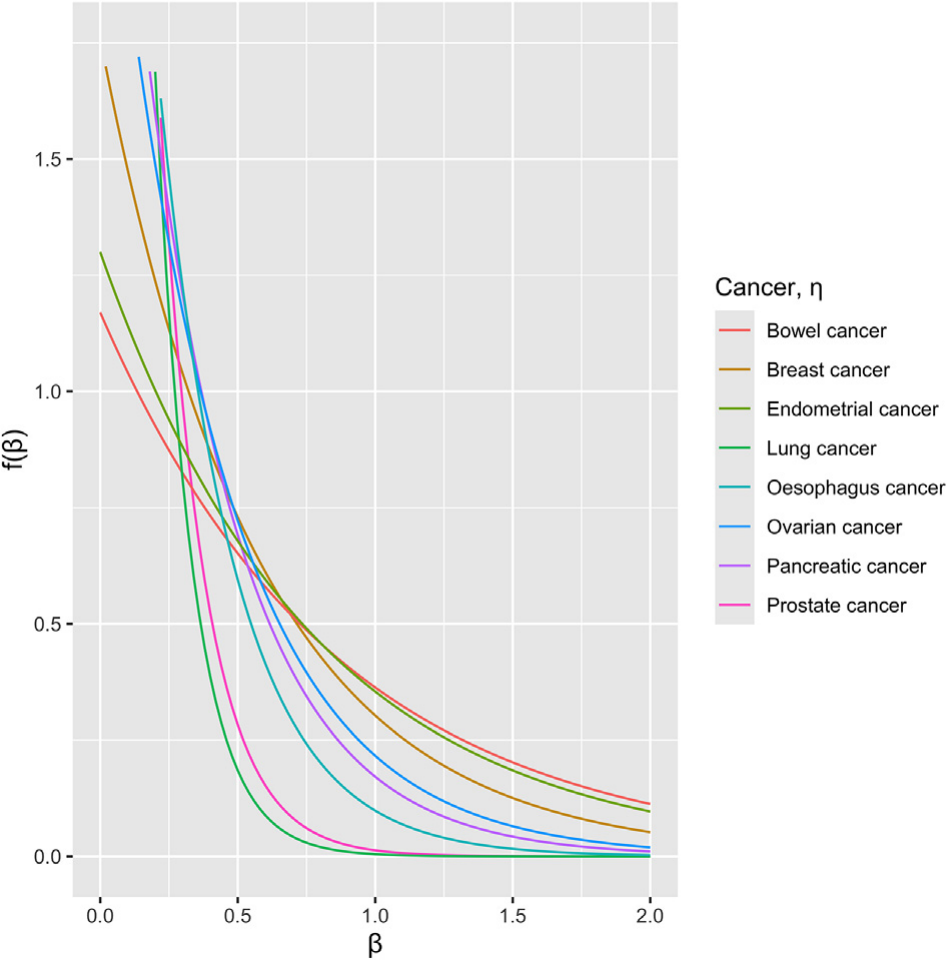
The distributions for the log-odds ratio of associated genes for each cancer This uses the optimized value of η, where $g\left(\beta ,\eta \right)∼\eta e^{-\eta_{\beta }}$.

Table 3 and Figure 1 summarize the best-fitting models for each cancer. The cancer with the greatest estimated proportion of risk-associated genes was ovarian cancer (α = 0.037, equivalent to 578 genes), followed by esophageal cancer (α = 0.030, ≈468 genes) and pancreatic cancer (α = 0.019, ≈297 genes). In contrast, for kidney, bladder, and malignant melanoma, α was estimated to be 0. The estimated exponential distributions for the log-ORs are shown in Figure 1. The graph is least steep for bowel cancer, which has the greatest estimated median OR. In contrast, the median OR was lowest for prostate cancer, consistent with a lower proportion of higher-risk genes.

**Table 3:** Optimized values of α and η for each cancer, as well as the estimated heritability and contribution to familial relative risk

	α	η	**Median OR**	**loglik**	λ	**%FRR**	**Genes with posterior probability >0.9**	**Genes with posterior probability >0.5**
Breast	0.0027	1.8	1.5	713.43	1.06	8.8	*BRCA1*, *BRCA2*, *CHEK2*, *PALB2*, *ATM*, *MAP3K1*	*BRCA1*, *BRCA2*, *CHEK2*, *PALB2*, *ATM*, *MAP3K1*
Prostate	0.020	6.2	1.1	37.73	1.01	1.08	*BRCA2*, *CHEK2*, *ATM*	*BRCA2*, *CHEK2*, *ATM*
Bowel	0.0016	1.2	1.8	140.67	1.05	6.7	*MSH2*, *MSH6*, *MLH1*, *APC*	*MSH2*, *MSH6*, *MLH1*, *APC*, *GAPDH*
Lung	0.0035	7.4	1.1	0.04	1.00	0.1	N/A	N/A
Pancreas	0.019	2.8	1.3	7.26	1.08	10.6	*ATM*	*ATM*
Endometrial	0.0014	1.3	1.7	88.05	1.12	16.5	*MSH6*	*MSH6*
Ovarian	0.037	2.4	1.3	89.26	1.37	45.9	*BRCA2*, *BRCA1*	*BRCA2*, *BRCA1*, *IVD*, *SLC35E4*, *REX05*
Esophagus	0.030	3.6	1.2	0.92	1.04	6.2	N/A	*NLRP12*, *ATM*
Kidney	0.00	4.4	1.2	0.00	1.00	0.0	N/A	N/A
Bladder	0.00	2.5	1.3	0.00	1.00	0.0	N/A	N/A
Malignant melanoma	0.00	5.6	1.1	0.00	1.00	0.0	N/A	N/A

Genes not previously mentioned: *SLC35E4* (MIM: N/A), *REX05* (MIM: N/A), and *NLRP12* (MIM: 609648). The %FRR estimates use posterior gene PTV frequency adjusted for copy-number variation (CNV) frequency. The genes with posterior probability >0.5 are listed in descending order. N/A, not applicable.

In terms of overall contribution to the FRR, the highest proportion was for ovarian cancer (45.9%), followed by endometrial cancer (16.5%; Table 3).

Based on the best-fitting models, we computed the posterior probability of each gene being associated with each cancer (Tables S9–S16). For breast cancer, there were 6 genes with a posterior probability > 0.9: *BRCA2*, *BRCA1*, *PALB2*, *CHEK2*, *ATM*, and *MAP3K1* (Table S9). *BRCA2* and *BRCA1* also reached this level for ovarian cancer, *BRCA2*, *ATM*, and *CHEK2* for prostate cancer, and *ATM* for pancreatic cancer. For bowel cancer, there were 4 genes with a posterior probability > 0.9: the MMR genes *MLH1*, *MSH6*, and *MSH2*, as well as *APC* (Table S10). *MSH6* also reached this level for endometrial cancer. For lung, esophagus, kidney, and bladder cancer, as well as malignant melanoma, there were no genes with a posterior probability > 0.9. Table 4 shows all genes reaching posterior probability >0.8 for at least one cancer.

**Table 4:** Genes with posterior probability >0.8 for at least 1 cancer

	**Cancers**
*APC*	bowel
*ATM*	breast, pancreas, prostate
*BRCA1*	breast, ovarian
*BRCA2*	breast, ovarian, prostate
*CHEK2*	breast, prostate
*MAP3K1*	breast
*MLH1*	bowel
*MSH2*	bowel
*MSH6*	bowel, endometrial
*PALB2*	breast

The cancer columns are the cancers that had a posterior probability > 0.8 of the gene being associated with the cancer.

### Joint cancer models

**Figure 2:**
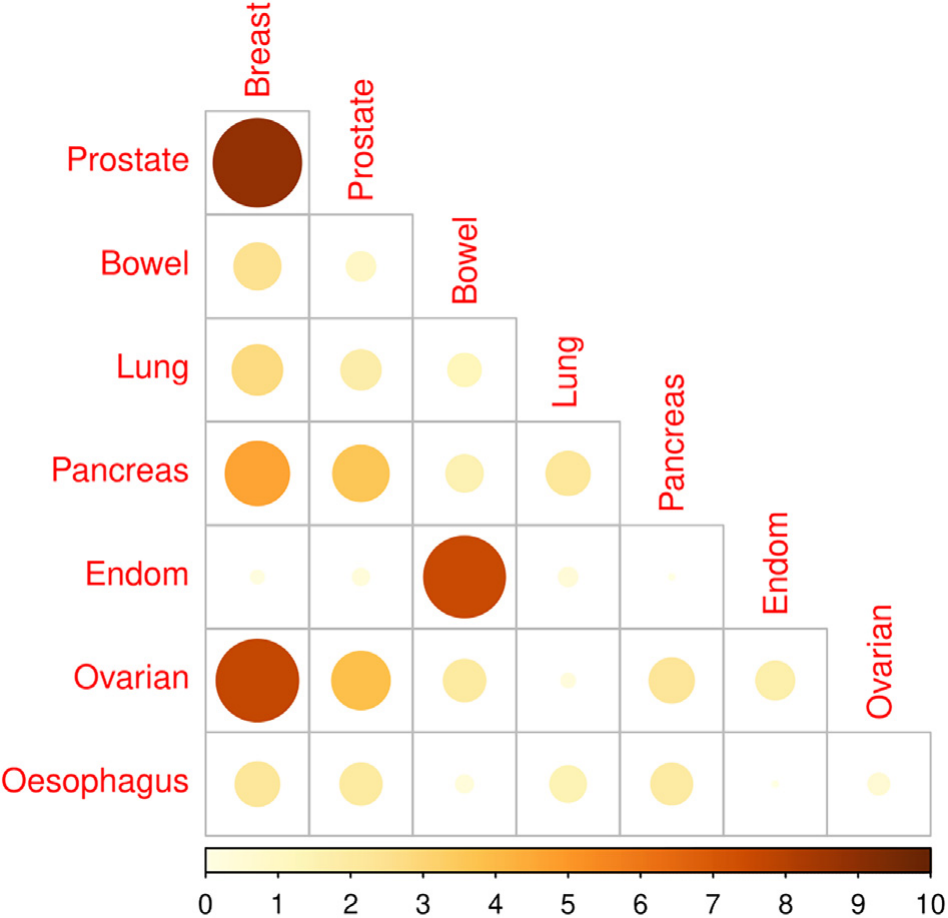
-log10 *p* values from the LRT comparing joint to independent cancer models -log10 *p* values from a likelihood ratio test comparing models in which the prior probabilities that any gene is associated with each of a pair of cancers is related, with corresponding models in which these probabilities are independent (see methods).

**Figure 3:**
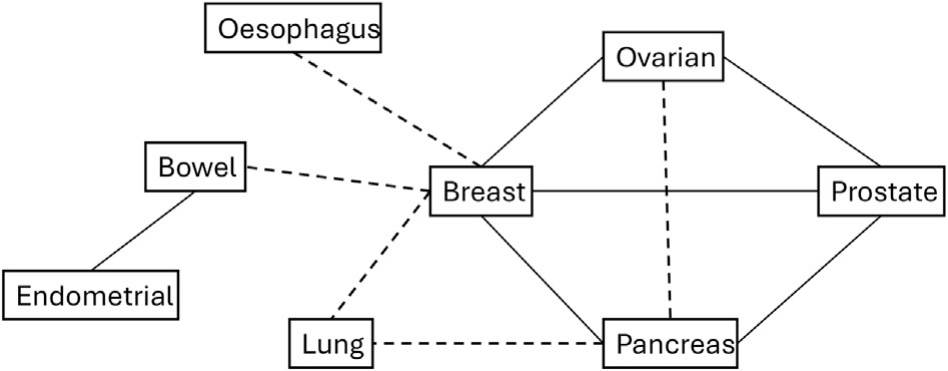
Diagram showing cancer pairs with LRT *p*≤ 0.001 as bold lines and LRT 0.01 ≤*p* < 0.001 as dashed lines

We next considered models incorporating pairs of cancers (Figures 2 and 3). The strongest evidence for overlap was found for breast-prostate (likelihood ratio *p* = 1.5 × 10^−9^), breast-ovarian (*p* = 2.1 × 10^−8^), bowel-endometrial (*p* = 3.0 × 10^−8^), and breast-pancreatic (*p* = 2.3 × 10^−5^). Associations with *p* < 0.001 were additionally observed for prostate-ovarian and prostate-pancreas, with weaker evidence of overlap for breast-bowel, breast-lung, breast-esophagus, and lung-pancreas. More detailed results for pairs with *p* < 0.01 are shown in Table 5 and for all cancer pairs in Table S17.

**Table 5:** Estimated parameters for the best fitting joint cancer models and the LRT p value comparing the joint to independent cancer models

**Cancer 1**	**Cancer 2**	**Method**	$\alpha_{10}$	$\alpha_{01}$	$\alpha_{11}$	${\;\eta }_{1}$	$\eta_{2}$	**LRT**
Breast	Prostate	1	0.00000	0.00000	0.00220	1.49	2.51	1.5 × 10^−9^
Breast	Ovarian	1	0.00000	0.00000	0.00270	1.77	1.23	2.1 × 10^−8^
Bowel	Endometrial	2	0.00020	0.00000	0.00140	1.13	0.93	3.0 × 10^−8^
Breast	Pancreas	1	0.00000	0.00000	0.00230	1.62	1.57	2.3 × 10^−5^
Prostate	Ovarian	1	0.00000	0.00340	0.01700	6.04	2.06	0.00015
Prostate	Pancreas	1	0.00000	0.00000	0.00840	4.56	2.17	0.00028
Breast	Lung	1	0.00013	0.00026	0.00150	1.52	6.00	0.0014
Breast	Bowel	1	0.00053	0.00027	0.00160	1.51	1.00	0.0035
Pancreas	Ovarian	1	0.00000	0.00000	0.03000	3.09	2.25	0.0054
Breast	Esophagus	1	0.00000	0.00980	0.00240	1.64	2.95	0.0062
Lung	Pancreas	1	0.00000	0.00000	0.00460	5.93	2.05	0.0069

Method refers to the joint cancer model method used as described in the material and methods section. Results here have LRT *p* value values < 0.01 and are sorted by ascending LRT *p* value. $\alpha_{10}=P\left(C1\;n\;C2^{\prime }\right),{\;\alpha }_{01}=P\left(C1^{\prime }n\;C2\right),\text{and}\;\alpha_{11}=P\left(C1\;n\;C2\right)$. η_1_ and η_2_, effect size parameters for cancer 1 and cancer 2, respectively.

We note that of the cancer pairs with *p* < 0.001, the breast-prostate, breast-ovarian, breast-pancreatic, prostate-pancreatic, and pancreatic-ovarian pairs all estimated $\alpha_{10}=\alpha_{01}=0\text{,}$ i.e*.*, a complete overlap of associated genes. Genes with a posterior probability > 0.8 of being associated with both cancers for at least 1 cancer pair were *APC*, *ATM*, *BAP1* (MIM: 603089), *BRCA1*, *BRCA2*, *CHEK2*, *MAP3K1*, *MLH1*, *MSH2*, *MSH6*, and *PALB2* (Table S18). *ATM* was associated with the most cancer pairs with a posterior probability > 0.8 (9 pairs). Six of these genes are established genes for one or more of the cancers, the exception being *BAP1*, which had a posterior probability of 0.824 in the breast-prostate model. In the breast-ovarian model, the gene with the next highest posterior probability (after those identified above) was *NHEJ1* (0.300), followed by the established risk genes *RAD51D* (0.282) and *BRIP1* (0.259) (Table S19). *NHEJ1* PTVs were associated with an OR of 2.70 (1.30, 5.61), *p* = 0.0079, for breast cancer and 17.67 (5.36, 58.24), *p* = 2.37 × 10^−6^, for ovarian cancer.

For bowel and endometrial cancer, the best joint model estimated $\alpha_{1}$ = 0.00020 (≈3 genes), $\alpha_{2}$ = 0, and $\alpha_{3}=$ 0.00140 (≈22 genes), i.e., consistent with subsets of genes associated with both cancers and with bowel cancer only but none associated with endometrial cancer alone. There were 3 genes with a posterior probability > 0.9 of being associated with both cancers: *MSH6* (1.00), *MLH1* (0.98), and *MSH2* (0.98) (Table S21). COSMIC TSGs with a posterior probability > 0.5 also include *APC* (0.52). The genes with the highest posterior probability of being associated with bowel cancer alone were *APC* (0.48) and *GAPDH* (0.48).

## Discussion

We used a large exome-sequencing dataset to describe the pattern of gene-based associations across 11 cancer types. For cancers with results not previously reported elsewhere, we found exome-wide significant genes for pancreatic, endometrial, ovarian, esophagus, kidney, and bladder cancer, as well as for malignant melanoma, based on the Wald test. Many of these associations were, however, based on low carrier counts, and the associations were less significant based on the more conservative Firth regression or a likelihood ratio test: of gene-disease associations not previously reported, only *MED9* for melanoma reached exome-wide significance.

Previous studies, such as Backman et al.^16^ and Wang et al.,^28^ have demonstrated the utility of phenome-wide association studies (PheWAS) to identify associations with traits and rare coding variants. Our study highlights additional associations that were not identified in these studies. Our analysis uses a Bayesian approach to estimate the posterior probability of a gene being associated with a cancer, or a pair of cancers, and enables the identification of additional genes to be associated with risk that would have been missed by a simple trait-gene burden test. For example, we identified *ATM* to be associated with 9 cancer pairs with a posterior probability > 0.8 (involving bowel, lung, and esophageal cancer in addition to breast, prostate, ovary, and pancreas), while it was only associated with breast cancer by Backman et al.^16^ and Wang et al.^28^

Our study highlights the potential value of combining information for multiple cancers. We found strong evidence for overlap in susceptibility genes between several cancer pairs. This information can strengthen the evidence for susceptibility to one cancer by borrowing information from the other cancer, in effect improving the prior. Thus, we found the data to be consistent with an essentially complete overlap of susceptibility genes for breast, ovarian, pancreatic, and prostate cancer. While this is not surprising given that several of the known susceptibility genes are associated with several of these cancers (though not to the same extent), the reasons underlying the overlap in susceptibility to these cancers are not well understood, and the results thus suggest avenues for future research.

The results also highlight some additional putative susceptibility genes. Of particular interest was *NHEJ1*, which reached exome-wide significance in ovarian cancer analysis for the Wald test and showed evidence of association with breast cancer. The latter association would have been missed if considered in isolation. *NHEJ1* encodes a DNA repair factor essential for non-homologous end joining (NHEJ). The association for PTVs is consistent with NHEJ being defective in a large proportion of ovarian cancers.^29^ Ovarian cancer cells defective in NHEJ have also been shown to be resistant to PARP inhibition,^29^ which might indicate some relevance for therapeutic approaches. We also found stronger evidence for *BAP1* in the joint analysis of breast and prostate cancer. *BAP1* PTVs have been associated with a range of cancers, including cutaneous and uveal melanoma, kidney cancer, mesothelioma, and basal cell carcinoma. Kidney (*p* = 0.059) and bladder (*p* = 0.047) cancer, as well as ovarian (*p* = 0.033) and pancreatic (*p* = 0.022) cancer, also showed some evidence of association in this dataset (Table S30; Figure S15).

Beyond the breast, ovarian, pancreatic, and prostate associations and the expected overlap between bowel and endometrial cancer, driven by the MMR genes, there were also significant, albeit weaker, overlaps between breast cancer and several other cancers. This is partly driven by *ATM*, which was associated with 9 pairs as mentioned above. The best-fitting models for lung cancer in combination with breast, pancreatic, and prostate cancer are all consistent, with most susceptibility genes for these cancers also being associated with lung cancer, albeit to a smaller extent.

The results confirm the striking enrichment of tumor-suppressor and DNA repair genes among cancer susceptibility genes. Thus, of the 19 genes with a posterior probability > 0.5 in any analyses, 12 are known TSGs and/or involved in DNA repair (including all 11 genes with a posterior probability > 0.8). In terms of the contribution of PTVs to the FRR of each cancer, the proportion was estimated to be greatest for ovarian cancer (46%), followed by endometrial cancer (16.5%). The contribution was estimated to be 0% for kidney and bladder cancer and malignant melanoma. This is clearly inaccurate: *CDKN2A* and *CDK4* (MIM: 123829) PTVs are associated with malignant melanoma,^30^^,^^31^ and *VHL* (MIM: 608537) is associated with kidney cancer.^32^ (*CDKN2A* did reach exome-wide significance for association with melanoma in this dataset using a Wald test, but this is likely to have been exaggerated, as the likelihood ratio *p* value was 0.00041.) *CDK4* had 0 carriers with melanoma, while *VHL* had 1 carrier with kidney cancer, and the OR was non-significant (3.5 [0.49, 25.4], *p* = 0.21). Larger datasets should allow more precise estimates of the associations between these genes and cancer risk to be derived. The UK Biobank has lower incidence rates for some cancers (e.g., kidney and lung), reflecting a “healthy volunteer” bias.^33^ While this is unlikely to result in any significant bias in the association analyses, studies in populations with incidence rates would also improve power. Notwithstanding these uncertainties, the estimated contribution to the FRR, for all cancers, was largely attributable to the known genes. This strongly suggests that the contribution of additional genes is likely to be small and that most “missing” heritability is likely to reflect non-coding variation.

Similar to the breast cancer results reported previously, there was an excess of associations at *p* < 0.001 across the cancers, indicating that further genes should be identifiable in larger datasets. We also note that for many cancers, the sample size is small, resulting in large standard errors and wide confidence intervals for many associated genes. Estimated risk estimates for associated genes not previously established may also be over-estimated due to the “winners curse.”^34^ Further replication in larger datasets will, therefore, be necessary to confirm associations and provide more precise risk estimates for variants in associated genes. Furthermore, our study focused on individuals of European ancestry, and expanding the analysis to other populations will be important and may identify additional associations. Some other large cohorts, e.g., All of Us, could provide a basis for replicating the findings across multiple cancers in the future; All of Us also has more diverse ancestry, with 51% of individuals being of non-European ancestry.^35^ Alternatively, large targeted gene panel studies may be a more feasible option for individual cancers.

Our analyses have some limitations. We restricted our analyses to PTVs. For the known genes, most of the effect is driven by PTVs, and the assumption that PTVs confer similar risks is a plausible simplification. In principle, the analyses could be extended to missense or other coding variants, but this would require the model to be extended to incorporate variation in effect size. Our multicancer analyses have thus far been restricted to pairs of cancers but could logically be extended to larger sets of cancers. This would provide a more rational model, defining a single set of parameters and posterior probabilities. However, it would require a larger number of parameters to be estimated simultaneously. The model also assumed a particular prior distribution of effect sizes, in which a proportion of genes are associated with risk—an example of a spike-and-slab prior.^36^ This is somewhat analogous to the approach used in some GWAS analyses, for example, LDpred2.^37^ The model also only accounts for rare variants associated with an increased risk and does not account for protective alleles. We note, however, that all 47 exome-wide significant associations were positive, so this appears a reasonable simplification given the available data. The model is simplistic and may not reflect the true underlying distribution for all cancers; however, the model provides a systematic approach to identify and rank genes worthy of exploration in larger targeted sequencing and functional experiments.

In conclusion, we have developed an approach to estimate the genome-wide contribution of the burden of rare coding variants to the heritability of cancer, considering 11 cancers in the UK Biobank. We have shown significant clustering of cancer types, including breast, ovarian, prostate, and pancreatic cancer, with a large enrichment of tumor-suppressor and DNA repair genes among cancer susceptibility genes. The estimated contribution to the FRR, for all cancers, was largely attributable to the known genes. This strongly suggests that the contribution of additional genes is likely to be small and that most missing heritability is likely to reflect non-coding variation.

## Data and code availability

Requests for access to UK Biobank data should be made to the UK Biobank access management team (access@ukbiobank.ac.uk). QC filtering of VCF files was performed using vcftools v.0.1.15, bcftools v.1.9, picard v.2.22.2, and plink v.1.90b, as outlined in the material and methods. Variants were annotated using Ensembl VEP v.101 with assembly GRCh38. The code for each software is available at the website of each package. Data manipulation and analysis were performed using R-4.3.3 with the packages clusterProfiler (4.2.2), data.table (1.14.2), dplyr (1.0.9), dbplyr (2.5.0), gtools (3.9.5), HGNChelper (0.8.9), SKAT (2.2.5), tibble (3.2.1), and tidyr (1.3.1). Plots were created using the additional packages ggplot2 (3.5.1) and ggrepel (0.9.5). The code for each of the R packages can be found in their associated vignettes. Burden test results are available for each cancer from the GWAS Catalog (https://www.ebi.ac.uk/gwas/; https://ftp.ebi.ac.uk/pub/databases/gwas/summary_statistics/). The accession numbers for the burden test results reported in this paper are GWAS Catalog: GCST90503274 (pancreatic cancer), GCST90503275 (endometrial cancer), GCST90503276 (ovarian cancer), GCST90503277 (oesophagus cancer), GSCST90503278 (kidney cancer), GCST90503279 (bladder cancer), and GCST90503280 (malignant melanoma).

## Acknowledgments

QC of the UK Biobank sequencing data was funded by the 10.13039/501100000265Medical Research Council (unit programs: MC_UU_12015/2 and MC_UU_00006/2). The research was conducted using the UK Biobank Resource under application no. 28126. N.W. was supported by the International Alliance for Cancer Early Detection, an alliance between 10.13039/501100000289Cancer Research UK (C14478/A29329), the Canary Center at Stanford University, the 10.13039/501100000735University of Cambridge, the 10.13039/100018071OHSU Knight Cancer Institute, 10.13039/501100000765University College London, and the 10.13039/501100000770University of Manchester. J.D. was supported by core funding from the 10.13039/501100018956NIHR Cambridge Biomedical Research Centre (NIHR203312). X.Y. and J.P.T. were supported by 10.13039/501100000289Cancer Research UK (PPRPGM-Nov20\100002 and PRCPJT-May21\100006).

## Author contributions

D.F.E. supervised this work and directed the overall analysis. N.W. performed the statistical analysis. N.W., E.J.G., J.P.T., and J.D.P. developed the bioinformatics and computational pipelines. X.Y. and J.D. acquired data, and X.Y. extracted cancer phenotypes. N.W. and D.F.E. drafted the manuscript. All authors reviewed and approved the paper.

## Declaration of interests

N.W. has been an employee and shareholder of Illumina since October 1, 2024. J.R.B.P. and E.J.G. are employees of Insmed Innovation UK and hold stock/stock options in Insmed, Inc. J.R.B.P. also receives research funding from GSK and engages in paid consultancy for WW International, Inc.

## Web resources

OMIM, http://www.omim.org

## Supplemental information

Document S1. Figures S1–S15, Tables S8–S15, S17–S26, S28, and S29, and supplemental methods

Data S1. Tables S1–S7, S16, S27, and S30

Document S2. Article plus supplemental information
