# Supplementary material for: The contribution of coding variants to the heritability of multiple cancer types using UK Biobank whole-exome sequencing data
Source: Am J Hum Genet. 2025 Mar 11;112(4):903–12. doi: 10.1016/j.ajhg.2025.02.013 (PMC12081281; doi:10.1016/j.ajhg.2025.02.013)
Supplement: Document S1. Figures S1–S15, Tables S8–S15, S17–S26, S28, and S29, and supplemental methods [file mmc1.pdf]

The American Journal of Human Genetics, Volume *112*

Supplemental information

The contribution of coding variants to the heritability of multiple cancer types using UK Biobank whole-exome sequencing data

Naomi Wilcox, Jonathan P. Tyrer, Joe Dennis, Xin Yang, John R.B. Perry, Eugene J. Gardner, and Douglas F. Easton

Supplementary Material

Table of Contents

Supplementary Methods **2**
Individual cancer model 2
Joint cancer model, method 1 5
Joint cancer model, method 2 8
Maximisation 9
Model extension 9

Supplementary Tables **10**

Single cancer heritability results 11

Joint cancer heritability results 18

Supplementary Figures **31**

Pancreatic Cancer 31

Endometrial Cancer 33

Ovarian Cancer 35

Oesophagus Cancer 37

Kidney Cancer 39

Bladder Cancer 41

Malignant Melanoma 43

Supplementary Methods

Individual cancer model

To derive the likelihood function we stratify the number of carriers by sex, family history, and case/control status.

	**FEMALE**					**MALE**				
	Control		Case			Control		Case		
	FH 0	FH 1	FH 0	FH 1		FH 0	FH 1	FH 0	FH 1	
**Non-carrier**	$N_{F0} - n_{F0j}$	$N_{F1} - n_{F1}$	$N_{F2} - n_{F2j}$	$N_{F3} - n_{F3j}$	$N_F - n_F$	$N_{M0} - n_{M0j}$	$N_{M1} - n_{M1j}$	$N_{M2}—$[illegible]	$N_{M3} - n_{M3j}$	$N_M - n_M$
**Carrier**	n_{F0j}	n_{F1j}	n_{F2j}	n_{F3j}	$n_{F0j} + n_{F1j}+n_{F2}$[illegible] $+ n_{F3j} = n_F$	n_{M0j}	n_{M1j}	n_{M2j}	n_{M3j}	$n_{M0j} + n_{M1j}+n_{M2}$[illegible] $+ n_{M3j} = n_M$
	N_{F0}	N_{F1}	N_{F2}	N_{F3}	$N_{F0} + N_{F1} + N_{F2} + N_{F3} = N_F$	N_{M0}	N_{M1}	N_{M2}	N_{M3}	$N_{M0} + N_{M1} + N_{M2} + N_{M3} = N_M$

For each sex, the number of carriers in each stratum can be modelled by a multinomial distribution with a probability mass function:

$$P\left(n_{oj}, n_{1j}, n_{2j}, n_{3j} | n_j, \beta_j\right) = \frac{\prod_{k=0}^{3}\left(N_k \frac{1}{2}\left(ke^{\beta_j} + 2 - k\right)\right)^{n_{kj}}}{\left(\sum_{k=0}^{3} N_k \frac{1}{2}\left(ke^{\beta_j} + 2 - k\right)\right)^{n_j}}$$

Defining $\gamma_{Fk} = log(\frac{N_{Fk}}{N_{F0}})$, $\gamma_{Mk} = log(\frac{N_{Mk}}{N_{M0}})$, and multiplying the probabilities for males and females, this simplifies to:

$$P\left(n_{oj},n_{1j},n_{2j},n_{3j}|n_j,\beta_j\right)=C\frac{\prod_{k=0}^{3}\left(\frac{1}{2}\left(ke^{\beta_j}+2-k\right)\right)^{n_{Fkj}}\prod_{k=0}^{3}\left(\frac{1}{2}\left(ke^{\beta_j}+2-k\right)\right)^{n_{Mkj}}}{\left(\sum_{k=0}^{3}e^{\gamma_{F_k}}\frac{1}{2}\left(ke^{\beta_j}+2-k\right)\right)^{n_{Fj}}\left(\sum_{k=0}^{3}e^{\gamma_{M_k}}\frac{1}{2}\left(ke^{\beta_j}+2-k\right)\right)^{n_{Mj}}}$$

Where $C=\binom{n_{Fj}}{n_{F0j}\quad n_{F1j}\quad n_{F2j}\quad n_{F3j}}\binom{n_{Mj}}{n_{M0j}\quad n_{M1j}\quad n_{M2j}\quad n_{M3j}}e^{\sum_{k=0}^{3}\gamma_{Fk}n_{Fkj}+\sum_{k=0}^{3}\gamma_{Mk}n_{Mkj}}$ is independent of the prior distribution.

The likelihood is integrated over the prior distribution to give the likelihood to be maximised:

$$L(\alpha,\eta)\propto\prod_{j=1}^{J}\int\frac{\prod_{k=0}^{3}\left(\frac{1}{2}\left(ke^{\beta_j}+2-k\right)\right)^{n_{Fkj}}\prod_{k=0}^{3}\left(\frac{1}{2}\left(ke^{\beta_j}+2-k\right)\right)^{n_{Mkj}}}{\left(\sum_{k=0}^{3}e^{\gamma_{F_k}}\frac{1}{2}\left(ke^{\beta_j}+2-k\right)\right)^{n_{Fj}}\left(\sum_{k=0}^{3}e^{\gamma_{M_k}}\frac{1}{2}\left(ke^{\beta_j}+2-k\right)\right)^{n_{Mj}}}f\left(\beta_j|\alpha,\eta\right)d\beta_j$$

Where $f(\beta_j|\alpha,\eta)$ is the prior distribution on β_j.

Writing $L_j\left(\beta_j\right)=$

$$\left(\sum_{k=0}^{3}e^{\gamma_{F_k}}\right)^{n_{Fj}}\left(\sum_{k=0}^{3}e^{\gamma_{M_k}}\right)^{n_{Mj}}\frac{\prod_{k=0}^{3}\left(\frac{1}{2}\left(ke^{\beta_j}+2-k\right)\right)^{n_{Fkj}}\prod_{k=0}^{3}\left(\frac{1}{2}(ke^{\beta_j}+2-k)\right)^{n_{Mkj}}}{\left(\sum_{k=0}^{3}e^{\gamma_{F_k}}\frac{1}{2}\left(\left(ke^{\beta_j}+2-k\right)\right)\right)^{n_{Fj}}\left(\sum_{k=0}^{3}e^{\gamma_{M_k}}\frac{1}{2}(ke^{\beta_j}+2-k)\right)^{n_{Mj}}}$$

$L(\alpha,\eta)\propto\prod_{j=1}^{J}\int L_j\left(\beta_j\right)f\left(\beta_j|\alpha,\eta\right)d\beta_j=\prod_{j=1}^{J}\left(1-\alpha+\alpha\int L_j\left(\beta_j\right)g\left(\beta_j|\eta\right)d\beta_j\right)=\prod_{j=1}^{J}(1-\alpha+\alpha L_{*j})$.

The posterior probability a gene is associated, given optimised estimates of $\alpha$and η is: $P\left(\beta_j\middle|Data\right)=\frac{\alpha\int L_j(\beta_j)g\left(\beta_j|\eta\right)d\beta_j}{1-\alpha+\alpha\int L_j(\beta_j)g\left(\beta_j|\eta\right)d\beta_j}=\frac{\alpha L_{*j}}{1-\alpha+\alpha L_{*j}}$.

The posterior mean β_j is: $\frac{\int\beta_j L_j(\beta_j)g\left(\beta_j|\eta\right)d\beta_j}{L_{*j}}$.

And the posterior mean relative risk e^{β_j} is: $\frac{\int e^{\beta_j}L_j(\beta_j)g\left(\beta_j|\eta\right)d\beta_j}{L_{*j}}$.

For gene j with aggregate PTV frequency, p_j, associated with relative risk e^{β_j}, the FRR is $\lambda_j = 1 + \frac{p_j(e^{\beta_j}-1)^2}{(2p_j\left(e^{\beta_j}-1\right)+1)^2}$

Using control and case data, we estimate the allele frequency based on the posterior distribution of the relative risk: $p_{Bj}(\beta_j) = \frac{n_{F0j}+n_{F1j}+n_{F2j}+n_{F3j}}{2(N_{F0}+N_{F1}+e^{\beta_j}(N_{F2}+N_{F3})}$.

Hence $\lambda_{jB} = 1 + \frac{\alpha}{1-\alpha+\alpha L_{*j}} \int L_j(\beta_j) g\left(\beta_j|\eta\right) \frac{p_{jB}(\beta_j)(e^{p_{jB}(\beta_j)}-1)^2}{(2p_{jB}(\beta_j)\left(e^{p_{jB}(\beta_j)}-1\right)+1)^2} d\beta_j$

The total FRR over all genes, assuming an additive model, is given by: $\hat{\lambda}_{TOT} = 1 + \sum_{j=1}^{J}(\lambda_j - 1)$.

Assuming that the PTVs combine multiplicatively with other genetic or familial factors, and an overall FRR of 2, the percentage contribution of each gene to the overall FRR is: $100 \times \frac{log(\hat{\lambda}_j)}{log\ (2)}$ and the total contribution of PTVs in all genes is: $100 \times \frac{log(\hat{\lambda}_{TOT}\ )}{log\ (2)}$.

These equations simplify if there is no recorded family history information. In this case, the multinomial distribution simplifies to a binomial distribution. We set $N_{F1} = N_{F3} = N_{M1} = N_{M3} = 0$. Therefore for each sex: $P\left(n_{oj}, n_{2j}|n_j, \beta_j\right) \propto \frac{(N_0\ )^{n_{0j}}\left(N_2 e^{\beta_j}\ \right)^{n_{2j}}}{\left(N_0+N_2 e^{\beta_j}\ \right)^{n_j}}$

Defining $\gamma_{F2} = log(\frac{N_{F2}}{N_{F0}})$, $\gamma_{M2} = log(\frac{N_{M2}}{N_{M0}})$, as above, and multiplying the probabilities for males and females, this simplifies to:

$P\left(n_{oj}, n_{2j}|n_j, \beta_j\right) = C \frac{\left(e^{\gamma_{F2}} e^{\beta_j}\ \right)^{n_{F2j}} \left(e^{\gamma_{M2}} e^{\beta_j}\ \right)^{n_{M2j}}}{\left(1+e^{\gamma_{F2}} e^{\beta_j}\right)^{n_{Fj}} \left(1+e^{\gamma_{M2}} e^{\beta_j}\right)^{n_{Mj}}}$. Here C=$\binom{n_{Fj}}{n_{F2j}}\binom{n_{Mj}}{n_{M2j}}$.

Therefore, $L(\alpha,\eta) \propto \prod_{j=1}^{J} \int \frac{(N_{F0})^{n_{F0j}}\left(N_{F2}e^{\beta_j}\right)^{n_{F2j}}(N_{M0})^{n_{M0j}}\left(N_{M2}e^{\beta_j}\right)^{n_{M2j}}}{\left(1+e^{\gamma_{F2}}e^{\beta_j}\right)^{n_{Fj}}\left(1+e^{\gamma_{M2}}e^{\beta_j}\right)^{n_{Mj}}} f\left(\beta_j|\alpha,\eta\right) d\beta_j$

Joint cancer model, method 1

We now consider two cancers and assume distributions for effect sizes in which a proportion, α_1, of genes are associated with cancer 1, and α_2 are associated with cancer 2. The log-relative risk for cancer 1, β_1, has a density of the form $f(\beta_1|\alpha_1,\eta_1)$ and the log-relative risk for cancer 2, β_2, has a density of the form $f(\beta_2|\alpha_2,\eta_2)$. We assume that the prior probabilities can be correlated (i.e., the probability that a gene is a risk for cancer 2 is dependent on whether it is a gene for cancer 1).

There are now 4 combinations for a gene j:

		$\boldsymbol{Cancer\ 1, \beta_{1j}}$	
		**0 (not associated)** $w.p\ (\mathbf{1}-\boldsymbol{\alpha_1})$	$\boldsymbol{g(\beta_1|\eta)}$ **(associated)** $w.p\ \boldsymbol{\alpha_1}$
$\boldsymbol{Cancer\ 2, \beta_{2j}}$	**0 (not associated)** $w.p\ (\mathbf{1}-\boldsymbol{\alpha_2})$	(0,0) $w.p\ (1-\alpha_{10}-\alpha_{01}-\alpha_{11})$	$(g(\beta_1|\eta_1),0)$ $w.p\ \alpha_{10}$
	$\boldsymbol{g(\beta_2|\eta_2)}$ **(associated)** $w.p\ \boldsymbol{\alpha_2}$	$(0, g(\beta_2|\eta_2))$ $w.p\ \alpha_{01}$	$(g(\beta_1|\eta_1), g(\beta_2|\eta_2))$ $w.p\ \alpha_{11}$

i.e.

$$(\beta_1,\beta_2) \sim \begin{cases} (0,0)\ w.p.\ (1-\alpha_{10}-\alpha_{01}-\alpha_{11}) \\ (g(\beta_1|\eta_1),0)\ w.p.\ \alpha_{10} \\ (0, g(\beta_2|\eta_2))\ w.p\ \alpha_{01} \\ (g(\beta_1|\eta_1), g(\beta_2|\eta_2))\ w.p.\ \alpha_{11} \end{cases}$$

Where $g(\beta_1|\eta_1) \sim \eta_1 \exp(-\eta_1\beta_1)$, $g(\beta_2|\eta_2) \sim \eta_2 \exp(-\eta_2\beta_2)$,

For simplicity, we assume that the effect sizes β_1 and β_2 are uncorrelated.

There are five parameters to estimate: α_{10}, α_{01}, α_{11}, η_1, and η_2.

We can calculate $\alpha_1 = \alpha_{10} + \alpha_{11}$ and $\alpha_2 = \alpha_{01} + \alpha_{11}$. We note $\alpha_1, \alpha_2, \eta_1$, and $\eta_2$calculated here may differ to the values from optimising the individual cancer models.

The odds ratio $\alpha_{11}(1 - \alpha_{10} - \alpha_{01} - \alpha_{11})/(\alpha_{10}\alpha_{01})$ represents the degree of enrichment of susceptibility to cancer 2 given cancer 1 (or vice versa).

The likelihood can be written as:

$$L(\alpha_{10}, \alpha_{01}, \alpha_{11}, \eta_1, \eta_2) \propto \prod_{j=1}^{J} \iint L_j(\beta_{1j}, \beta_{2j}) f(\beta_{1j}, \beta_{2j} | \alpha_{10}, \alpha_{01}, \alpha_{11}, \eta_1, \eta_2)\, d\beta_{1j} d\beta_{2j}$$

$$= \prod_{j=1}^{J} \int L_j(\beta_{1j}) f(\beta_{1j}|\alpha_1\eta) d\beta_{1j} \int L_j(\beta_{2j}) f(\beta_{2j}|\alpha_2\eta) d\beta_{2j}$$

$$= \prod_{j=1}^{J} \Big((1 - \alpha_{10} - \alpha_{01} - \alpha_{11}) + \alpha_{10} \int L_{j1}(\beta_{1j}) g(\beta_{1j}|\eta_1) d\beta_{1j}$$

$$+ \alpha_{01} \int L_{j2}(\beta_{2j}) g(\beta_{2j}|\eta_2) d\beta_{1j}$$

$$+ \alpha_{11} \int L_j(\beta_{1j}) g(\beta_{1j}|\eta_1) d\beta_{1j} \int L_j(\beta_{2j}) g(\beta_{2j}|\eta_2) d\beta_{2j} \Big) =$$

$$= \prod_{j=1}^{J} \Big((1 - \alpha_{10} - \alpha_{01} - \alpha_{11}) + \alpha_{10} L_{*j1}(\eta_1) + \alpha_{01} L_{*j2}(\eta_2)$$

$$+ \alpha_{11} L_{*j1}(\eta_1) L_{*j2}(\eta_2) \Big)$$

The posterior probability a gene is associated with cancer 1 given the estimates α_{10}, α_{01}, α_{11}, $\eta_1$and η_2, is: $\mathrm{P(Risk\ gene\ for\ cancer\ 1|Data)} = \frac{\alpha_{10}L_{1*j} + \alpha_{11}L_{1*j}L_{2*j}}{(1-\alpha_{10}-\alpha_{01}-\alpha_{11}) + \alpha_{10}L_{1*j} + \alpha_{01}L_{2*j} + \alpha_{11}L_{1*j}L_{2*j}}$, and similarly for cancer 2. L_{1*} and L_{2*} both depend on $\eta_1$and η_2 respectively.

The posterior probability a gene is associated with both cancers is:

$$\mathrm{P(Risk\ gene\ for\ both\ cancers\ \ |Data)} = \frac{\alpha_{11}\mathrm{L}_{1*\mathrm{j}}\mathrm{L}_{2*\mathrm{j}}}{(1-\alpha_{10}-\alpha_{01}-\alpha_{11})+\alpha_{10}\mathrm{L}_{1*\mathrm{j}}+\alpha_{01}\mathrm{L}_{2*\mathrm{j}}+\alpha_{11}\mathrm{L}_{1*\mathrm{j}}\mathrm{L}_{2*\mathrm{j}}}$$

We can calculate a likelihood ratio test to test for enrichment of susceptibility to cancer 2 given cancer 1 (or vice versa), by comparing the joint likelihood above to the joint likelihood when the cancers are independent.

		Cancer 1, $\boldsymbol{\beta_{1j}}$	
		**0 (not associated)** $w.p\ (\mathbf{1}-\boldsymbol{\alpha_1})$	$\boldsymbol{g(\beta_1\|\eta_1)}$ **(associated)** $w.p\ \boldsymbol{\alpha_1}$
$Cancer\ 2, \beta_{2j}$	**0 (not associated)** $w.p\ (\mathbf{1}-\boldsymbol{\alpha_2})$	(0,0) $w.p\ (1-\alpha_{10}-\alpha_{01}-\alpha_{11}) = (1-\alpha_1)\ (1-\alpha_2)$	$(g(\beta_1\|\eta_1)$,0) $w.p\ \alpha_{10}$=$\alpha_1(1-\alpha_2)$
	$\boldsymbol{g(\beta_2\|\eta_2)}$ **(associated)** $w.p\ \boldsymbol{\alpha_2}$	(0, $g(\beta_2\|\eta_2)$) $w.p\ \alpha_{01} = \alpha_2(1-\alpha_1)$	$(g(\beta_1\|\eta_1), g(\beta_2\|\eta_2))$ $w.p\ \alpha_{11}$=$\alpha_1\alpha_2$

The odds ratio $\frac{\alpha_{11}(1-\alpha_{10}-\alpha_{01}-\alpha_{11})}{\alpha_{10}\alpha_{01}} = \frac{\alpha_1\alpha_2(1-\alpha_1)\ (1-\alpha_2)}{\alpha_1\alpha_2(1-\alpha_1)\ (1-\alpha_2)} = 1$, and α_{11}=$\alpha_1\alpha_2$. Therefore:

$$L(\alpha_{10},\alpha_{01},\alpha_{11},\eta_1,\eta_2) = \prod_{j=1}^{J}\left(\left(\frac{\alpha_{01}\alpha_{10}}{\alpha_{11}}\right)+\alpha_{10}L_{*j1}(\eta_1)+\alpha_{01}L_{*j2}(\eta_2)+\alpha_{11}L_{*j1}(\eta_1)L_{*j2}(\eta_2)\right)$$

$$= \prod_{j=1}^{J}\frac{1}{\alpha_{11}}\left((\alpha_{01}\alpha_{10})+\alpha_{11}\alpha_{10}L_{*j1}(\eta_1)+\alpha_{11}\alpha_{01}L_{*j2}(\eta_2)+\alpha_{11}{}^2L_{*j1}(\eta_1)L_{*j2}(\eta_2)\right)$$

$$= \prod_{j=1}^{J}\frac{1}{\alpha_{11}}\left(\alpha_{01}+\alpha_{11}L_{*j1}(\eta_1)\right)\left(\alpha_{10}+\alpha_{11}L_{*j2}(\eta_2)\right)$$

$$= \prod_{j=1}^{J}\frac{1}{\alpha_1\alpha_2}\left(\alpha_2(1-\alpha_1)+\alpha_1\alpha_2L_{*j1}(\eta_1)\right)\left(\alpha_1(1-\alpha_2)+\alpha_1\alpha_2L_{*j2}(\eta_2)\right) =$$

$$= \prod_{j=1}^{J}(1-\alpha_1+\alpha_1L_{*j1}(\eta_1))(1-\alpha_2+\alpha_2L_{*j1}(\eta_2))$$

i.e., the likelihood simplifies to the product of the individual cancer likelihoods, assuming consistent $\eta_1$and η_2. Therefore, the likelihood ratio test is a comparison of the joint log-likelihood to the sum of the log-likelihoods of the separate models, using the chi-square distribution with 1 degree of freedom (df).

Joint cancer model, method 2

For some cancer pairs method 1 is difficult to optimise and we therefore consider an alternative method where we fix the marginal parameters α_1 and α_2 and estimate $\alpha_3 = P(C_2|C_1)$, η_1 and η_2. We then calculate $\alpha_{01}, \alpha_{10}, \alpha_{11}$ and α_{00}:

$$\alpha_{11} = P(C_1 \cap \ C_2) = P(C_2|C_1)P(C_1) = P(C_1|C_2)P(C_2) = \alpha_1\alpha_3$$

$$\alpha_{01} = P(\sim C_1 \cap \ C_2) = P(C_2|\sim C_1)P(\sim C_1) = P(\sim C_1|C_2)P(C_2) = \alpha_2\left(1 - \frac{\alpha_1\alpha_3}{\alpha_2}\right) = \alpha_2 - \alpha_1\alpha_3$$

$$\alpha_{10} = P(C_1 \cap \ \sim C_2) = \alpha_{11} = P(\sim C_2|C_1)P(C_1) = P(C_1|\sim C_2)P(\sim C_2) = \alpha_1(1 - \alpha_3)$$

$$\alpha_{00} = P(\sim C_1 \cap \ \sim C_2) = 1 - \alpha_1 - \alpha_2 + \alpha_1\alpha_3$$

These values should be similar to the values from joint model method 1 if the values of η_1, η_2, α_1 and α_2 are similar.

We set $P(C_2|C_1)$= α_3, and can calculate $P(C_1|C_2) = \frac{\alpha_1\alpha_3}{\alpha_2}$

The equivalent likelihood ratio test is testing $\alpha_3 = \alpha_2$, i.e., $P(C_2|C_1) = P(C_2)$.

We note that $\alpha_1\alpha_3 \leq \alpha_1$ and $\alpha_1\alpha_3 \leq \alpha_2$, i.e., $\alpha_3 \leq min\left(\frac{\alpha_2}{\alpha_1}, 1\right)$.

It therefore makes sense to fit the model with cancer 1 being the cancer with the greatest proportion of genes to be risk associated so that the upper bound of α_3 can consistently be set at 1. If not, and α_3 is estimated to be $\geq \frac{\alpha_2}{\alpha_1}$ then $P(C_1|C_2)$.

This method was used for bowel-endometrial and lung-ovarian cancer.

Maximisation

We maximise each log-likelihood to estimate the parameters using the L-BFGS-B optimisation algorithm in the optim package on R. This is a method by Byrd et. Al, 1995 for large non-linear optimisation problems which allows for multivariate estimation and parameter box constraints, i.e., $0 < \alpha < 1$[25].

Model extension

The joint model method 1 could be extended to more than 2 cancers e.g., breast, prostate and ovarian cancer which have significant overlap. For 3 cancers we would need to estimate 7 α's and 3 η's:

$$L(\alpha_{100}, \alpha_{010}, \alpha_{001}, \alpha_{110}, \alpha_{101}, \alpha_{011}, \alpha_{111}, \eta_1, \eta_2, \eta_3)$$

$$\propto \prod_{j=1}^{J} \Big((1 - \alpha_{100} - \alpha_{010} - \alpha_{001} - \alpha_{110} - \alpha_{101} - \alpha_{011} - \alpha_{111}) + \alpha_{100} L_{*j1}(\eta_1)$$

$$+ \alpha_{010} L_{*j2}(\eta_2) + \alpha_{001} L_{*j3}(\eta_3) + \alpha_{110} L_{*j1}(\eta_1) L_{*j2}(\eta_2) + \alpha_{101} L_{*j1}(\eta_1) L_{*j3}(\eta_2)$$

$$+ \alpha_{011} L_{*j2}(\eta_1) L_{*j3}(\eta_2) + \alpha_{111} L_{*j1}(\eta_1) L_{*j2}(\eta_2) L_{*j3}(\eta_2) \Big)$$

To account for n cancers, we would need to estimate 2^n-1 α's and n η's.

Supplementary Tables

LRT p-values

Table S8 | A comparison of Wald test, Firth regression, and LRT P-values for genes reaching exome-wide significance in the Wald test and with case carriers ≤5.

Cancer	Gene	Control		Case		OR (CI)	P-value		
		Non-carriers	carriers	Non-carriers	carriers		Wald	Firth regression	LRT
Pancreatic	*MEN1*	418224	2	1079	2	429 (57.4, 3200)	3.46E-09	4.17E-06	1.66E-05
	RCN2	418188	38	1078	3	31 (9.53, 101)	1.17E-08	2.32E-05	0.000136
	YPEL3	418215	11	1079	2	78 (17.2, 355)	1.73E-08	6.32E-05	0.000292
	SMC2	418177	49	1078	3	24.9 (7.74, 80.1)	7.03E-08	4.93E-05	0.000258
	GNG10	418205	21	1079	2	39.4 (9.15, 169)	8.05E-07	0.00022	0.00113
	ZNF461	418201	25	1079	2	32.8 (7.71, 139)	2.26E-06	0.000269	0.00164
Endometrial	*MLH1*	225410	9	1971	3	40.3 (10.9, 149)	3.24E-08	1.77E-05	8.2E-05
	ACRV1	225388	31	1970	4	14.6 (5.14, 41.4)	4.77E-07	6.21E-05	0.000225
	STK32C	225403	16	1971	3	21.5 (6.25, 74.2)	1.15E-06	0.000112	0.000466
	PSMC6	225417	2	1972	2	115 (16, 825)	2.37E-06	5.70E-05	0.000239
Ovarian	*IVD*	225957	64	1367	5	12.9 (5.19, 32.2)	3.84E-08	1.66E-05	6.26E-05
	JAML	226000	21	1369	3	24 (7.15, 80.7)	2.75E-07	6.72E-05	0.000322
	KCNAB2	226016	5	1370	2	68.9 (13.2, 360)	5.17E-07	8.77E-05	0.000461
	ZFP14	226016	5	1370	2	64.8 (12.5, 337)	6.92E-07	8.43E-05	0.000521
	TMEM163	226015	6	1370	2	54.1 (10.9, 269)	1.09E-06	1.20E-04	0.000716
	TMEM167A	226014	7	1370	2	47.2 (9.76, 228)	1.64E-06	1.48E-04	0.000916
	NHEJ1	225993	28	1369	3	17.7 (5.36, 58.2)	2.37E-06	1.76E-04	0.000782
Oesophagus	*KNL1*	418238	67	998	4	23.7 (8.54, 65.6)	1.17E-09	6.14E-06	3.09E-05
	IRF2BP2	418282	23	1000	2	36 (8.31, 156)	1.66E-06	0.000220	0.00138
Kidney	*FGL2*	417654	12	1639	2	49.3 (10.8, 225)	4.66E-07	0.000110	0.000768
	TTC9	417627	39	1638	3	20.4 (6.25, 66.5)	5.78E-07	0.000107	0.000493
	EXOC7	417536	130	1636	5	9.53 (3.88, 23.4)	8.45E-07	7.80E-05	0.000251
	NCK2	417652	14	1639	2	39.8 (8.94, 178)	1.34E-06	0.000188	0.00117
	TMEM174	417653	13	1639	2	39.9 (8.86, 180)	1.60E-06	0.000198	0.00118
Bladder	*DLX2*	417873	9	1423	2	99.5 (19.9, 498)	2.13E-08	3.30E-05	0.00205
	ZNF506	417873	9	1423	2	60.6 (12.4, 296)	3.98E-07	9.10E-05	0.000555
	CDCP2	417714	168	1420	5	9.81 (4, 24.1)	6.25E-07	6.72E-05	0.000220
	TMEM222	417836	46	1422	3	19.5 (5.95, 63.6)	9.11E-07	0.000129	0.000572
	KDM1A	417833	49	1422	3	18.3 (5.62, 59.7)	1.41E-06	0.000156	0.000681
	ARHGEF6	227079	2	311	1	400 (35, 4570)	1.43E-06	0.000176	0.00203
	HR	417711	171	1420	5	9.01 (3.68, 22.1)	1.53E-06	0.000104	0.000326
	NLRP10	417879	3	1424	1	293 (28.9, 2970)	1.54E-06	0.000398	0.00269
Malignant Melanoma	*MED9*	415117	11	4175	4	36.1 (11.5, 114)	8.62E-10	2.16E-06	9.20E-06
	MRPL44	415083	45	4174	5	11.6 (4.59, 29.3)	2.17E-07	3.46E-05	0.000113
	CDKN2A	415115	13	4176	3	23 (6.53, 80.8)	1.04E-06	9.56E-05	0.000413
	KLHL32	415058	70	4174	5	7.14 (2.88, 17.7)	2.24E-05	0.000371	0.00102

Single cancer heritability results

Table S9 | Heritability results for breast cancer, including the posterior probability of being disease associated, posterior mean effect sizes and the proportion of the familial relative risk (FRR) explained. Genes listed have posterior probability >0.1. This analysis uses females and males, incorporating family history data, and adjusts for CNV frequency. Results are sorted by descending posterior probability.

Gene	Posterior probability	Posterior mean β	Posterior mean e^{β}	λ	%FRR
BRCA1	1.000	2.10	8.19	1.02084	2.976
BRCA2	1.000	1.70	5.51	1.02525	3.598
CHEK2	1.000	0.85	2.35	1.00518	0.746
PALB2	1.000	1.34	3.81	1.00632	0.910
ATM	1.000	0.78	2.18	1.00176	0.254
MAP3K1	0.983	1.45	4.44	1.00076	0.109
BAP1	0.280	1.22	3.61	1.00011	0.015
PCDHGB3	0.272	0.37	1.46	1.00007	0.010
COL12A1	0.188	0.76	2.20	1.00005	0.008
RNF112	0.177	0.84	2.41	1.00005	0.007
BARD1	0.144	0.65	1.96	1.00007	0.010
KLK4	0.118	0.66	1.97	1.00003	0.004
CYBC1	0.116	1.92	8.22	1.00010	0.014
			**All genes**	**1.06300**	**8.821**

Table S10 | Heritability results for bowel cancer, including the posterior probability of being disease associated, posterior mean effect sizes and the proportion of the familial relative risk (FRR) explained. Genes listed have posterior probability >0.1. This analysis uses females and males, incorporating family history data, and adjusts for CNV frequency. Results are sorted by descending posterior probability.

Gene	Posterior probability	Posterior mean β	Posterior mean e^{β}	λ	%FRR
MSH2	1.000	1.89	6.71	1.00614	0.884
MSH6	1.000	1.71	5.57	1.00769	1.105
MLH1	1.000	2.86	17.56	1.02583	3.678
APC	1.000	2.39	11.27	1.00465	0.670
GAPDH	0.523	1.18	3.38	1.00034	0.049
FLCN	0.278	0.99	2.77	1.00021	0.030
			**All genes**	1.04750	6.697

Table S11 | Heritability results for prostate cancer, including the posterior probability of being disease associated, posterior mean effect sizes and the proportion of the familial relative risk (FRR) explained. Genes listed have posterior probability >0.1. This analysis uses females and males, incorporating family history data, and adjusts for CNV frequency. Results are sorted by descending posterior probability.

Gene	Posterior probability	Posterior mean β	Posterior mean e^{β}	λ	%FRR
BRCA2	1.000	0.72	2.05	1.00166	0.2399
CHEK2	1.000	0.44	1.56	1.00100	0.1441
ATM	0.999	0.54	1.73	1.00067	0.0970
PPP5C	0.388	0.46	1.61	1.00008	0.0109
INVS	0.338	0.39	1.50	1.00006	0.0084
BET1	0.272	0.44	1.58	1.00004	0.0060
PNLDC1	0.265	0.42	1.54	1.00004	0.0061
PPEF2	0.247	0.25	1.30	1.00004	0.0055
CHID1	0.221	0.37	1.47	1.00003	0.0041
MICB	0.219	0.32	1.39	1.00003	0.0041
MYH7	0.217	0.42	1.55	1.00003	0.0043
TRMT44	0.194	0.26	1.30	1.00003	0.0048
FSIP2	0.184	0.19	1.22	1.00004	0.0051
SNTG2	0.161	0.29	1.36	1.00002	0.0028
TMC2	0.154	0.34	1.42	1.00003	0.0050
WDR59	0.153	0.33	1.41	1.00003	0.0045
LYST	0.152	0.38	1.50	1.00002	0.0027
GEMIN2	0.148	0.66	2.11	1.00001	0.0019
MFSD8	0.135	0.49	1.71	1.00003	0.0041
C9orf50	0.128	0.53	1.81	1.00001	0.0013
NAA11	0.126	0.26	1.32	1.00001	0.0018
CCDC188	0.121	0.29	1.35	1.00001	0.0018
OAT	0.119	0.32	1.40	1.00001	0.0019
BSCL2	0.119	0.48	1.68	1.00001	0.0014
BEND5	0.118	0.39	1.52	1.00001	0.0013
PABPN1	0.117	0.34	1.44	1.00001	0.0014
MAP3K19	0.115	0.26	1.31	1.00001	0.0017
FOXR1	0.111	0.38	1.51	1.00001	0.0010
SSNA1	0.111	0.29	1.36	1.00001	0.0014
FANCM	0.107	0.17	1.19	1.00001	0.0018
OSGIN1	0.107	0.56	1.90	1.00001	0.0014
CNPY2	0.106	0.39	1.53	1.00001	0.0012
SORD	0.105	0.20	1.22	1.00001	0.0016
DPH1	0.104	0.27	1.32	1.00001	0.0013
DEPDC4	0.103	0.23	1.27	1.00001	0.0014
SPG7	0.103	0.19	1.22	1.00001	0.0015
ATP8B4	0.101	0.26	1.30	1.00001	0.0019
ADAM15	0.101	0.27	1.33	1.00001	0.0014
			**All genes**	1.00751	1.0798

Table S12 | Heritability results for lung cancer, including the posterior probability of being disease associated, posterior mean effect sizes and the proportion of the familial relative risk (FRR) explained. Genes listed have posterior probability >0.1. This analysis uses females and males, incorporating family history data, and adjusts for CNV frequency. Results are sorted by descending posterior probability.

Gene	Posterior probability	Posterior mean β	Posterior mean e^{β}	λ	%FRR
ATM	0.187	0.36	1.44	1.000055	0.00799
			**All genes**	1.000649	0.09354

Table S13 | Heritability results for pancreatic cancer, including the posterior probability of being disease associated, posterior mean effect sizes and the proportion of the familial relative risk (FRR) explained. Genes listed have posterior probability >0.1. This analysis uses females and males and adjusts for CNV frequency. Results are sorted by descending posterior probability.

Gene	Posterior probability	Posterior mean β	Posterior mean e^{β}	λ	%FRR
ATM	1.000	1.608	5.188	1.024699086	3.520
SEC14L3	0.491	1.292	4.073	1.003474889	0.500
LTV1	0.377	0.915	2.662	1.001748643	0.252
MROH6	0.239	0.887	2.631	1.000861016	0.124
MAN2A2	0.152	0.607	1.916	1.000408525	0.059
ALDH1L1	0.134	0.924	2.878	1.000642404	0.093
BRCA2	0.127	0.653	2.035	1.000302181	0.044
PCNT	0.121	0.725	2.232	1.000320685	0.046
RCN2	0.117	1.264	4.838	1.000593978	0.086
PKHD1	0.113	0.659	2.060	1.000256601	0.037
CYP20A1	0.111	0.965	3.131	1.000596335	0.086
SMC2	0.109	1.202	4.499	1.000741102	0.107
CEACAM4	0.108	0.950	3.072	1.000499133	0.072
SMTNL1	0.107	0.819	2.550	1.000219483	0.032
			**All genes**	1.0762	32.930

Table S14 | Heritability results for oesophagus cancer, including the posterior probability of being disease associated, posterior mean effect sizes and the proportion of the familial relative risk (FRR) explained. Genes listed have posterior probability >0.1. This analysis uses females and males and adjusts for CNV frequency. Results are sorted by descending posterior probability.

Gene	Posterior probability	Posterior mean β	Posterior mean e^{β}	λ	%FRR
NLRP12	0.599	0.89	2.57	1.00310	0.446
ATM	0.543	0.95	2.79	1.00302	0.435
ZGRF1	0.267	0.65	2.03	1.00078	0.113
KNL1	0.213	1.19	4.35	1.00051	0.074
CAMKMT	0.178	0.85	2.72	1.00053	0.077
CFTR	0.159	0.57	1.87	1.00031	0.045
FGF11	0.151	0.76	2.43	1.00024	0.035
PCDHA8	0.126	0.45	1.62	1.00020	0.029
VPS13A	0.122	0.60	1.97	1.00018	0.026
RTN4IP1	0.121	0.75	2.48	1.00016	0.024
TMPRSS11D	0.120	0.59	1.96	1.00016	0.022
METTL24	0.114	0.58	1.92	1.00015	0.022
DTHD1	0.111	0.70	2.31	1.00011	0.017
DFFA	0.110	0.70	2.31	1.00019	0.028
CTSF	0.106	0.68	2.24	1.00011	0.016
DIS3	0.103	0.54	1.84	1.00013	0.019
			**All genes**	1.04367	6.166

Table S15 | Heritability results for endometrial cancer, including the posterior probability of being disease associated, posterior mean effect sizes and the proportion of the familial relative risk (FRR) explained. Genes listed have posterior probability >0.1. This analysis uses females only and adjusts for CNV frequency. Results are sorted by descending posterior probability.

Gene	Posterior probability	Posterior mean β	Posterior mean e^{β}	λ	%FRR
MSH6	1.000	2.94	19.01	1.1070	14.666
			**All genes**	1.1214	16.526

Joint cancer heritability results

Table S17 | Optimisation results for cancer pairs. This includes the method used (method 1 or method 2 in methods), and optimised values of alpha10=P(C1 n C2'), alpha01=P(C1' n C2), alpha11=P(C1 n C2), eta1 and eta2, as well as the p-value from the likelihood ratio test comparing this model to the model where the cancers are independent.

Cancer 1	Cancer 2	Method	alpha10	alpha01	alpha11	eta1	eta2	LRT
**Breast**	**Prostate**	1	0.00000	0.00000	0.00220	1.49	2.51	1.53E-09
**Breast**	**Ovarian**	1	0.00000	0.00000	0.00270	1.77	1.23	2.12E-08
**Bowel**	**Endom**	2	0.00020	0.00000	0.00140	1.13	0.93	3.01E-08
**Breast**	**Pancreas**	1	0.00000	0.00000	0.00230	1.62	1.57	0.0000230
**Prostate**	**Ovarian**	1	0.00000	0.00340	0.01700	6.04	2.06	0.000151
**Prostate**	**Pancreas**	1	0.00000	0.00000	0.00840	4.56	2.17	0.000277
**Breast**	**Lung**	1	0.00013	0.00026	0.00150	1.52	6.00	0.00144
**Breast**	**Bowel**	1	0.00053	0.00027	0.00160	1.51	1.00	0.00351
**Pancreas**	**Ovarian**	1	0.00000	0.00000	0.03000	3.09	2.25	0.00540
**Breast**	**Oesophagus**	1	0.00000	0.00980	0.00240	1.64	2.95	0.00622
**Lung**	**Pancreas**	1	0.00000	0.00000	0.00460	5.93	2.05	0.00693
**Bowel**	**Ovarian**	1	0.00000	0.00000	0.00290	1.52	1.19	0.0102
**Prostate**	**Oesophagus**	1	0.00000	0.00000	0.01400	5.49	2.84	0.0106
**Pancreas**	**Oesophagus**	1	0.00000	0.00000	0.00750	2.28	2.37	0.0115
**Lung**	**Prostate**	1	0.00002	0.00002	0.00250	6.00	2.01	0.0161
**Endom**	**Ovarian**	1	0.00000	0.00000	0.00390	1.51	1.28	0.0213
**Bowel**	**Pancreas**	1	0.00000	0.00000	0.00220	1.40	1.68	0.0288
**Lung**	**Oesophagus**	1	0.00000	0.00000	0.00590	5.85	2.28	0.0339
**Bowel**	**Lung**	1	0.00000	0.00000	0.00260	1.49	6.00	0.0602
**Bowel**	**Prostate**	1	0.00000	0.00000	0.00340	1.83	2.93	0.112
**Oesophagus**	**Ovarian**	1	0.00000	0.01700	0.02600	3.36	2.51	0.311
**Lung**	**Endom**	1	0.00050	0.00005	0.00200	6.00	1.50	0.399
**Bowel**	**Oesophagus**	1	0.00000	0.01300	0.00230	1.46	2.94	0.460
**Prostate**	**Endom**	1	0.01300	0.00000	0.00430	5.76	1.91	0.498
**Lung**	**Ovarian**	2	0.00000	0.03350	0.00350	5.43	2.41	0.602
**Breast**	**Endom**	1	0.00098	0.00180	0.00150	1.52	1.51	0.639
**Pancreas**	**Endom**	1	0.00270	0.00000	0.00150	1.52	1.51	1.00
**Oesophagus**	**Endom**	1	0.02400	0.00000	0.00150	3.12	1.27	1.00

Table S18 |Genes with posterior>0.8 for being associated with both cancer 1 and cancer 2 for at least 1 cancer pair. The cancer pairs columns are the cancer pairs which had posterior probability >0.8 of the gene being associated with both cancer 1 and cancer 2.

Gene	Cancer pairs
APC	Breast-Bowel, Bowel-Lung, Bowel-Ovarian, Bowel-Pancreas, Bowel-Oesophagus
ATM	Breast-Bowel, Breast-Lung, Breast-Oesophagus, Breast-Ovarian, Breast-Pancreas, Breast-Prostate, Lung-Pancreas, Pancreas-Ovarian, Prostate-Ovarian, Prostate-Pancreas, Bowel-Lung, Bowel-Pancreas, Lung-Oesophagus, Lung-Prostate, Pancreas-Oesophagus, Prostate-Oesophagus, Bowel-Prostate, Oesophagus-Ovarian
BAP1	Breast-Prostate
BRCA1	Breast-Bowel, Breast-Lung, Breast-Oesophagus, Breast-Ovarian, Breast-Pancreas, Breast-Prostate, Pancreas-Ovarian, Prostate-Ovarian, Bowel-Ovarian, Endometrial-Ovarian, Breast-Endometrial
BRCA2	Breast-Lung, Breast-Oesophagus, Breast-Ovarian, Breast-Pancreas, Breast-Prostate, Pancreas-Ovarian, Prostate-Ovarian, Prostate-Pancreas, Bowel-Ovarian, Endometrial-Ovarian, Lung-Prostate, Prostate-Oesophagus, Bowel-Prostate
CHEK2	Breast-Bowel, Breast-Oesophagus, Breast-Ovarian, Breast-Pancreas, Breast-Prostate, Prostate-Ovarian, Prostate-Pancreas, Lung-Prostate, Prostate-Oesophagus, Bowel-Prostate
MAP3K1	Breast-Lung, Breast-Oesophagus, Breast-Ovarian, Breast-Pancreas, Breast-Prostate
MLH1	Bowel-Endometrial, Breast-Bowel, Bowel-Lung, Bowel-Ovarian, Bowel-Pancreas, Bowel-Oesophagus, Bowel-Prostate
MSH2	Bowel-Endometrial, Bowel-Lung, Bowel-Ovarian, Bowel-Pancreas, Bowel-Oesophagus, Bowel-Prostate
MSH6	Bowel-Endometrial, Bowel-Lung, Bowel-Ovarian, Bowel-Pancreas, Endometrial-Ovarian, Bowel-Oesophagus, Bowel-Prostate, Lung-Endometrial, Oesophagus-Endometrial, Pancreas-Endometrial, Prostate-Endometrial
PALB2	Breast-Lung, Breast-Oesophagus, Breast-Ovarian, Breast-Pancreas, Breast-Prostate

Table S19 | Posterior probabilities for the joint cancer modelling of breast and prostate cancer. The table includes the posterior probability genes are associated with just breast cancer, just prostate cancer, or both cancers. Results shown are for genes with any posterior probability>0.1 and are sorted by descending posterior probability.

Gene	P(C1 n C2')	P(C1' n C2)	P(C1 n C2)
BRCA2	0.000	0.000	1.000
BRCA1	0.000	0.000	1.000
PALB2	0.000	0.000	1.000
ATM	0.000	0.000	1.000
CHEK2	0.000	0.000	1.000
MAP3K1	0.000	0.000	0.972
BAP1	0.000	0.000	0.824
KLK4	0.000	0.000	0.320
PPP5C	0.000	0.000	0.293
OSGIN1	0.000	0.000	0.204
PNLDC1	0.000	0.000	0.156
GEMIN2	0.000	0.000	0.154
CYBC1	0.000	0.000	0.124
DEGS1	0.000	0.000	0.113
SEC62	0.000	0.000	0.107
SNX2	0.000	0.000	0.103

Table S20 | Posterior probabilities for the joint cancer modelling of breast and ovarian cancer. The table includes the posterior probability genes are associated with just breast cancer, just ovarian cancer, or both cancers. Results shown are for genes with any posterior probability>0.1 and are sorted by descending posterior probability.

Gene	P(C1 n C2')	P(C1' n C2)	P(C1 n C2)
BRCA2	0.000	0.000	1.000
BRCA1	0.000	0.000	1.000
PALB2	0.000	0.000	1.000
CHEK2	0.000	0.000	1.000
ATM	0.000	0.000	1.000
MAP3K1	0.000	0.000	0.979
BAP1	0.000	0.000	0.416
NHEJ1	0.000	0.000	0.300
RAD51D	0.000	0.000	0.282
BRIP1	0.000	0.000	0.259
SLC35E4	0.000	0.000	0.241
KLK4	0.000	0.000	0.206
VWA2	0.000	0.000	0.200
BARD1	0.000	0.000	0.139
IVD	0.000	0.000	0.139
RNF112	0.000	0.000	0.133
ZNHIT1	0.000	0.000	0.132
COL12A1	0.000	0.000	0.130
PCDHGB3	0.000	0.000	0.122
TGM7	0.000	0.000	0.117
CYBC1	0.000	0.000	0.113
PSRC1	0.000	0.000	0.110
TRMT10B	0.000	0.000	0.105
PLEKHG4	0.000	0.000	0.104

Table S21 | Posterior probabilities for the joint cancer modelling of bowel and endometrial cancer. The table includes the posterior probability genes are associated with just bowel cancer, just endometrial cancer, or both cancers. Results shown are for genes with any posterior probability>0.1 and are sorted by descending posterior probability.

Gene	P(C1 n C2')	P(C1' n C2)	P(C1 n C2)
MSH6	1.92E-42	0	1.000
MLH1	0.019	0.000	0.981
MSH2	0.020	0.000	0.980
APC	0.481	0.000	0.519
GAPDH	0.475	0.000	0.182
FLCN	0.291	0.000	0.106
RDX	0.012	0.000	0.128
NPNT	0.122	0.000	0.051
ACRV1	0.001	0.000	0.116
MPPE1	0.113	0.000	0.029

Table S22 | Posterior probabilities for the joint cancer modelling of breast and pancreatic cancer. The table includes the posterior probability genes are associated with just breast cancer, just pancreatic cancer, or both cancers. Results shown are for genes with any posterior probability>0.1 and are sorted by descending posterior probability.

Gene	P(C1 n C2')	P(C1' n C2)	P(C1 n C2)
BRCA2	0.000	0.000	1.000
BRCA1	0.000	0.000	1.000
PALB2	0.000	0.000	1.000
ATM	0.000	0.000	1.000
MAP3K1	0.000	0.000	0.980
BAP1	0.000	0.000	0.393
RNF112	0.000	0.000	0.130
PCDHGB3	0.000	0.000	0.146
KLK4	0.000	0.000	0.192
COL12A1	0.000	0.000	0.131
CYBC1	0.000	0.000	0.122
CHEK2	0.000	0.000	1.000
RCN2	0.000	0.000	0.144

Table S23 | Posterior probabilities for the joint cancer modelling of prostate and ovarian cancer. The table includes the posterior probability genes are associated with just prostate cancer, just ovarian cancer, or both cancers. Results shown are for genes with any posterior probability>0.1 and are sorted by descending posterior probability.

Gene	P(C1 n C2')	P(C1' n C2)	P(C1 n C2)
BRCA2	0	1.47E-12	1.000
CHEK2	0	3.49E-08	1.000
ATM	0	5.50E-06	1.000
BRCA1	0	0.194	0.806
IVD	0	0.119	0.468
FKBP6	0	0.021	0.421
ANO2	0	0.031	0.372
PPP5C	0	0.002	0.297
BET1	0	0.003	0.292
SLC35E4	0	0.102	0.263
PLEKHG4	0	0.076	0.261
ESYT1	0	0.032	0.260
REXO5	0	0.089	0.254
NEK11	0	0.029	0.250
DPH1	0	0.008	0.238
SORD	0	0.008	0.223
OIP5	0	0.018	0.214
PNLDC1	0	0.002	0.203
CHID1	0	0.003	0.201
PNLIP	0	0.032	0.198
BRIP1	0	0.038	0.195
INVS	0	0.001	0.188
PALB2	0	0.009	0.175
WDFY4	0	0.010	0.170
JAML	0	0.034	0.169
MSH6	0	0.025	0.162
MICB	0	0.002	0.161
R3HCC1L	0	0.026	0.161
TMC2	0	0.004	0.161
ADH1B	0	0.033	0.151
PPEF2	0	0.002	0.147
WDR59	0	0.003	0.147
CEACAM20	0	0.025	0.146
ELP4	0	0.050	0.146
RELT	0	0.022	0.146
TRMT44	0	0.002	0.146
C9orf50	0	0.004	0.145
UBOX5	0	0.022	0.144
ERCC3	0	0.006	0.144
MYH7	0	0.002	0.143
SSNA1	0	0.004	0.139
DOCK2	0	0.019	0.136

PABPN1	0	0.004	0.133
ITGB3	0	0.006	0.132
TRIT1	0	0.015	0.130
GEMIN2	0	0.003	0.126
TMPRSS6	0	0.007	0.118
POLE	0	0.020	0.113
FSIP2	0	0.002	0.113
BEND5	0	0.003	0.112
NHEJ1	0	0.030	0.111
IFNL1	0	0.007	0.111
IFT57	0	0.009	0.110
PIGV	0	0.006	0.109
BRD1	0	0.023	0.108
LMF2	0	0.005	0.107
MARK3	0	0.025	0.107
SNX2	0	0.005	0.106
SPG7	0	0.004	0.105
MXD3	0	0.007	0.105
ZNHIT1	0	0.027	0.104
MFSD8	0	0.003	0.103
PITPNM1	0	0.019	0.103
RAD51D	0	0.028	0.101
FAM161A	0	0.011	0.100
ZRANB3	0	0.028	0.100

Table S24 | Posterior probabilities for the joint cancer modelling of prostate and pancreatic cancer. The table includes the posterior probability genes are associated with just prostate cancer, just pancreatic cancer, or both cancers. Results shown are for genes with any posterior probability>0.1 and are sorted by descending posterior probability.

Gene	**P(C1 n C2')**	**P(C1' n C2)**	**P(C1 n C2)**
BRCA2	0.000	0.000	1.000
ATM	0.000	0.000	1.000
CHEK2	0.000	0.000	1.000
SEC14L3	0.000	0.000	0.745
INVS	0.000	0.000	0.481
GEMIN2	0.000	0.000	0.232
PPP5C	0.000	0.000	0.210
BET1	0.000	0.000	0.183
C9orf50	0.000	0.000	0.148
PALB2	0.000	0.000	0.147
LYST	0.000	0.000	0.145
PNLDC1	0.000	0.000	0.128
SMC2	0.000	0.000	0.114
PACSIN3	0.000	0.000	0.112
MYH7	0.000	0.000	0.110
RCN2	0.000	0.000	0.110
SAG	0.000	0.000	0.105

Table S25 | Posterior probabilities for the joint cancer modelling of breast and lung cancer. The table includes the posterior probability genes are associated with just breast cancer, just lung cancer, or both cancers. Results shown are for genes with any posterior probability>0.1 and are sorted by descending posterior probability.

Gene	**P(C1 n C2')**	**P(C1' n C2)**	**P(C1 n C2)**
ATM	0.000979	2.74E-16	0.999
BRCA2	0.00443	4.25E-150	0.996
BRCA1	0.0760	7.27E-77	0.924
MAP3K1	0.0734	5.77E-06	0.906
PALB2	0.145	1.58E-45	0.855
CHEK2	0.290	9.24E-43	0.710
BAP1	0.0160	0.000272	0.270
PCDHGB3	0.0141	0.000199	0.149
LZTR1	0.00194	0.00108	0.104

Table S26| Posterior probabilities for the joint cancer modelling of breast and bowel cancer. The table includes the posterior probability genes are associated with just breast cancer, just bowel cancer, or both cancers. Results shown are for genes with any posterior probability>0.1 and are sorted by descending posterior probability.

Gene	P(C1 n C2')	P(C1' n C2)	P(C1 n C2)
ATM	0.0158	2.62E-16	0.984
BRCA1	0.0218	7.39E-77	0.978
MLH1	4.84E-17	0.149	0.851
APC	3.67E-11	0.183	0.817
CHEK2	0.198	1.01E-42	0.802
BARD1	0.00672	0.00211	0.791
BRCA2	0.310	2.84E-150	0.690
MSH6	1.74E-31	0.346	0.654
MSH2	3.24E-20	0.346	0.654
MAP3K1	0.453	3.18E-06	0.516
PALB2	0.491	9.12E-46	0.509
GAPDH	0.000227	0.0825	0.491
BAP1	0.0610	0.000268	0.274
NPNT	0.000825	0.0162	0.188
CTTNBP2NL	0.00265	0.00390	0.134

Table S28 | Posterior probabilities for the joint cancer modelling of breast and oesophagus cancer. The table includes the posterior probability genes are associated with just breast cancer, just oesophagus cancer, or both cancers. Results shown are for genes with any posterior probability>0.1 and are sorted by descending posterior probability.

Gene	P(C1 n C2')	P(C1' n C2)	P(C1 n C2)
BRCA2	0.000	1.14E-148	1.000
BRCA1	0.000	2.21E-75	1.000
PALB2	0.000	4.74E-44	1.000
CHEK2	0.000	3.15E-41	1.000
ATM	0.000	6.57E-15	1.000
MAP3K1	0.000	0.000	0.982
NLRP12	0.000	0.399	0.043
BAP1	0.000	0.007	0.268
RNF112	0.000	0.010	0.199
PCDHGB3	0.000	0.005	0.171
KLK4	0.000	0.014	0.168
COL12A1	0.000	0.007	0.156
KNL1	0.000	0.146	0.012
BARD1	0.000	0.009	0.146
ZGRF1	0.000	0.130	0.003
CYBC1	0.000	0.009	0.126

Table S29 | Posterior probabilities for the joint cancer modelling of lung and pancreatic cancer. The table includes the posterior probability genes are associated with just lung cancer, just pancreatic cancer, or both cancers. Results shown are for genes with any posterior probability>0.1 and are sorted by descending posterior probability.

Gene	P(C1 n C2')	P(C1' n C2)	P(C1 n C2)
ATM	0.000	0.000	1.000
BRCA2	0.000	0.000	0.438
LTV1	0.000	0.000	0.163
SEC14L3	0.000	0.000	0.161
FANCM	0.000	0.000	0.106

Supplementary Figures

Pancreatic Cancer

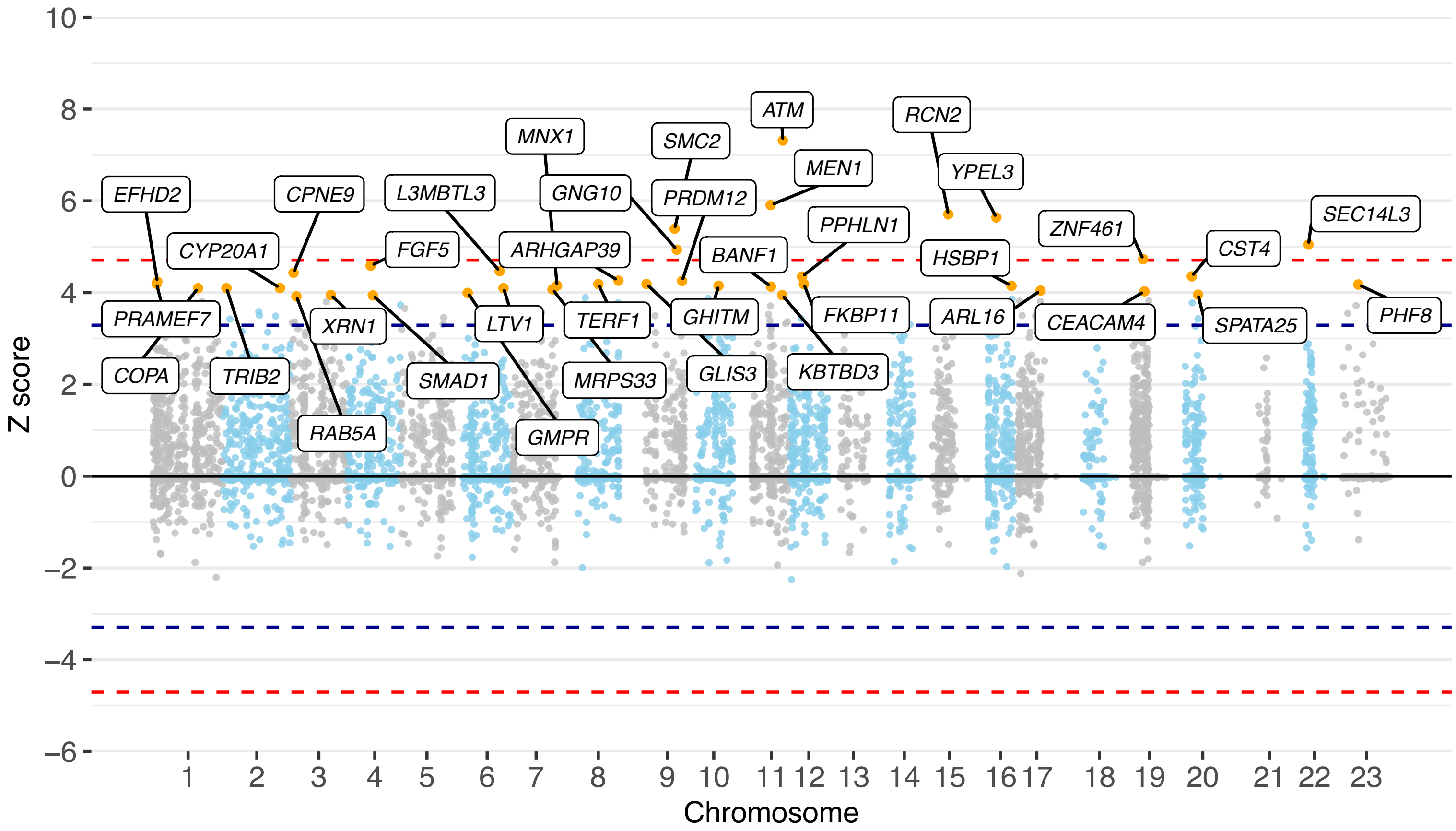

Figure S1 | Manhattan plot of z scores from assessing the association between protein-truncating variant carriers within genes and pancreatic cancer risk, using model 2. The x axis is the chromosomal position, and the y axis is the z score from testing H0: $\beta = \ln(OR) = 0$ (two-tailed) by LRT to the null model. The blue lines correspond to $z = \pm 3.29$, $P = 0.001$, the red lines correspond to $z = \pm 4.71$, $P = 2.5 \times 10{-}6$. All labelled genes are those with $P < 0.001$. All P-values are unadjusted for multiple testing.

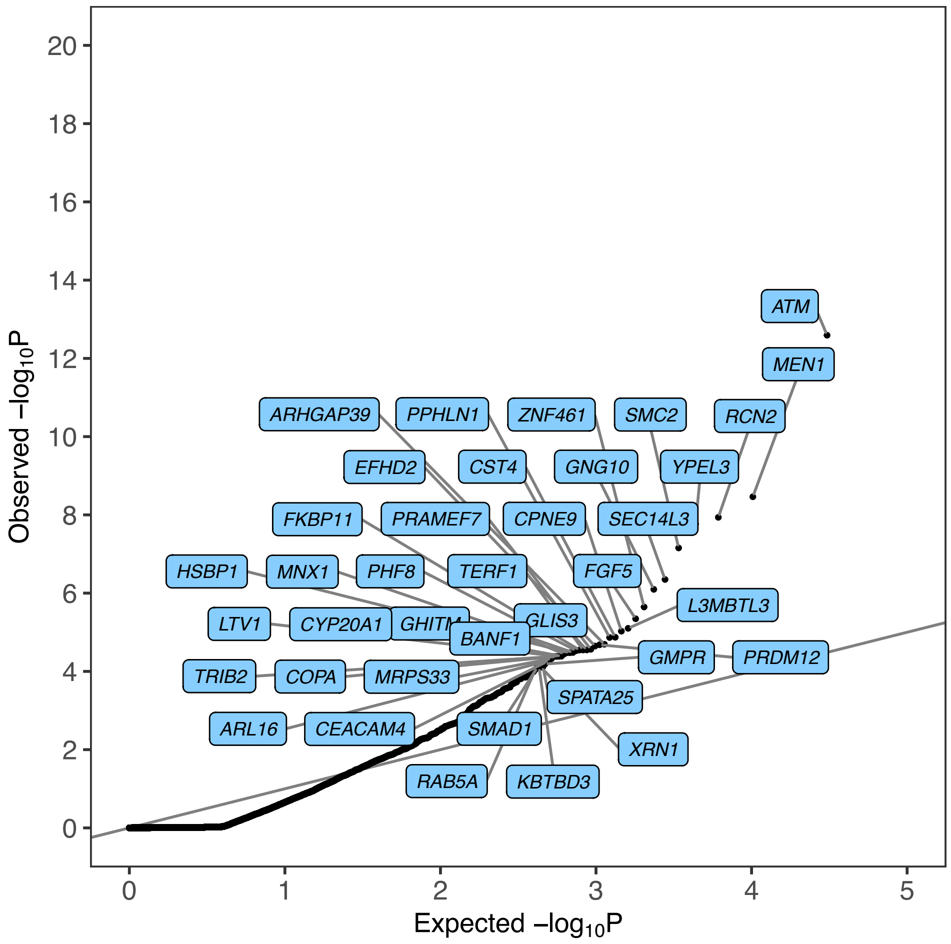

Figure S2 | Quantile–quantile plot of P values from assessing the association between protein-truncating variant carriers and pancreatic cancer risk. P-values are from testing H0: $\beta = \ln(OR) = 0$ by LRT to the null model (two-tailed). The x-axis is the expected log10 P values from the null hypothesis, the y-axis is the observed log10 P value. Highlighted genes have $P < 0.0001$. Highlighted genes in blue are associated with an increased risk of pancreatic cancer and highlighted genes in cream are associated with decreased risk of pancreatic cancer. All P-values are unadjusted for multiple testing.

Endometrial Cancer

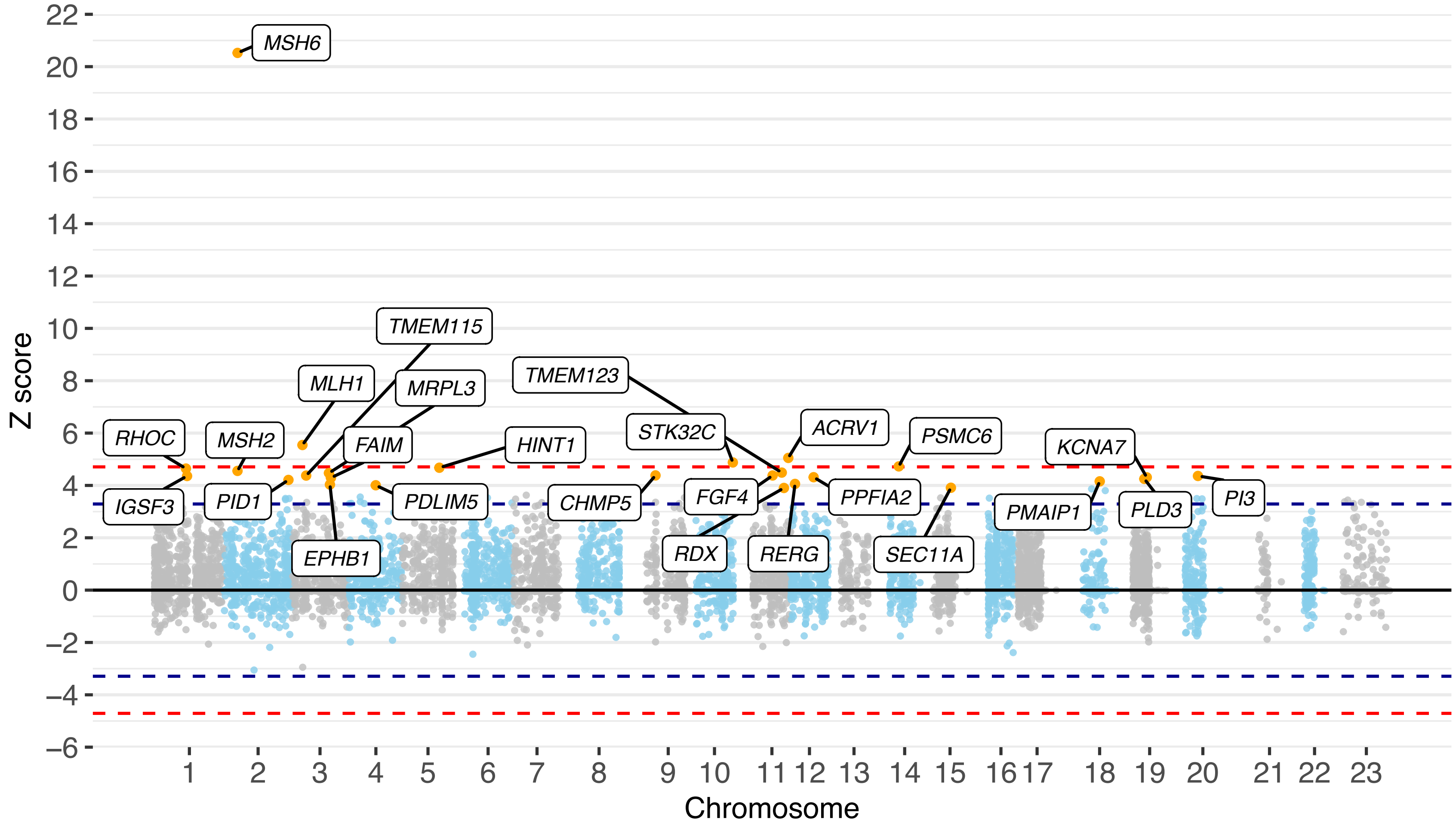

Figure S3 | Manhattan plot of z scores from assessing the association between protein-truncating variant carriers within genes and endometrial cancer risk, using model 1. The x axis is the chromosomal position, and the y axis is the z score from testing H0: β = ln(OR) = 0 (two-tailed) by LRT to the null model. The blue lines correspond to z = ±3.29, P = 0.001, the red lines correspond to z = ±4.71, P = 2.5 × 10−6. All labelled genes are those with P < 0.001. All P-values are unadjusted for multiple testing.

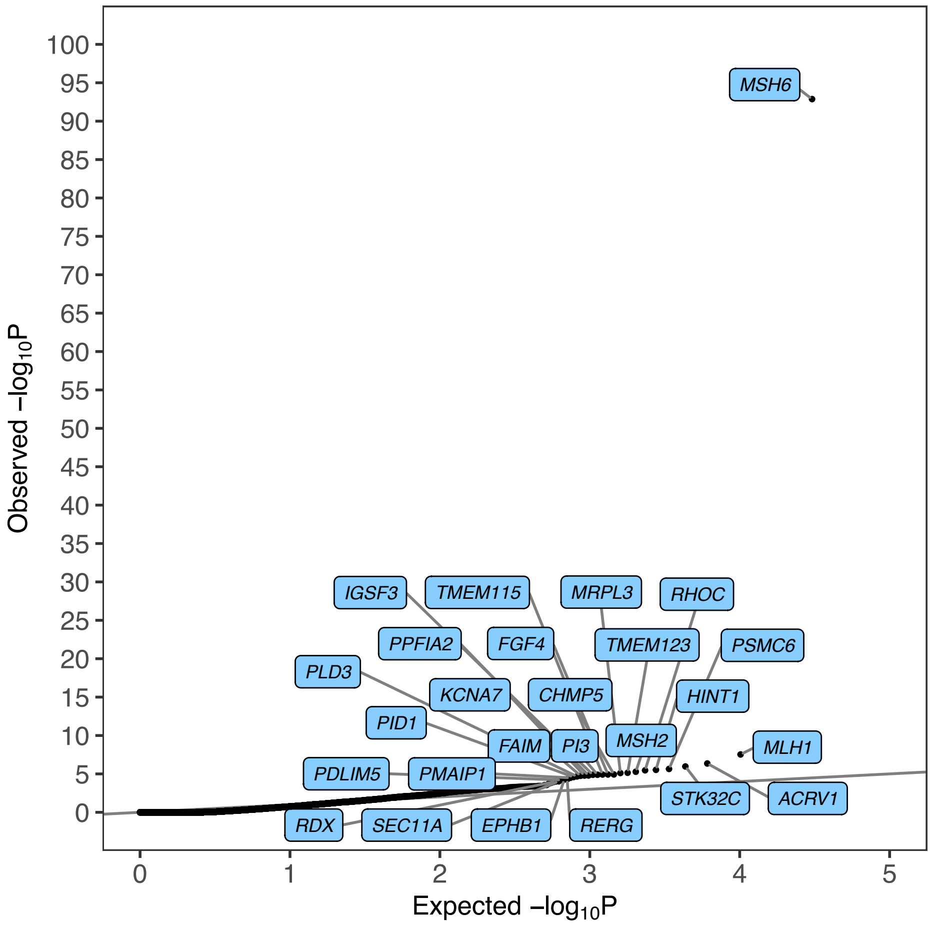

Figure S4 | Quantile–quantile plot of P values from assessing the association between protein-truncating variant carriers and endometrial cancer risk. P-values are from testing H0: $\beta = \ln(OR) = 0$ by LRT to the null model (two-tailed). The x-axis is the expected log10 P values from the null hypothesis, the y-axis is the observed log10 P value. Highlighted genes have $P < 0.0001$. Highlighted genes in blue are associated with an increased risk of endometrial cancer and highlighted genes in cream are associated with decreased risk of endometrial cancer. All P-values are unadjusted for multiple testing.

Ovarian Cancer

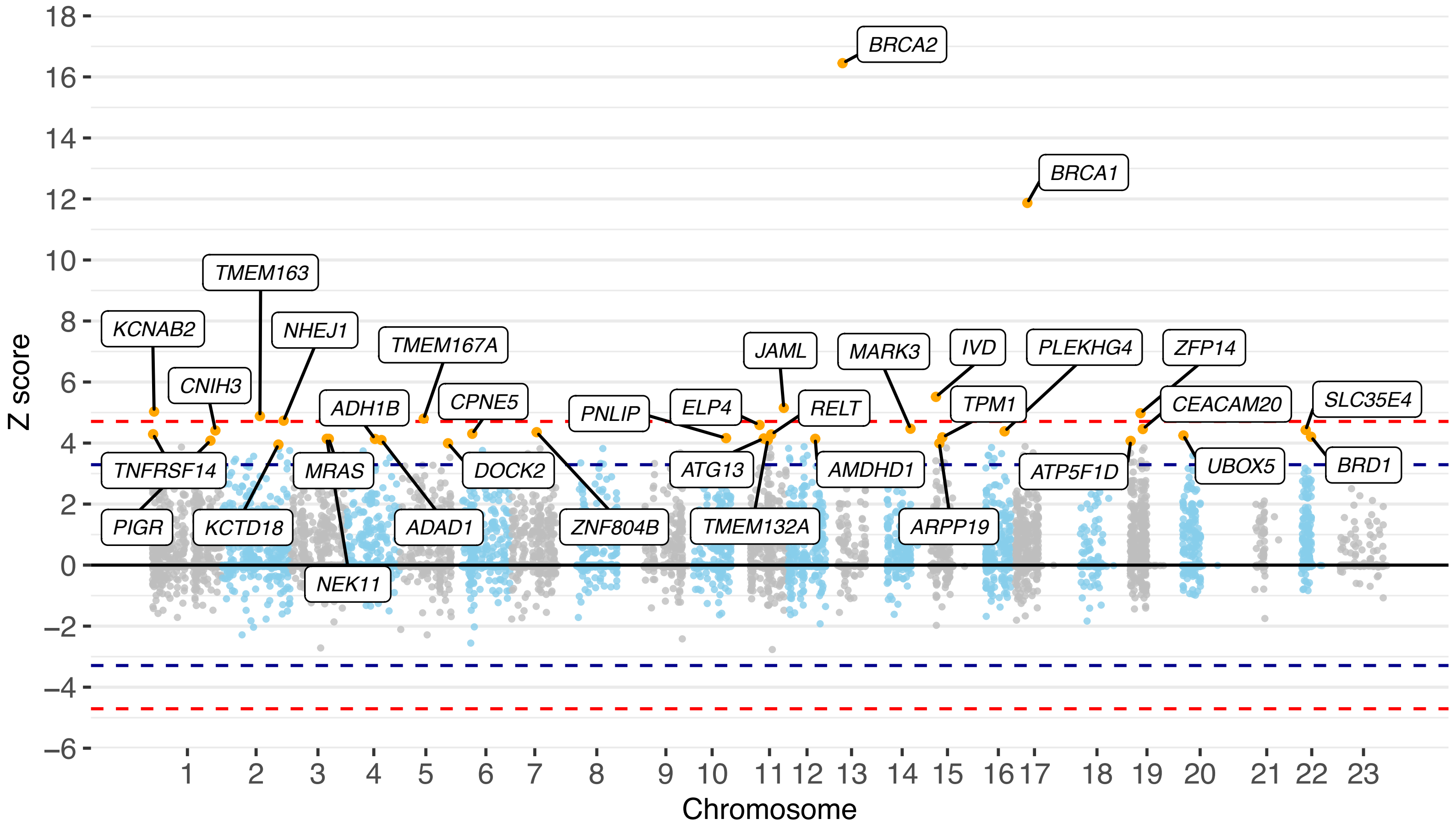

Figure S5 | Manhattan plot of z scores from assessing the association between protein-truncating variant carriers within genes and ovarian cancer risk, using model 1. The x axis is the chromosomal position, and the y axis is the z score from testing H0: $\beta = \ln(OR) = 0$ (two-tailed) by LRT to the null model. The blue lines correspond to $z = \pm 3.29$, $P = 0.001$, the red lines correspond to $z = \pm 4.71$, $P = 2.5 \times 10{-}6$. All labelled genes are those with $P < 0.001$. All P-values are unadjusted for multiple testing.

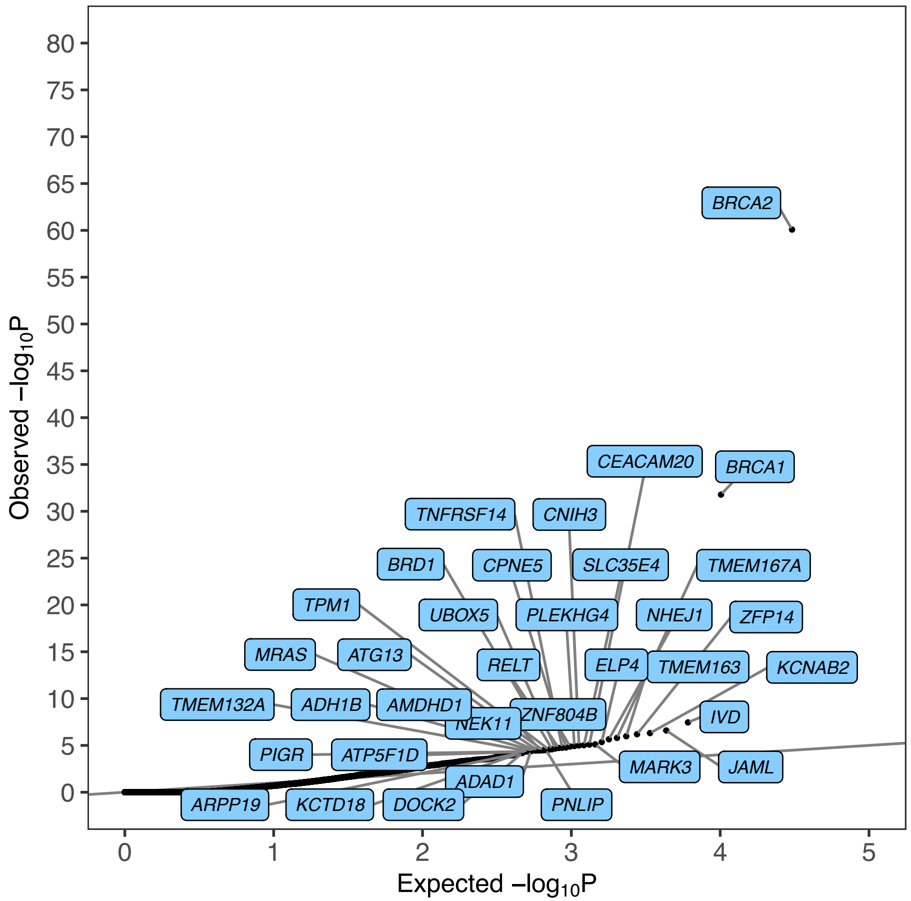

Figure S6 | Quantile–quantile plot of P values from assessing the association between protein-truncating variant carriers and ovarian cancer risk. P-values are from testing H0: $\beta = \ln(OR) = 0$ by LRT to the null model (two-tailed). The x-axis is the expected log10 P values from the null hypothesis, the y-axis is the observed log10 P value. Highlighted genes have $P < 0.0001$. Highlighted genes in blue are associated with an increased risk of ovarian cancer and highlighted genes in cream are associated with decreased risk of ovarian cancer. All P-values are unadjusted for multiple testing

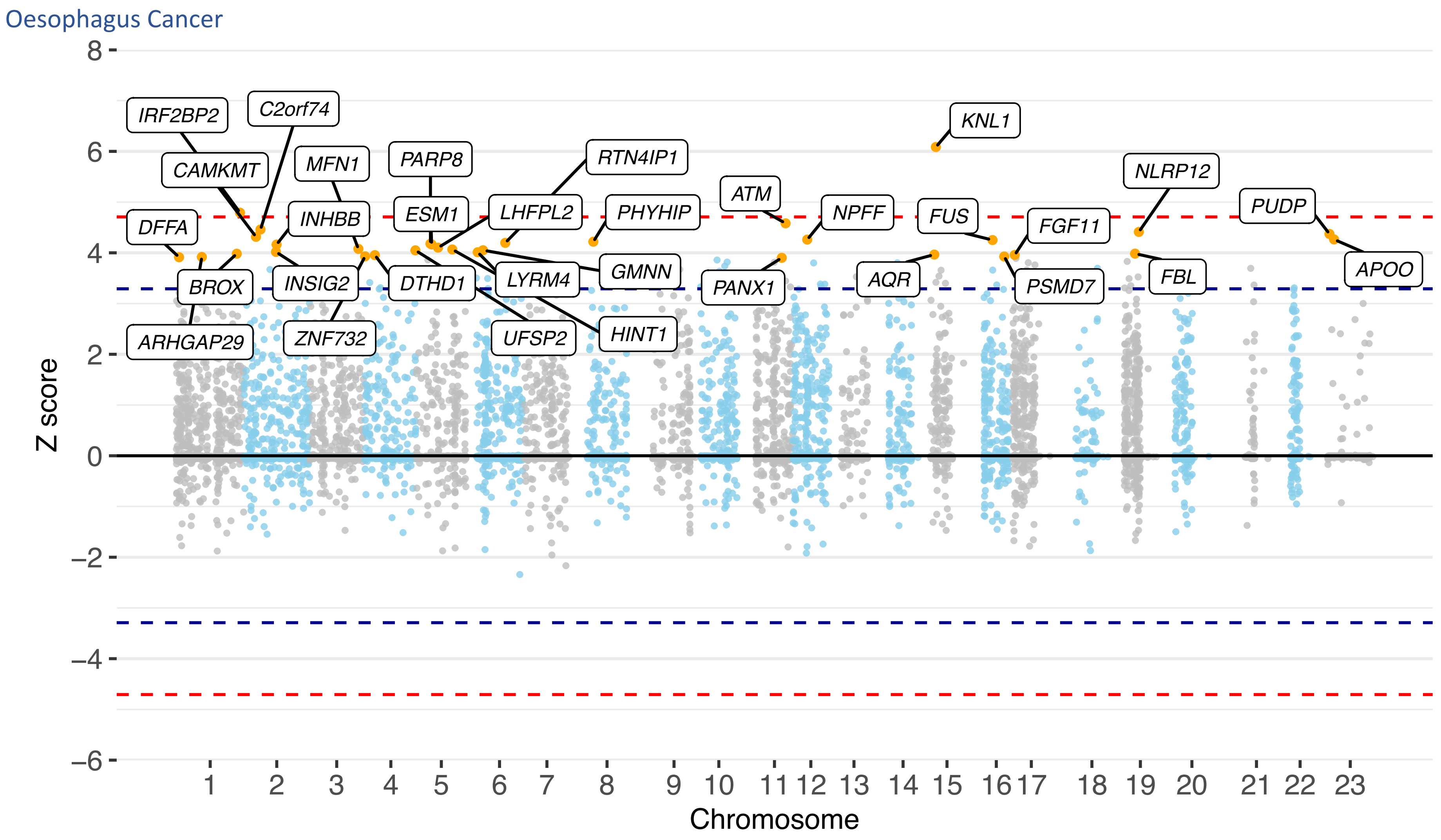

Figure S7 | Manhattan plot of z scores from assessing the association between protein-truncating variant carriers within genes and oesophagus cancer risk, using model 2. The x axis is the chromosomal position, and the y axis is the z score from testing H0: β = ln(OR) = 0 (two-tailed) by LRT to the null model. The blue lines correspond to z = ±3.29, P = 0.001, the red lines correspond to z = ±4.71, P = 2.5 × 10−6. All labelled genes are those with P < 0.001. All P-values are unadjusted for multiple testing.

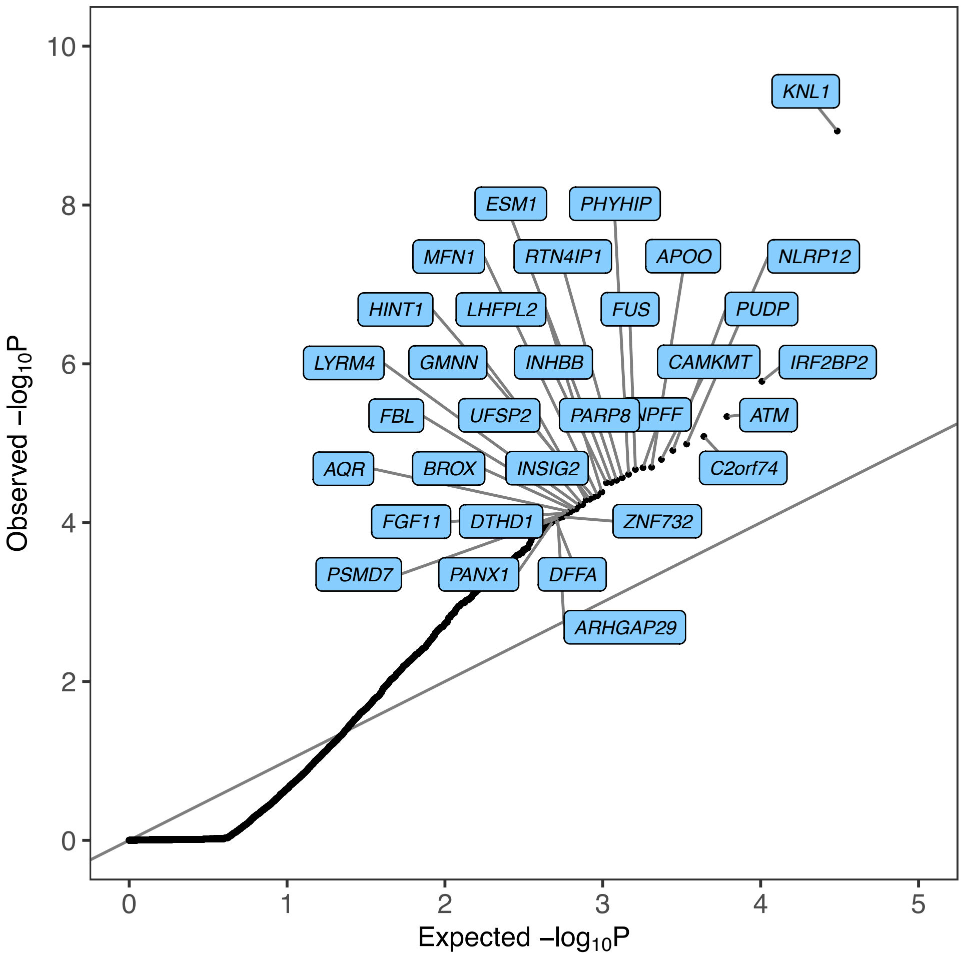

Figure S8 | Quantile–quantile plot of P values from assessing the association between protein-truncating variant carriers and oesophagus cancer risk. P-values are from testing H0: β = ln(OR) = 0 by LRT to the null model (two-tailed). The x-axis is the expected log10 P values from the null hypothesis, the y-axis is the observed log10 P value. Highlighted genes have P < 0.0001. Highlighted genes in blue are associated with an increased risk of oesophagus cancer and highlighted genes in cream are associated with decreased risk of oesophagus cancer. All P-values are unadjusted for multiple testing.

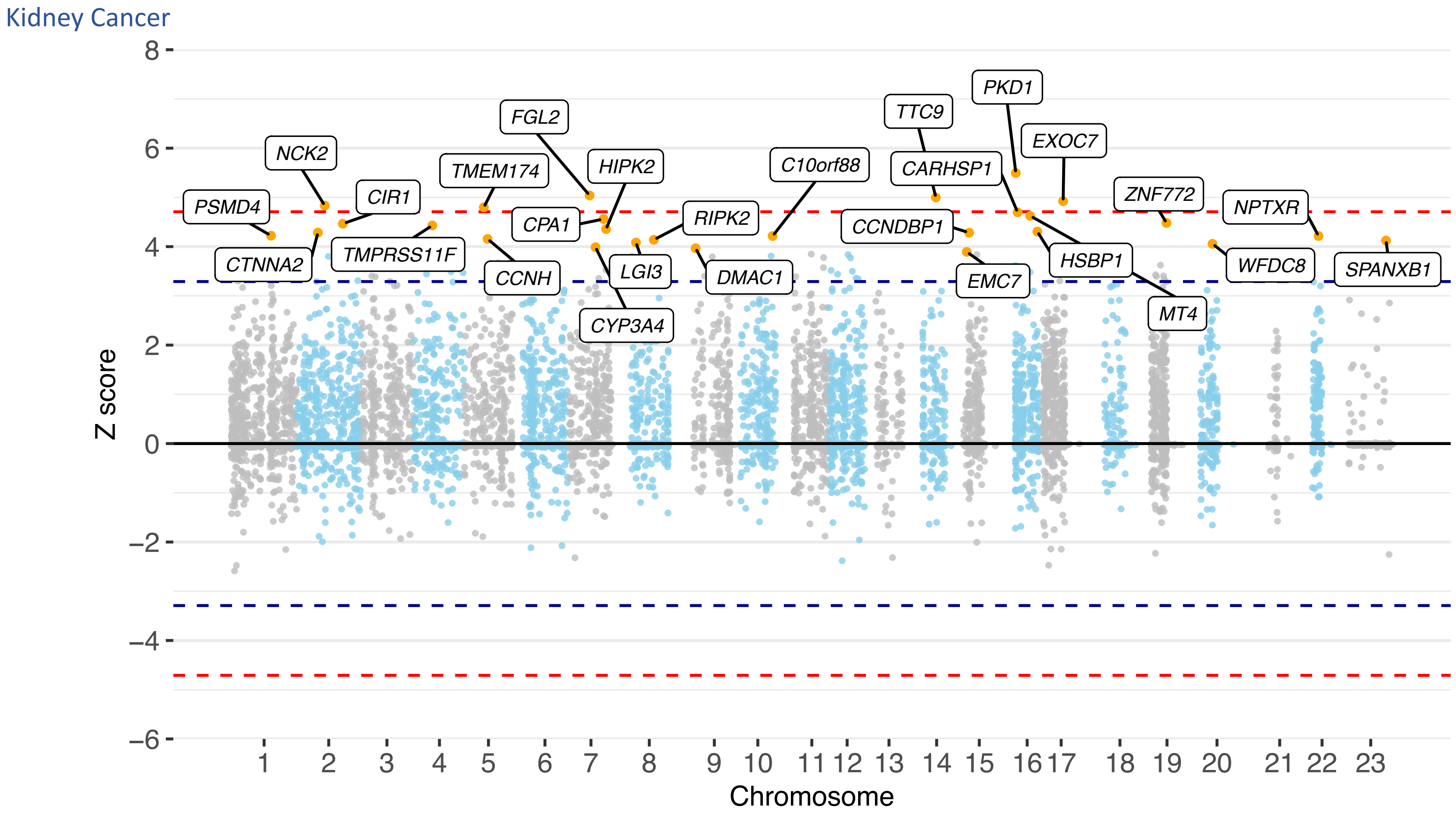

Figure S9 | Manhattan plot of z scores from assessing the association between protein-truncating variant carriers within genes and kidney cancer risk, using model 2. The x axis is the chromosomal position, and the y axis is the z score from testing H0: β = ln(OR) = 0 (two-tailed) by LRT to the null model. The blue lines correspond to z = ±3.29, P = 0.001, the red lines correspond to z = ±4.71, P = 2.5 × 10−6. All labelled genes are those with P < 0.001. All P-values are unadjusted for multiple testing.

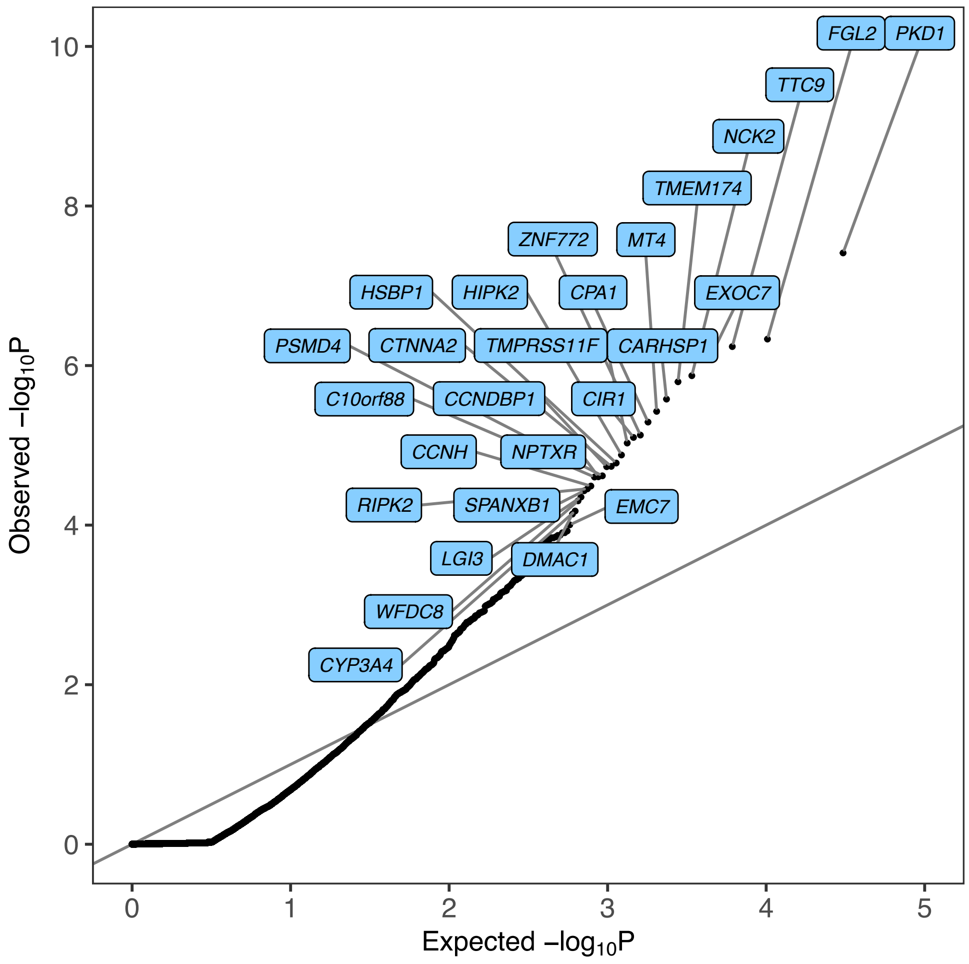

Figure S10 | Quantile–quantile plot of P values from assessing the association between protein-truncating variant carriers and kidney cancer risk. P-values are from testing H0: $\beta = \ln(OR) = 0$ by LRT to the null model (two-tailed). The x-axis is the expected log10 P values from the null hypothesis, the y-axis is the observed log10 P value. Highlighted genes have $P < 0.0001$. Highlighted genes in blue are associated with an increased risk of kidney cancer and highlighted genes in cream are associated with decreased risk of kidney cancer. All P-values are unadjusted for multiple testing.

Bladder Cancer

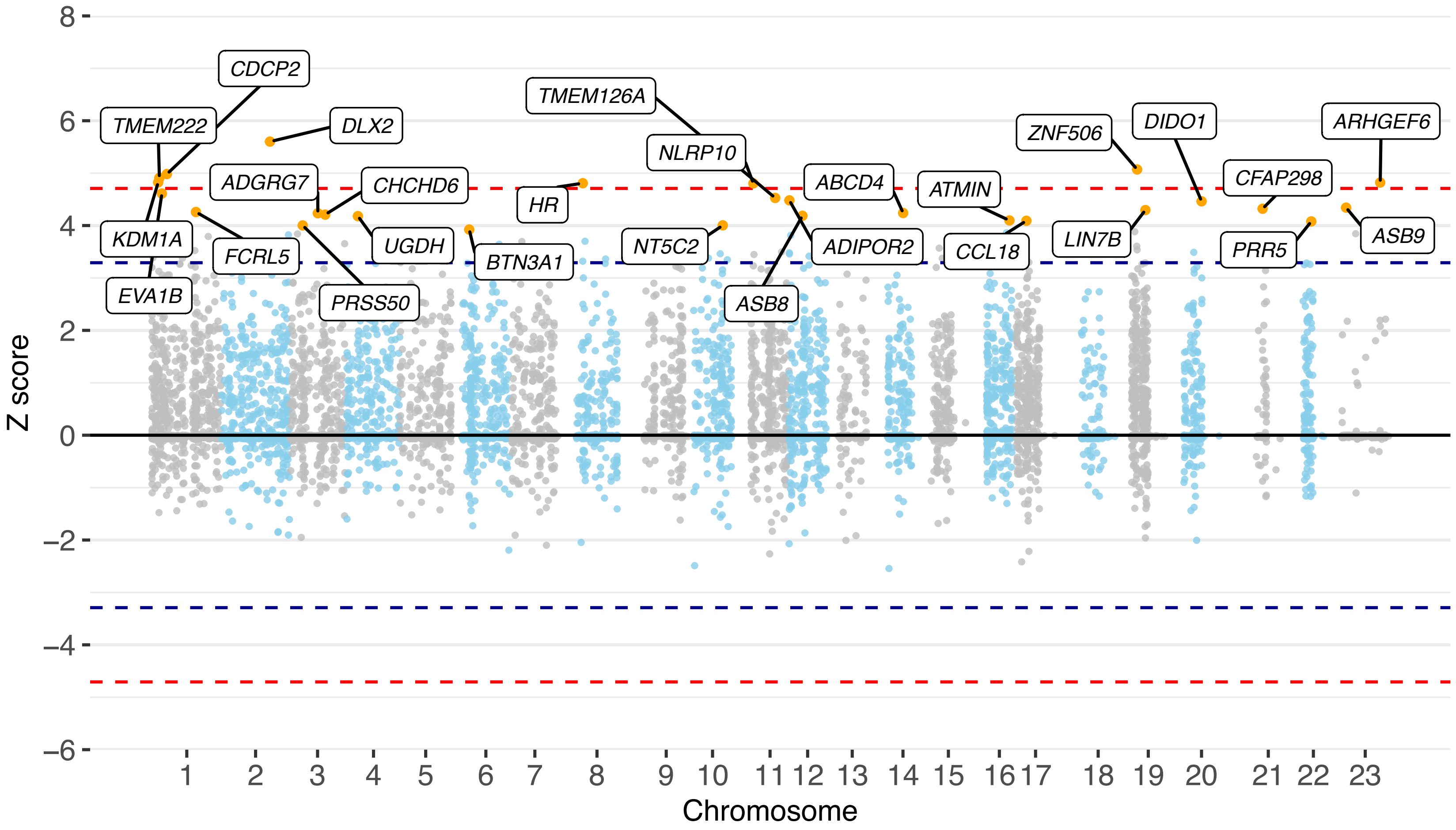

Figure S11 | Manhattan plot of z scores from assessing the association between protein-truncating variant carriers within genes and bladder cancer risk, using model 2. The x axis is the chromosomal position, and the y axis is the z score from testing H0: β = ln(OR) = 0 (two-tailed) by LRT to the null model. The blue lines correspond to z = ±3.29, P = 0.001, the red lines correspond to z = ±4.71, P = 2.5 × 10−6. All labelled genes are those with P < 0.001. All P-values are unadjusted for multiple testing.

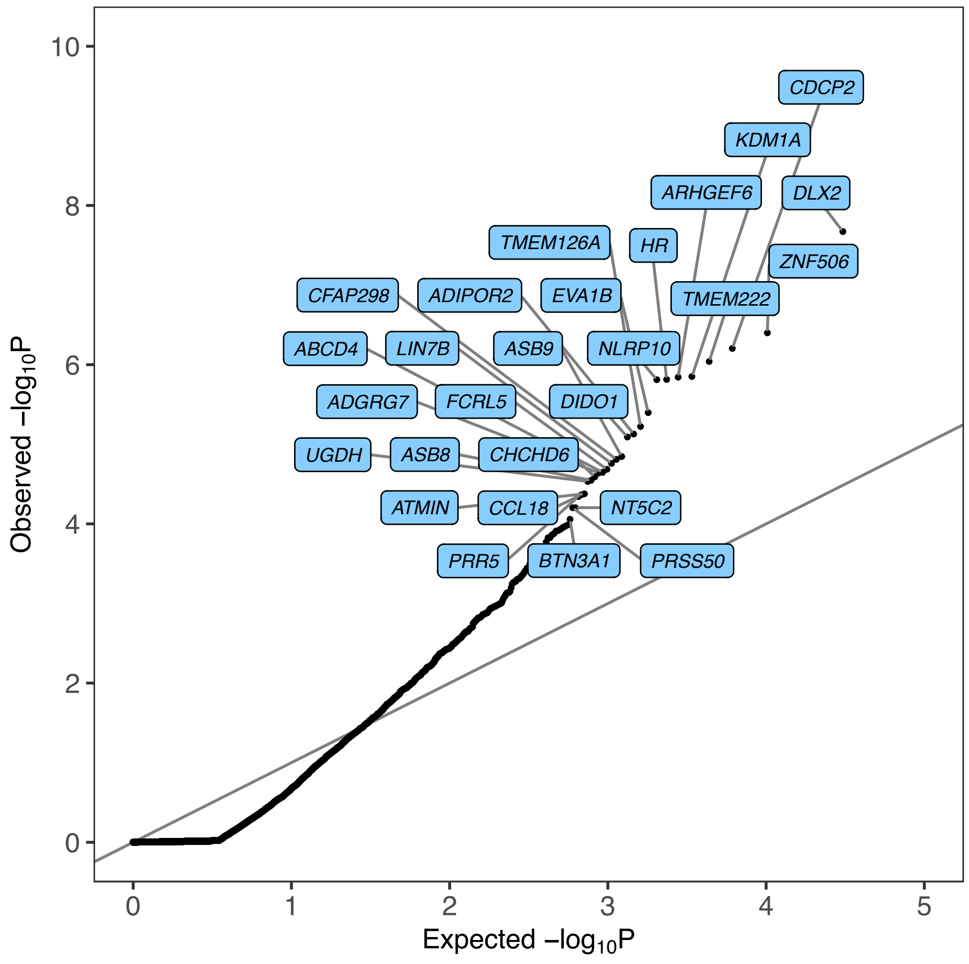

Figure S12 | Quantile–quantile plot of P values from assessing the association between protein-truncating variant carriers and kidney cancer risk. P-values are from testing H0: $\beta = \ln(OR) = 0$ by LRT to the null model (two-tailed). The x-axis is the expected log10 P values from the null hypothesis, the y-axis is the observed log10 P value. Highlighted genes have $P < 0.0001$. Highlighted genes in blue are associated with an increased risk of kidney cancer and highlighted genes in cream are associated with decreased risk of kidney cancer. All P-values are unadjusted for multiple testing.

Malignant Melanoma

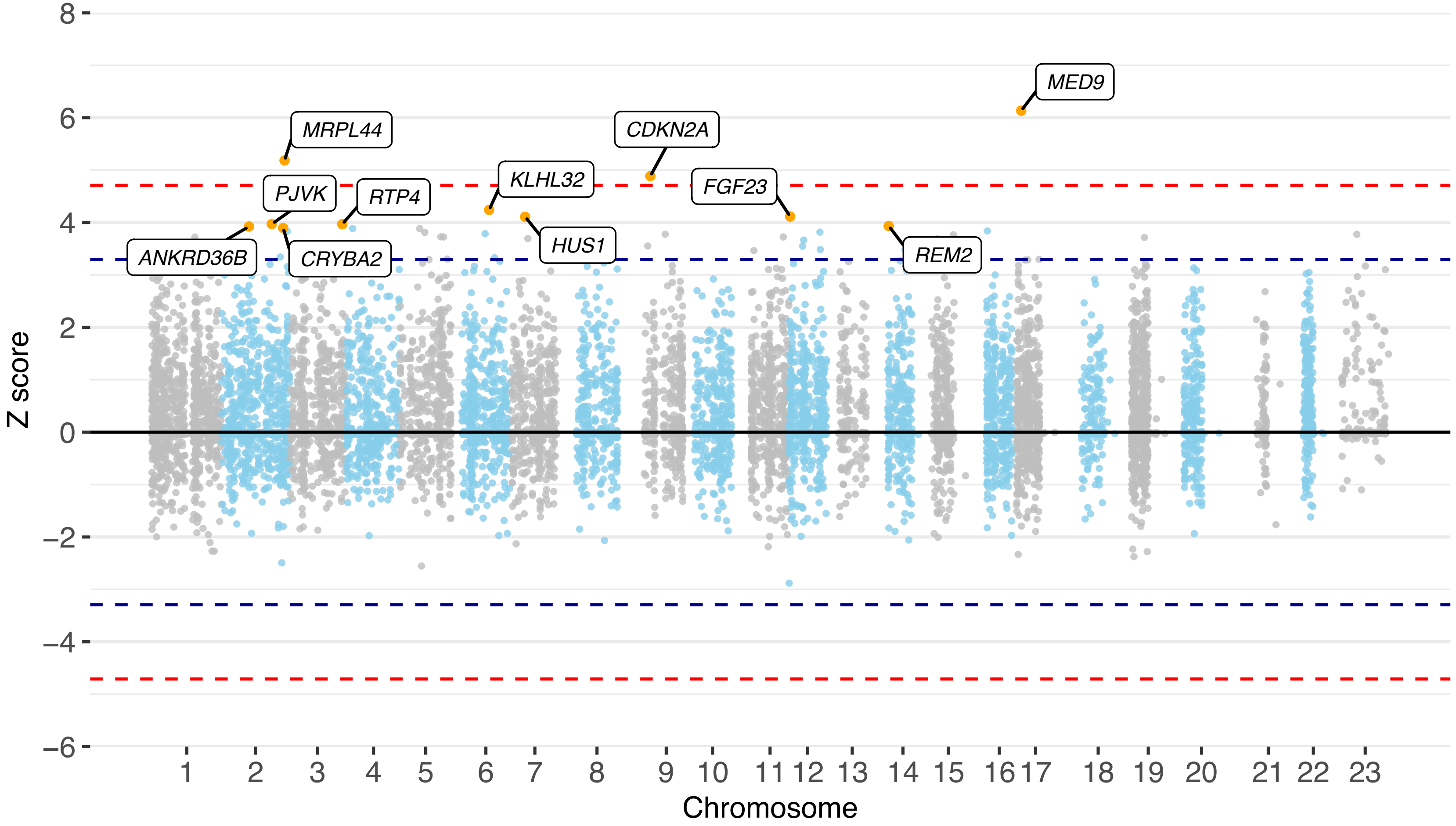

Figure S13 | Manhattan plot of z scores from assessing the association between protein-truncating variant carriers within genes and malignant melanoma risk, using model 2. The x axis is the chromosomal position, and the y axis is the z score from testing H0: β = ln(OR) = 0 (two-tailed) by LRT to the null model. The blue lines correspond to z = ±3.29, P = 0.001, the red lines correspond to z = ±4.71, P = 2.5 × 10−6. All labelled genes are those with P < 0.001. All P-values are unadjusted for multiple testing.

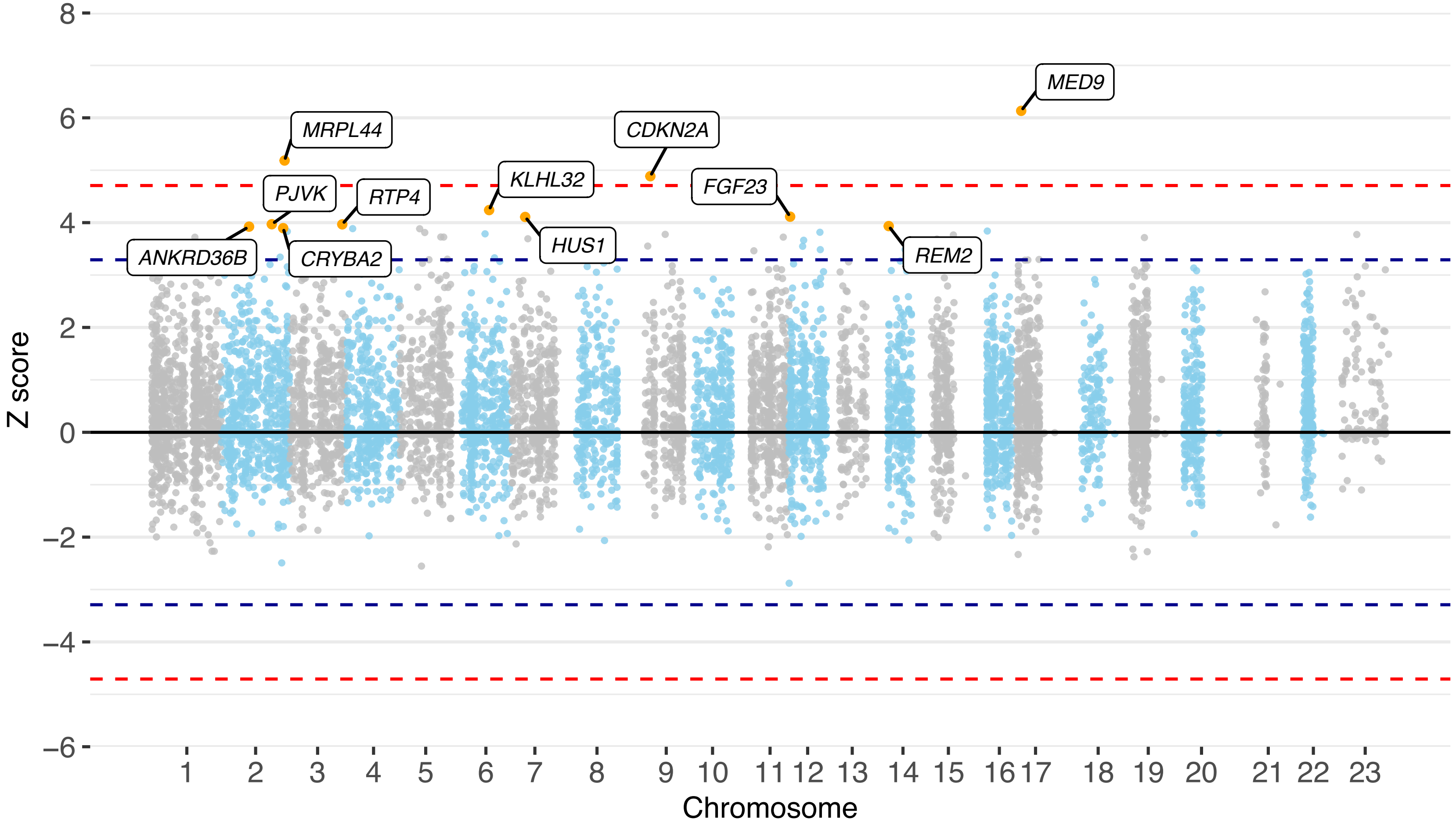

Figure S14 | Manhattan plot of z-scores from assessing the association between protein-truncating variant carriers within genes and malignant melanoma risk, using model 2. The x-axis is the chromosomal position, and the y-axis is the z-score from testing H0: $\beta = \ln(OR) = 0$ (two-tailed) by LRT to the null model. The blue lines correspond to $z = \pm 3.29$, $P = 0.001$, the red lines correspond to $z = \pm 4.71$, $P = 2.5 \times 10{-}6$. All labelled genes are those with $P < 0.001$. All P-values are unadjusted for multiple testing.

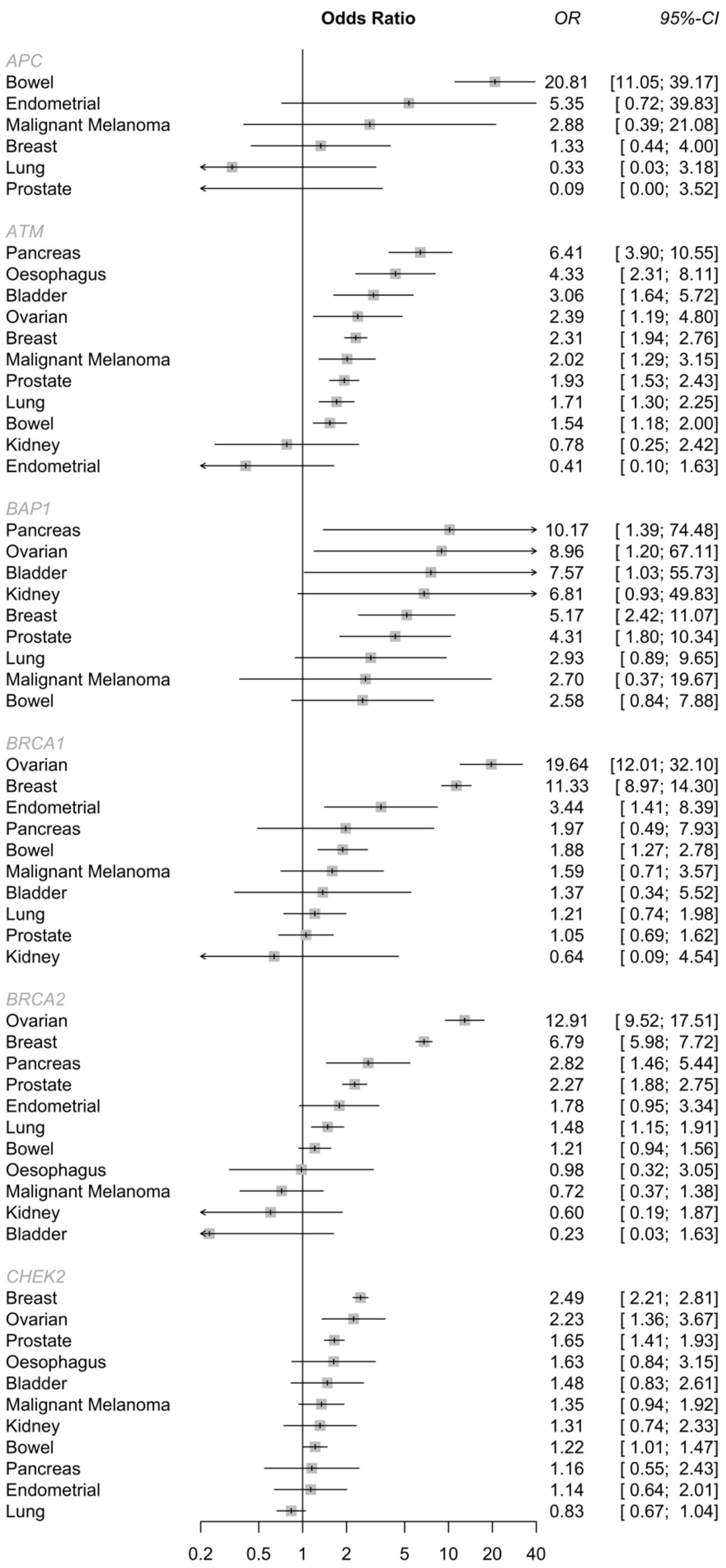

Figure S15a | Forest plot of PTV burden results across each cancer for the 11 genes with posterior probability > 0.8 in any analysis, and *NHEJ1*. For each gene, cancers with 0 case carriers were removed.

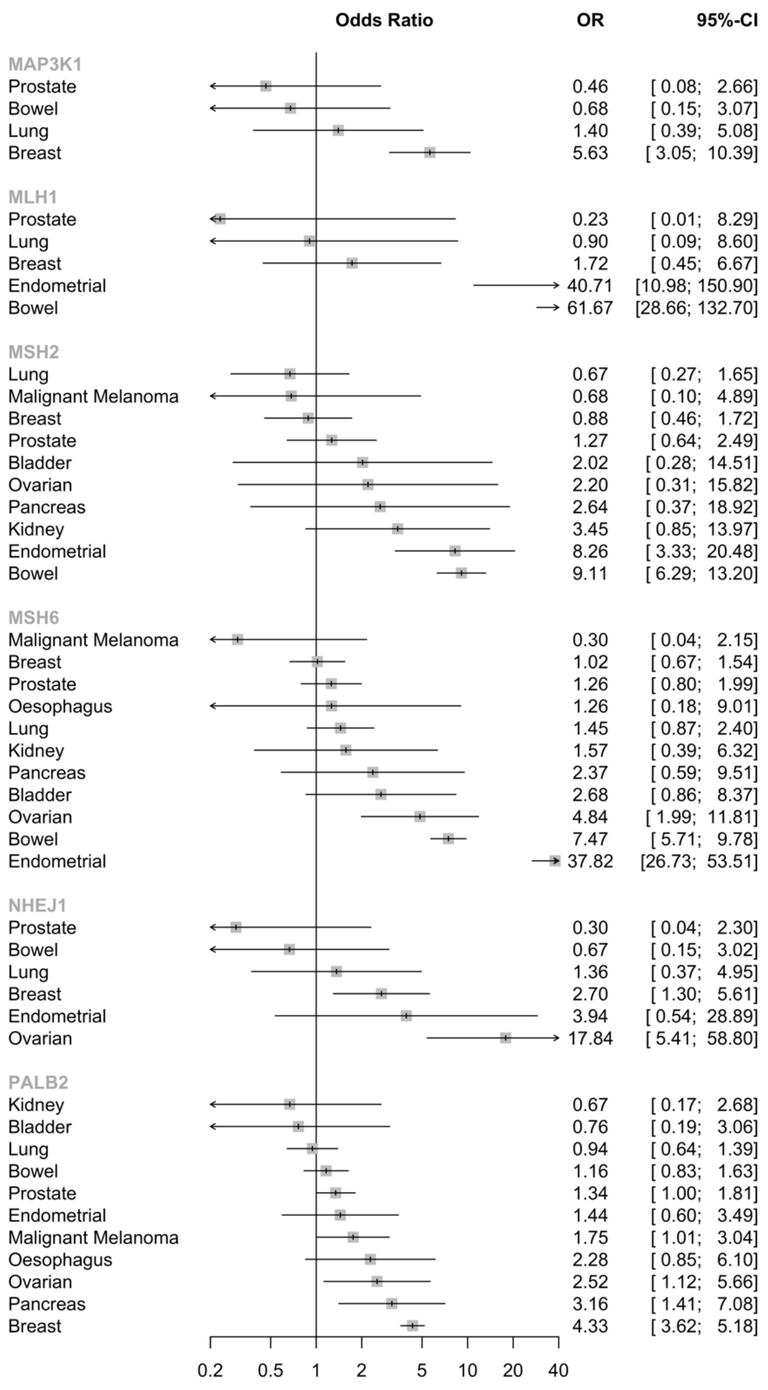

Figure S15b | Forest plot of PTV burden results across each cancer for the 11 genes with posterior probability > 0.8 in any analysis, and *NHEJ1*. For each gene, cancers with 0 case carriers were removed.
