# Supplementary material for: The contribution of coding variants to the heritability of multiple cancer types using UK Biobank whole-exome sequencing data
Source: Am J Hum Genet. 2025 Mar 11;112(4):903–12. doi: 10.1016/j.ajhg.2025.02.013 (PMC12081281; doi:10.1016/j.ajhg.2025.02.013)
Supplement: Document S2. Article plus supplemental information [file mmc3.pdf]

The contribution of coding variants to the heritability of multiple cancer types using UK Biobank whole-exome sequencing data

Authors

Naomi Wilcox, Jonathan P. Tyrer, Joe Dennis, Xin Yang, John R.B. Perry, Eugene J. Gardner, Douglas F. Easton

Correspondence

senior_naomi@yahoo.com

We developed a method to estimate the contribution of rare coding variants to cancer heritability and applied it to 11 cancers in the UK Biobank. Our results provide insights into the contribution of genes to multiple cancers. We identify a near-complete overlap of genes for breast, ovarian, prostate, and pancreatic cancer.

Wilcox et al., 2025, The American Journal of Human Genetics *112*, 903–912
April 3, 2025 Crown Copyright © 2025 Published by Elsevier Inc. on behalf of American Society of Human Genetics.
https://doi.org/10.1016/j.ajhg.2025.02.013

CellPress

The contribution of coding variants to the heritability of multiple cancer types using UK Biobank whole-exome sequencing data

Naomi Wilcox,[1,*] Jonathan P. Tyrer,[1] Joe Dennis,[1] Xin Yang,[1] John R.B. Perry,[2,3] Eugene J. Gardner,[3] and Douglas F. Easton[1,4]

Summary

Genome-wide association studies have been highly successful at identifying common variants associated with cancer; however, they do not explain all the inherited risks of cancer. Family-based studies, targeted sequencing, and, more recently, exome-wide association studies have identified rare coding variants in some genes associated with cancer risk, but the overall contribution of these variants to the heritability of cancer is less clear. Here, we describe a method to estimate the genome-wide contribution of rare coding variants to heritability that fits models to the burden effect sizes using an empirical Bayesian approach. We apply this method to the burden of protein-truncating variants in over 15,000 genes for 11 cancers in the UK Biobank using whole-exome sequencing data on over 400,000 individuals. We extend the method to consider the overlap of genes contributing to pairs of cancers. We found ovarian cancer to have the greatest proportion of heritability attributable to protein-truncating variants in genes (46%). The joint cancer models highlight significant clustering of cancer types, including a near-complete overlap in susceptibility genes for breast, ovarian, prostate, and pancreatic cancer. Our results provide insights into the contribution of rare coding variants to the heritability of cancer and identify additional genes with strong evidence of susceptibility to multiple cancer types.

Introduction

Genome-wide association studies (GWASs) have been highly successful at identifying common variants associated with disease. Increasingly, association studies are being extended to study rare variants using next-generation sequencing methods. For example, for breast cancer (MIM: 114480), GWASs have identified over 300 common susceptibility loci,[1–3] while rare variants in *ATM* (MIM: 607585), *BARD1* (MIM: 601593), *BRCA1* (MIM: 113705), *BRCA2* (MIM: 600185), *CHEK2* (MIM: 604373), *RAD51C* (MIM: 602774), *RAD51D* (MIM: 602954), *PALB2* (MIM: 610355), and *TP53* (MIM: 191170) have been identified through linkage or targeted sequencing studies.[4] Exome-wide analysis has recently additionally identified rare variants in *MAP3K1* (MIM: 600982) to be associated with breast cancer risk.[5] Similarly, for bowel cancer (MIM: 114500), GWASs have identified over 200 common susceptibility loci,[6] and rare variants have been identified in mismatch repair (MMR) genes including *MSH2* (MIM: 609309), *MSH6* (MIM: 600678), *MLH1* (MIM: 120436), and *PMS2* (MIM: 600259), as well other genes including *APC*[7] (MIM: 611731). The increasing availability of whole-exome sequencing (WES) and whole-genome sequencing data is enabling exome- and genome-wide analysis of rare variants and the discovery of novel rare variants associated with cancer risk.

For common variants, GWAS data can be used to estimate the overall contribution to heritability, using methods including linkage disequilibrium score regression (LDSC),[8] which uses GWAS summary results, and The Cancer Genome Atlas (TGCA).[9] LDSC has been used to estimate that common variants explain ~41% of the familial relative risk of breast cancer and ~73% of colorectal cancer.[2,6] This method has also been extended to estimate the genetic correlation between traits,[10] which has shown significant correlations in cancer susceptibility, for example, for breast and ovarian cancer (MIM: 167000) and breast and lung cancer[11,12] (MIM: 211980). An analogous question is what is the overall contribution of rare coding variants to cancer heritability. While some genes, such as *BRCA1* and *BRCA2*, have long been known to make a significant contribution to the familial aggregation of certain cancers,[5,13] the more general question has not been definitively answered since most genes have not been extensively evaluated in association studies.

We previously described a method for evaluating the contribution of the gene-wise burden of rare coding variants to cancer heritability that fits models to the burden effect sizes using an empirical Bayesian approach.[5] This approach can be implemented using gene burden summary statistics and is not computationally intensive. We previously applied this method to the burden of protein-truncating variants (PTVs) in genes and breast cancer

[1]Centre for Cancer Genetic Epidemiology, Department of Public Health and Primary Care, University of Cambridge, Cambridge, UK; [2]Metabolic Research Laboratory, Wellcome-MRC Institute of Metabolic Science, University of Cambridge, Cambridge, UK; [3]MRC Epidemiology Unit, Wellcome-MRC Institute of Metabolic Science, University of Cambridge, Cambridge, UK; [4]Centre for Cancer Genetic Epidemiology, Department of Oncology, University of Cambridge, Cambridge, UK
*Correspondence: senior_naomi@yahoo.com
https://doi.org/10.1016/j.ajhg.2025.02.013.
Crown Copyright © 2025 Published by Elsevier Inc. on behalf of American Society of Human Genetics.
This is an open access article under the CC BY license (http://creativecommons.org/licenses/by/4.0/).

Table 1. **Cancer ICD10 codes, the number of subjects with and without cancer, and the model used for analysis**

		Female		Male			
	**ICD10 code(s)**	Subjects without cancer	Subjects with cancer	Subjects without cancer	Subjects with cancer	**FH variable**	**Model**
Breast	C50, D05	209,435	17,958	191,820	94	yes	3
Lung	C34	225,612	1,781	189,959	1,955	yes	3
Prostate	C61	227,393	0	180,214	11,700	yes	3
Bowel	C18, C19, C20, C21	224,404	2,989	187,956	3,958	yes	3
Pancreatic	C25	226,900	493	191,326	588	no	2
Endometrial	C54	225,419	1,974	191,914	0	no	1
Ovarian	C56	226,021	1,372	191,914	0	no	1
Esophagus	C15	227,118	275	191,167	747	no	2
Kidney	C64	226,774	619	190,862	1,052	no	2
Bladder	C67	227,072	321	190,780	1,134	no	2
Malignant melanoma	C43	225,111	2,282	189,986	1,928	no	2

Cancers not previously mentioned: endometrial (MIM: 608089), esophagus (MIM: 133239), kidney (MIM: 144700), bladder (MIM: 109800), and malignant melanoma (MIM: 155600).

risk using data from the UK Biobank and Breast Cancer Association Consortium (BCAC).[5] Here, we apply this method to 11 different cancer types using data from the UK Biobank. We extend this method to consider the overlap of genes contributing to pairs of cancers and evaluate the correlation of coding variant heritability among cancers. This method can be used to derive the posterior probability that each gene is cancer associated.

Material and methods

Material: UK Biobank

The UK Biobank is a population-based prospective cohort study of more than 500,000 individuals. UK Biobank has approval from the North West Multi-centre Research Ethics Committee (MREC) as a Research Tissue Bank (RTB). More detailed information on the UK Biobank is given elsewhere.[14,15] WES data for 450,000 samples were released in October 2021 and accessed via the UK Biobank DNANexus platform.[16] Quality control (QC) metrics were applied to variant call format (VCF) files as described by Gardner et al., including genotype-level filters for depth and genotype quality.[17] Other filters, including the exclusion of samples with disagreement between genetically determined and self-reported sex or excess relatives, were applied as described elsewhere.[5] Analyses were restricted to individuals of European ancestry, as defined by ancestry informative principal components. The final dataset for analysis included 419,307 samples, with 227,393 females and 191,914 males.

Subjects with cancer were determined by linkage to national cancer registration data (NCRAS) and by selecting the appropriate ICD-10 codes (Table 1). For breast cancer, we also included self-reported cancer (7.8% of all subjects with breast cancer) for consistency with previous analysis and since self-reporting a breast cancer diagnosis is known to be accurate.[18] Subjects with prevalent and incident cancer were included. Only cancers that were an individual's first or second diagnosed cancer were included. The numbers of subjects with cancer for males and females for each cancer are provided in Table 1. The 11 cancers we considered were the most common solid tumors by incident cancer diagnoses in the UK Biobank, as per the UK Biobank Malignant Cancer Summary Report.[19]

The Ensembl Variant Effect Predictor (VEP) was used to annotate variants, including the 1000 Genomes phase 3 allele frequency, sequence ontology variant consequences, and exon/intron numbers.[20] Annotation files were used to identify PTVs (including predicted frameshift, stop gain, start loss, and canonical splice variants). PTVs in the last exon of each gene and the last 50 bp of the penultimate exon were excluded, as these are generally predicted to escape nonsense-mediated mRNA decay (NMD).

Methods

Gene burden tests

To test for associations between rare variants in genes and cancers of interest, we performed simple burden tests where variants within genes are collapsed together. This is a powerful method if variants have similar effect sizes.[21] We consider the simplest type of burden test where genotypes are collapsed to a 0/1 variable based on whether the samples carry a PTV, including canonical splice-site variants, in a specific gene. That is, $G_i = 1$ if $\sum_{j=1}^{p} g_{ij} > 0$ and 0 if $\sum_{j=1}^{p} g_{ij} = 0$, where $g_{ij} = 0, 1, 2$ is the number of minor alleles observed for sample i at variant j and p is the number of PTVs in the gene.

To apply the approach above, we fit logistic regression models in which the carrier status is the outcome variable and the disease phenotype a covariate. This method is described further elsewhere.[22]

When there is no family history information available and the cancer is prevalent in only one sex, e.g., ovarian cancer, the model used is

$$\text{model 1} : \log\left(\frac{P(G=1)}{1-P(G=1)}\right) = \alpha + \beta_1 Case + \beta_4 x_4 \ldots$$

When there is no family history information available but the cancer is prevalent in both sexes, e.g., pancreatic cancer (MIM: 260350), the model used is

$$\text{model 2}: \log\left(\frac{P(G=1)}{1-P(G=1)}\right) = \alpha + \beta_1 Case + \beta_3 Sex + \beta_4 x_4 \ldots$$

When family history information is available, e.g., for breast cancer, the model used is

$$\text{model 3}: \log\left(\frac{P(G=1)}{1-P(G=1)}\right) = \alpha + \beta_1 (Case + 0.5FH) + \beta_3 Sex + \beta_4 x_4 \ldots$$

We tested for association using the Wald p value associated with β_1. Highly significant associations based on small counts may be unreliable, so we also conducted likelihood ratio tests for genes reaching exome-wide significance with ≤ 5 affected carriers. For gene-cancer associations with $p < 0.001$, we additionally performed Firth logistic regression to account for potential imprecision in the p values due to small carrier counts.[23]

For breast cancer, the PTV burden results were reported by Wilcox et al.[5] For the other cancers with available family history information, i.e., bowel, lung, and prostate cancer (MIM: 176807), the results for the PTV burden association analysis are shown elsewhere.[22] Here, we additionally present the PTV burden association results for the 7 other cancers without family history information.

Modeling effect sizes

A method to model the effect sizes associated with PTVs, and hence estimate the contribution of PTVs to the familial relative risk of breast cancer, was described by Wilcox et al.[5] Here, we generalize this method for any cancer in the UK Biobank, as well as extend the method to account for the joint distribution of multiple cancers. For breast cancer results, here we use the UK Biobank data only, whereas the paper by Wilcox et al.[5] included the BCAC data in the familial relative risk (FRR) calculations.

Individual cancer model

For individual cancers, we assume a prior distribution for effect sizes (log-odds ratios [OR]) $f(\beta|\alpha,\eta)$ in which a proportion, α, of genes are associated with the cancer. For genes that are risk associated, the prior distribution for the log-OR is assumed to follow a negative exponential distribution. Thus,

$$\beta \sim \begin{cases} 0 \; w.p \; 1-\alpha \\ g(\beta,\eta) \; w.p \; \alpha \end{cases}, \text{where } g(\beta,\eta) \sim \eta e^{-\eta_\beta}.$$

The effect size is assumed to be the same for all PTVs in a gene. Thus, the distribution is determined by the parameters α and η. These parameters can be fit, using maximum likelihood, from summary counts of the numbers of PTV carriers with and without cancer in each gene and each sex. For cancers where family history data is also available, the method can be extended to incorporate counts by family history. Details of the likelihood derivation are given in the supplemental methods and Wilcox et al.[5] The estimates of α and η can be used to derive the posterior probability that each gene is risk associated and the median predicted effect size. It can also be used to estimate the familial relative risk to first-degree relatives (λ) attributable to PTVs in all genes under the assumption that the combined effect of PTVs in different genes is additive.[5,24] We also express this as an estimated proportion of the overall familial relative risk under the assumption that the overall FRR for each cancer is 2 (which is approximately true for all the cancers considered here[25,26]) and that the PTVs combine multiplicatively with other common genetic or familial factors.

To model the joint effect of two cancers, we extend the model to allow four categories of gene: genes associated with cancer 1 only, cancer 2 only, both cancers, or neither cancer, with proportions α_{10}, α_{01}, α_{11}, and $1-\alpha_{10}-\alpha_{01}-\alpha_{11}$ respectively. The effect sizes for the two cancers, when associated, are again assumed to follow negatively exponential distributions. Thus,

$$(\beta_1,\beta_2) \sim \begin{cases} (0,0) & & (1-\alpha_{10}-\alpha_{01}-\alpha_{11}) \\ (g(\beta_1|\eta_1),0) & w.p. & \alpha_{10} \\ (0,g(\beta_2|\eta_2)) & & \alpha_{01} \\ (g(\beta_1|\eta_1),g(\beta_2|\eta_2)) & & \alpha_{11} \end{cases},$$

where $g(\beta_1|\eta_1) \sim \eta_1 \exp(-\eta_1\beta_1), g(\beta_2|\eta_2) \sim \eta_2 \exp(-\eta_2\beta_2)$.

There are thus five parameters to estimate: α_{10}, α_{01}, α_{11}, η_1, and η_2.

For simplicity, we assume that the effect sizes (when both cancers are associated) β_1 and β_2 are uncorrelated. This is motivated by the fact that the data would be too limited to estimate this correlation in addition to the other parameters. Moreover, for the strongest known genes associated with multiple cancers (e.g., *ATM*, *BRCA1*, and *BRCA2*), there is no clear correlation between the effect sizes for different cancers. To evaluate the evidence for overlap in the susceptibility genes for pairs of cancers, we performed likelihood ratio tests against the null hypothesis that the probabilities that genes are associated with each of the two cancers are independent, i.e., the OR:

$$\psi = \frac{\alpha_{11}(1-\alpha_{10}-\alpha_{01}-\alpha_{11})}{\alpha_{10}\alpha_{01}} = 1.$$

More details are provided in the supplemental methods.

Results

PTV burden results

The PTV burden results for 11 all cancers are summarized in Table 2. Association results for each cancer that were not reported previously,[5,27] for genes reaching $p < 0.001$, can be found in Tables S1–S7. The corresponding Manhattan and quantile-quantile (QQ) plots can be found in Figures S1–S14. A comparison of Wald and likelihood ratio test (LRT) p values for genes reaching exome-wide significance from the Wald test and with the number of carriers with cancer ≤ 5 for each cancer are shown in Table S8.

The cancer with the most exome-wide associations by the Wald test was ovarian cancer (9 genes), followed by pancreatic (8 genes) and bladder (8 genes) cancer. We specifically examined tumor-suppressor genes (TSGs) defined by COSMIC since previous analyses of breast cancer indicated that this category was highly enriched.[5] The cancer with the most COSMIC TSGs having $p < 1 \times 10^{-4}$ was breast cancer (8 genes), followed by bowel cancer (6 genes). Among TSGs, *ATM* was associated at $p < 1 \times 10^{-4}$ for breast, prostate, pancreatic, and esophagus cancer;

Table 2. Summary of results for PTV burden tests for 11 cancers

	Wald test					Firth regression
	Number of genes at a *p* value threshold					
Cancer	$p < 0.001$	$p < 1 \times 10^{-4}$	$p < 2.5 \times 10^{-6}$	Genes with $p < 2.5 \times 10^{-6}$	COSMIC TSGs with $p < 1 \times 10^{-4}$	Genes with $p < 2.5 \times 10^{-6}$
Breast	30	9	6	*BRCA2, BRCA1, CHEK2, PALB2, ATM, MAP3K1*	*BRCA2, BRCA1, CHEK2, PALB2, ATM, MAP3K1, LZTR1, BARD1*	*BRCA2, BRCA1, PALB2, CHEK2, ATM, MAP3K1*
Prostate	35	8	3	*BRCA2, CHEK2, ATM*	*BRCA2, CHEK2, ATM*	*BRCA2, CHEK2, ATM*
Bowel	42	9	5	*MSH6, MSH2, MLH1, APC, GAPDH*	*MSH6, MSH2, MLH1, APC, FLCN, SMAD4*	*MSH6, MSH2, MLH1, APC*
Lung	46	8	0	N/A	*ARHGAP35, BIRC3*	N/A
Pancreatic	100	38	8	*ATM, MEN1, RCN2, YPEL3, SMC2, SEC14L3, GNG10, ZNF461*	*ATM, MEN1*	*ATM*
Endometrial	80	26	5	*MSH6, MLH1, ACRV1, STK32C, PSMC6*	*MSH6, MLH1, MSH2*	*MSH6*
Ovarian	115	35	9	*BRCA2, BRCA1, IVD, JAML, KCNAB2, ZFP14, TMEM163, TMEM167A, NHEJ1*	*BRCA2, BRCA1, NFRSF14*	*BRCA2, BRCA1*
Esophagus	117	32	2	*KNL1, IRF2BP2*	*KNL1, ATM, FUS*	N/A
Kidney	89	27	6	*PKD1, FGL2, TTC9, EX0C7, NCK2, TMEM174*	N/A	N/A
Bladder	72	27	8	*DLX2, ZNF506, CDCP2, TMEM222, KDM1A, ARHGEF6, HR, NLRP10*	N/A	N/A
Malignant melanoma	45	12	4	*DCX, MED9, MRPL44, CDKN2A*	*CDKN2A*	*MED9*

Genes not previously mentioned: *LZTR1* (MIM: 600574), *GAPDH* (MIM: 138400), *FLCN* [MIM: 607273), *SMAD4* (MIM: 600993), *ARHGAP35* (MIM: 605277), *BIRC3* (MIM: 601721), *MEN1* (MIM: 613733), *RCN2* (MIM: 602584), *YPEL3* (MIM: 609724), *SMC2* (MIM: 605576), *SEC14L3* (MIM: 612824), *GNG10* (MIM: 604389), *ZNF461* (MIM: 608640), *ACRV1* (MIM: 102525), *STK32C* (MIM: N/A), *PSMC6* (MIM: 602708), *IVD* (MIM: 607036), *JAML* (MIM: 609770), *KCNAB2* (MIM: 601142), *ZFP15* (MIM: 620163), *TMEM163* (MIM: 618978), *TMEM167A* (MIM: 620000), *NHEJ1* (MIM: 611290), *NFRSF14* (MIM: N/A), *KNL1* (MIM: 609173), *IRF2BP2* (MIM: 615332), *FUS* (MIM: 137070), *PKD1* (MIM: 601313), *FGL2* (MIM: 605351), *TTC9* (MIM: 610488), *EX0C7* (MIM: 608163), *NCK2* (MIM: 604930), *TMEM174* (MIM: 614909), *DLX2* (MIM: 126255), *ZNF506* (MIM: N/A), *CDCP2* (MIM: 612320), *TMEM222* (MIM: 619469), *KDM1A* (MIM: 609132), *ARHGEF6* (MIM: 300267), *HR* (MIM: 602302), *NLRP10* (MIM: 609662), *DCX* (MIM: 300121), *MRPL44* (MIM: 611849), and *CDKN2A* (MIM: 600160). The table includes the number of genes reaching different significance thresholds as well as the list of genes that reach exome-wide significance and COSMIC TSGs with Wald test $p < 1 \times 10^{-4}$ listed in ascending *p* value order. For breast cancer, the results are from the meta-analysis of the UK Biobank and BCAC datasets as reported by Wilcox et al.,[5] apart from for Firth regression, which was just run in the UK Biobank. The final column shows the genes that remained exome wide significant using Firth regression. N/A, not applicable.

Table 3. Optimized values of α and η for each cancer, as well as the estimated heritability and contribution to familial relative risk

	α	η	Median OR	loglik	λ	% FRR	Genes with posterior probability >0.9	Genes with posterior probability >0.5
Breast	0.0027	1.8	1.5	713.43	1.06	8.8	*BRCA1*, *BRCA2*, *CHEK2*, *PALB2*, *ATM*, *MAP3K1*	*BRCA1*, *BRCA2*, *CHEK2*, *PALB2*, *ATM*, *MAP3K1*
Prostate	0.020	6.2	1.1	37.73	1.01	1.08	*BRCA2*, *CHEK2*, *ATM*	*BRCA2*, *CHEK2*, *ATM*
Bowel	0.0016	1.2	1.8	140.67	1.05	6.7	*MSH2*, *MSH6*, *MLH1*, *APC*	*MSH2*, *MSH6*, *MLH1*, *APC*, *GAPDH*
Lung	0.0035	7.4	1.1	0.04	1.00	0.1	N/A	N/A
Pancreas	0.019	2.8	1.3	7.26	1.08	10.6	*ATM*	*ATM*
Endometrial	0.0014	1.3	1.7	88.05	1.12	16.5	*MSH6*	*MSH6*
Ovarian	0.037	2.4	1.3	89.26	1.37	45.9	*BRCA2*, *BRCA1*	*BRCA2*, *BRCA1*, *IVD*, *SLC35E4*, *REX05*
Esophagus	0.030	3.6	1.2	0.92	1.04	6.2	N/A	*NLRP12*, *ATM*
Kidney	0.00	4.4	1.2	0.00	1.00	0.0	N/A	N/A
Bladder	0.00	2.5	1.3	0.00	1.00	0.0	N/A	N/A
Malignant melanoma	0.00	5.6	1.1	0.00	1.00	0.0	N/A	N/A

Genes not previously mentioned: *SLC35E4* (MIM: N/A), *REX05* (MIM: N/A), and *NLRP12* (MIM: 609648). The %FRR estimates use posterior gene PTV frequency adjusted for copy-number variation (CNV) frequency. The genes with posterior probability >0.5 are listed in descending order. N/A, not applicable.

BRCA2 was associated at $p < 1 \times 10^{-4}$ for breast, prostate, and ovarian cancer; and *MSH6*, *MSH2*, and *MLH1* were associated at $p < 1 \times 10^{-4}$ for bowel and endometrial cancer. No COSMIC TSGs had $p < 1 \times 10^{-4}$ for kidney or bladder cancer. Cancers with >50 genes associated at $p < 0.001$ include pancreatic, endometrial, ovarian, esophagus, kidney, and bladder cancer.

We note that for that for many of the associated genes, the number of carriers with cancer was low. In this situation, the p values can be exaggerated and, as expected, were somewhat less significant using Firth regression or likelihood ratio tests. Of the gene-disease associations not reported previously, associations reaching exome-wide associations using all methods included *MSH6* for endometrial cancer, *ATM* for prostate cancer, and *MED9* (MIM: 609878) for melanoma.

Of the genes not listed in Table 2, of interest for ovarian cancer are associations at $p < 0.05$ for 5 putative ovarian cancer genes: *MSH6* ($p = 0.00056$), *BRIP1* (MIM: 605882) ($p = 0.00055$), *RAD51C* ($p = 0.028$), *RAD51D* ($p = 0.00011$), and *CHEK2* ($p = 0.00049$).

Empirical Bayes modeling

Table 3 and Figure 1 summarize the best-fitting models for each cancer. The cancer with the greatest estimated proportion of risk-associated genes was ovarian cancer ($\alpha = 0.037$, equivalent to 578 genes), followed by esophageal cancer ($\alpha = 0.030$, ≈468 genes) and pancreatic cancer ($\alpha = 0.019$, ≈297 genes). In contrast, for kidney, bladder, and malignant melanoma, α was estimated to be 0. The estimated exponential distributions for the log-ORs are shown in Figure 1. The graph is least steep for bowel cancer, which has the greatest estimated median OR. In contrast, the median OR was lowest for prostate cancer, consistent with a lower proportion of higher-risk genes.

In terms of overall contribution to the FRR, the highest proportion was for ovarian cancer (45.9%), followed by endometrial cancer (16.5%; Table 3).

Based on the best-fitting models, we computed the posterior probability of each gene being associated with each cancer (Tables S9–S16). For breast cancer, there were 6 genes with a posterior probability > 0.9: *BRCA2*, *BRCA1*, *PALB2*, *CHEK2*, *ATM*, and *MAP3K1* (Table S9). *BRCA2* and *BRCA1* also reached this level for ovarian cancer, *BRCA2*, *ATM*, and *CHEK2* for prostate cancer, and *ATM* for pancreatic cancer. For bowel cancer, there were 4 genes with a posterior probability > 0.9: the MMR genes *MLH1*, *MSH6*, and *MSH2*, as well as *APC* (Table S10). *MSH6* also reached this level for endometrial cancer. For lung, esophagus, kidney, and bladder cancer, as well as malignant melanoma, there were no genes with a posterior probability > 0.9. Table 4 shows all genes reaching posterior probability >0.8 for at least one cancer.

Joint cancer models

We next considered models incorporating pairs of cancers (Figures 2 and 3). The strongest evidence for overlap was found for breast-prostate (likelihood ratio $p = 1.5 \times 10^{-9}$), breast-ovarian ($p = 2.1 \times 10^{-8}$), bowel-endometrial ($p = 3.0 \times 10^{-8}$), and breast-pancreatic ($p = 2.3 \times 10^{-5}$). Associations with $p < 0.001$ were additionally observed for prostate-ovarian and prostate-pancreas, with weaker evidence of overlap for breast-bowel, breast-lung, breast-esophagus, and lung-pancreas. More detailed results for pairs with $p < 0.01$ are shown in Table 5 and for all cancer pairs in Table S17.

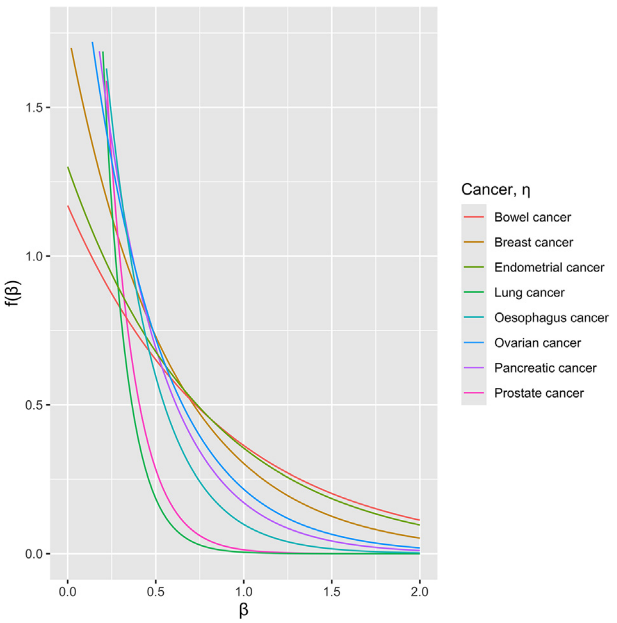

Figure 1. The distributions for the log-odds ratio of associated genes for each cancer
This uses the optimized value of η, where $g(\beta,\eta) \sim \eta e^{-\eta_\beta}$.

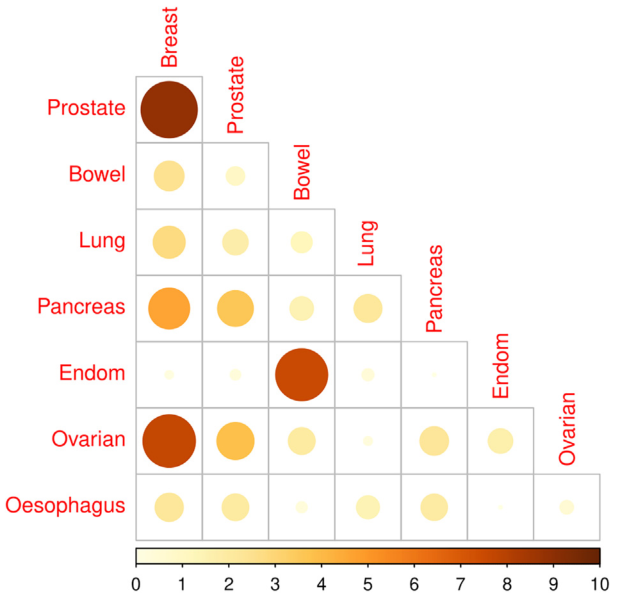

Figure 2. -log10 *p* values from the LRT comparing joint to independent cancer models
-log10 *p* values from a likelihood ratio test comparing models in which the prior probabilities that any gene is associated with each of a pair of cancers is related, with corresponding models in which these probabilities are independent (see methods).

We note that of the cancer pairs with $p < 0.001$, the breast-prostate, breast-ovarian, breast-pancreatic, prostate-pancreatic, and pancreatic-ovarian pairs all estimated $\alpha_{10} = \alpha_{01} = 0$, i.e., a complete overlap of associated genes. Genes with a posterior probability > 0.8 of being associated with both cancers for at least 1 cancer pair were *APC*, *ATM*, *BAP1* (MIM: 603089), *BRCA1*, *BRCA2*, *CHEK2*, *MAP3K1*, *MLH1*, *MSH2*, *MSH6*, and *PALB2* (Table S18). *ATM* was associated with the most cancer pairs with a posterior probability > 0.8 (9 pairs). Six of these genes are established genes for one or more of the cancers, the exception being *BAP1*, which had a posterior probability of 0.824 in the breast-prostate model. In the breast-ovarian model, the gene with the next highest posterior probability (after those identified above) was *NHEJ1* (0.300), followed by the established risk genes *RAD51D* (0.282) and *BRIP1* (0.259) (Table S19). *NHEJ1* PTVs were associated with an OR of 2.70 (1.30, 5.61), $p = 0.0079$, for breast cancer and 17.67 (5.36, 58.24), $p = 2.37 \times 10^{-6}$, for ovarian cancer.

Table 4. Genes with posterior probability >0.8 for at least 1 cancer

	Cancers
APC	bowel
ATM	breast, pancreas, prostate
BRCA1	breast, ovarian
BRCA2	breast, ovarian, prostate
CHEK2	breast, prostate
MAP3K1	breast
MLH1	bowel
MSH2	bowel
MSH6	bowel, endometrial
PALB2	breast

The cancer columns are the cancers that had a posterior probability > 0.8 of the gene being associated with the cancer.

For bowel and endometrial cancer, the best joint model estimated $\alpha_1 = 0.00020$ ($\approx$3 genes), $\alpha_2 = 0$, and $\alpha_3 = 0.00140$ ($\approx$22 genes), i.e., consistent with subsets of genes associated with both cancers and with bowel cancer only but none associated with endometrial cancer alone. There were 3 genes with a posterior probability > 0.9 of being associated with both cancers: *MSH6* (1.00), *MLH1* (0.98), and *MSH2* (0.98) (Table S21). COSMIC TSGs with a posterior probability > 0.5 also include *APC* (0.52). The genes with the highest posterior probability of being associated with bowel cancer alone were *APC* (0.48) and *GAPDH* (0.48).

Discussion

We used a large exome-sequencing dataset to describe the pattern of gene-based associations across 11 cancer types. For cancers with results not previously reported elsewhere, we found exome-wide significant genes for pancreatic, endometrial, ovarian, esophagus, kidney, and bladder cancer, as well as for malignant melanoma, based on the Wald test. Many of these associations were, however, based on low carrier counts, and the associations were less significant based on the more conservative Firth regression or a

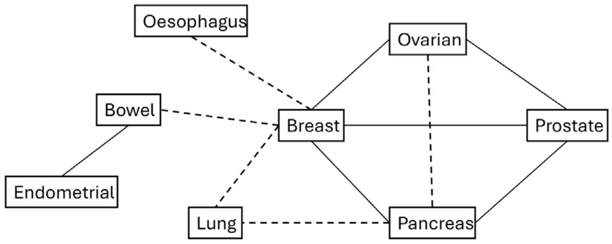

Figure 3. Diagram showing cancer pairs with LRT $p \leq 0.001$ as bold lines and LRT $0.01 \leq p < 0.001$ as dashed lines

likelihood ratio test: of gene-disease associations not previously reported, only *MED9* for melanoma reached exome-wide significance.

Previous studies, such as Backman et al.[16] and Wang et al.,[28] have demonstrated the utility of phenome-wide association studies (PheWAS) to identify associations with traits and rare coding variants. Our study highlights additional associations that were not identified in these studies. Our analysis uses a Bayesian approach to estimate the posterior probability of a gene being associated with a cancer, or a pair of cancers, and enables the identification of additional genes to be associated with risk that would have been missed by a simple trait-gene burden test. For example, we identified *ATM* to be associated with 9 cancer pairs with a posterior probability > 0.8 (involving bowel, lung, and esophageal cancer in addition to breast, prostate, ovary, and pancreas), while it was only associated with breast cancer by Backman et al.[16] and Wang et al.[28]

Our study highlights the potential value of combining information for multiple cancers. We found strong evidence for overlap in susceptibility genes between several cancer pairs. This information can strengthen the evidence for susceptibility to one cancer by borrowing information from the other cancer, in effect improving the prior. Thus, we found the data to be consistent with an essentially complete overlap of susceptibility genes for breast, ovarian, pancreatic, and prostate cancer. While this is not surprising given that several of the known susceptibility genes are associated with several of these cancers (though not to the same extent), the reasons underlying the overlap in susceptibility to these cancers are not well understood, and the results thus suggest avenues for future research.

The results also highlight some additional putative susceptibility genes. Of particular interest was *NHEJ1*, which reached exome-wide significance in ovarian cancer analysis for the Wald test and showed evidence of association with breast cancer. The latter association would have been missed if considered in isolation. *NHEJ1* encodes a DNA repair factor essential for non-homologous end joining (NHEJ). The association for PTVs is consistent with NHEJ being defective in a large proportion of ovarian cancers.[29] Ovarian cancer cells defective in NHEJ have also been shown to be resistant to PARP inhibition,[29] which might indicate some relevance for therapeutic approaches. We also found stronger evidence for *BAP1* in the joint analysis of breast and prostate cancer. *BAP1* PTVs have been associated with a range of cancers, including cutaneous and uveal melanoma, kidney cancer, mesothelioma, and basal cell carcinoma. Kidney ($p = 0.059$) and bladder ($p = 0.047$) cancer, as well as ovarian ($p = 0.033$) and pancreatic ($p = 0.022$) cancer, also showed some evidence of association in this dataset (Table S30; Figure S15).

Beyond the breast, ovarian, pancreatic, and prostate associations and the expected overlap between bowel and endometrial cancer, driven by the MMR genes, there were also significant, albeit weaker, overlaps between breast cancer and several other cancers. This is partly driven by *ATM*, which was associated with 9 pairs as mentioned above. The best-fitting models for lung cancer in combination with breast, pancreatic, and prostate

Table 5. Estimated parameters for the best fitting joint cancer models and the LRT p value comparing the joint to independent cancer models

Cancer 1	Cancer 2	Method	α_{10}	α_{01}	α_{11}	η_1	η_2	LRT
Breast	Prostate	1	0.00000	0.00000	0.00220	1.49	2.51	1.5×10^{-9}
Breast	Ovarian	1	0.00000	0.00000	0.00270	1.77	1.23	2.1×10^{-8}
Bowel	Endometrial	2	0.00020	0.00000	0.00140	1.13	0.93	3.0×10^{-8}
Breast	Pancreas	1	0.00000	0.00000	0.00230	1.62	1.57	2.3×10^{-5}
Prostate	Ovarian	1	0.00000	0.00340	0.01700	6.04	2.06	0.00015
Prostate	Pancreas	1	0.00000	0.00000	0.00840	4.56	2.17	0.00028
Breast	Lung	1	0.00013	0.00026	0.00150	1.52	6.00	0.0014
Breast	Bowel	1	0.00053	0.00027	0.00160	1.51	1.00	0.0035
Pancreas	Ovarian	1	0.00000	0.00000	0.03000	3.09	2.25	0.0054
Breast	Esophagus	1	0.00000	0.00980	0.00240	1.64	2.95	0.0062
Lung	Pancreas	1	0.00000	0.00000	0.00460	5.93	2.05	0.0069

Method refers to the joint cancer model method used as described in the material and methods section. Results here have LRT p value values < 0.01 and are sorted by ascending LRT p value. $\alpha_{10} = P(C1\, n\, C2')$, $\alpha_{01} = P(C1' n\, C2)$, and $\alpha_{11} = P(C1\, n\, C2)$. η_1 and η_2, effect size parameters for cancer 1 and cancer 2, respectively.

cancer are all consistent, with most susceptibility genes for these cancers also being associated with lung cancer, albeit to a smaller extent.

The results confirm the striking enrichment of tumor-suppressor and DNA repair genes among cancer susceptibility genes. Thus, of the 19 genes with a posterior probability > 0.5 in any analyses, 12 are known TSGs and/or involved in DNA repair (including all 11 genes with a posterior probability > 0.8). In terms of the contribution of PTVs to the FRR of each cancer, the proportion was estimated to be greatest for ovarian cancer (46%), followed by endometrial cancer (16.5%). The contribution was estimated to be 0% for kidney and bladder cancer and malignant melanoma. This is clearly inaccurate: *CDKN2A* and *CDK4* (MIM: 123829) PTVs are associated with malignant melanoma,[30,31] and *VHL* (MIM: 608537) is associated with kidney cancer.[32] (*CDKN2A* did reach exome-wide significance for association with melanoma in this dataset using a Wald test, but this is likely to have been exaggerated, as the likelihood ratio p value was 0.00041.) *CDK4* had 0 carriers with melanoma, while *VHL* had 1 carrier with kidney cancer, and the OR was non-significant (3.5 [0.49, 25.4], $p = 0.21$). Larger datasets should allow more precise estimates of the associations between these genes and cancer risk to be derived. The UK Biobank has lower incidence rates for some cancers (e.g., kidney and lung), reflecting a "healthy volunteer" bias.[33] While this is unlikely to result in any significant bias in the association analyses, studies in populations with incidence rates would also improve power. Notwithstanding these uncertainties, the estimated contribution to the FRR, for all cancers, was largely attributable to the known genes. This strongly suggests that the contribution of additional genes is likely to be small and that most "missing" heritability is likely to reflect non-coding variation.

Similar to the breast cancer results reported previously, there was an excess of associations at $p < 0.001$ across the cancers, indicating that further genes should be identifiable in larger datasets. We also note that for many cancers, the sample size is small, resulting in large standard errors and wide confidence intervals for many associated genes. Estimated risk estimates for associated genes not previously established may also be over-estimated due to the "winners curse."[34] Further replication in larger datasets will, therefore, be necessary to confirm associations and provide more precise risk estimates for variants in associated genes. Furthermore, our study focused on individuals of European ancestry, and expanding the analysis to other populations will be important and may identify additional associations. Some other large cohorts, e.g., All of Us, could provide a basis for replicating the findings across multiple cancers in the future; All of Us also has more diverse ancestry, with 51% of individuals being of non-European ancestry.[35] Alternatively, large targeted gene panel studies may be a more feasible option for individual cancers.

Our analyses have some limitations. We restricted our analyses to PTVs. For the known genes, most of the effect is driven by PTVs, and the assumption that PTVs confer similar risks is a plausible simplification. In principle, the analyses could be extended to missense or other coding variants, but this would require the model to be extended to incorporate variation in effect size. Our multicancer analyses have thus far been restricted to pairs of cancers but could logically be extended to larger sets of cancers. This would provide a more rational model, defining a single set of parameters and posterior probabilities. However, it would require a larger number of parameters to be estimated simultaneously. The model also assumed a particular prior distribution of effect sizes, in which a proportion of genes are associated with risk—an example of a spike-and-slab prior.[36] This is somewhat analogous to the approach used in some GWAS analyses, for example, LDpred2.[37] The model also only accounts for rare variants associated with an increased risk and does not account for protective alleles. We note, however, that all 47 exome-wide significant associations were positive, so this appears a reasonable simplification given the available data. The model is simplistic and may not reflect the true underlying distribution for all cancers; however, the model provides a systematic approach to identify and rank genes worthy of exploration in larger targeted sequencing and functional experiments.

In conclusion, we have developed an approach to estimate the genome-wide contribution of the burden of rare coding variants to the heritability of cancer, considering 11 cancers in the UK Biobank. We have shown significant clustering of cancer types, including breast, ovarian, prostate, and pancreatic cancer, with a large enrichment of tumor-suppressor and DNA repair genes among cancer susceptibility genes. The estimated contribution to the FRR, for all cancers, was largely attributable to the known genes. This strongly suggests that the contribution of additional genes is likely to be small and that most missing heritability is likely to reflect non-coding variation.

Data and code availability

Requests for access to UK Biobank data should be made to the UK Biobank access management team (access@ukbiobank.ac.uk). QC filtering of VCF files was performed using vcftools v.0.1.15, bcftools v.1.9, picard v.2.22.2, and plink v.1.90b, as outlined in the material and methods. Variants were annotated using Ensembl VEP v.101 with assembly GRCh38. The code for each software is available at the website of each package. Data manipulation and analysis were performed using R-4.3.3 with the packages clusterProfiler (4.2.2), data.table (1.14.2), dplyr (1.0.9), dbplyr (2.5.0), gtools (3.9.5), HGNChelper (0.8.9), SKAT (2.2.5), tibble (3.2.1), and tidyr (1.3.1). Plots were created using the additional packages ggplot2 (3.5.1) and ggrepel (0.9.5). The code for each of the R packages can be found in their associated vignettes. Burden test results are available for each cancer from the GWAS Catalog (https://www.ebi.ac.uk/gwas/; https://ftp.ebi.ac.uk/pub/databases/gwas/summary_statistics/). The accession numbers for the burden test results reported in this paper are GWAS Catalog: GCST90503274 (pancreatic cancer), GCST90503275 (endometrial cancer), GCST90503276 (ovarian cancer), GCST90503277 (oesophagus

cancer), GSCST90503278 (kidney cancer), GCST90503279 (bladder cancer), and GCST90503280 (malignant melanoma).

Acknowledgments

QC of the UK Biobank sequencing data was funded by the Medical Research Council (unit programs: MC_UU_12015/2 and MC_UU_00006/2). The research was conducted using the UK Biobank Resource under application no. 28126. N.W. was supported by the International Alliance for Cancer Early Detection, an alliance between Cancer Research UK (C14478/A29329), the Canary Center at Stanford University, the University of Cambridge, the OHSU Knight Cancer Institute, University College London, and the University of Manchester. J.D. was supported by core funding from the NIHR Cambridge Biomedical Research Centre (NIHR203312). X.Y. and J.P.T. were supported by Cancer Research UK (PPRPGM-Nov20\100002 and PRCPJT-May21\100006).

Author contributions

D.F.E. supervised this work and directed the overall analysis. N.W. performed the statistical analysis. N.W., E.J.G., J.P.T., and J.D.P. developed the bioinformatics and computational pipelines. X.Y. and J.D. acquired data, and X.Y. extracted cancer phenotypes. N.W. and D.F.E. drafted the manuscript. All authors reviewed and approved the paper.

Declaration of interests

N.W. has been an employee and shareholder of Illumina since October 1, 2024. J.R.B.P. and E.J.G. are employees of Insmed Innovation UK and hold stock/stock options in Insmed, Inc. J.R.B.P. also receives research funding from GSK and engages in paid consultancy for WW International, Inc.

Supplemental information

Supplemental information can be found online at https://doi.org/10.1016/j.ajhg.2025.02.013.

Web resources

OMIM, http://www.omim.org

Received: September 27, 2024
Accepted: February 12, 2025
Published: March 11, 2025

References

1. Zhang, H., Ahearn, T.U., Lecarpentier, J., Barnes, D., Beesley, J., Qi, G., Jiang, X., O'Mara, T.A., Zhao, N., Bolla, M.K., et al. (2020). Genome-wide association study identifies 32 novel breast cancer susceptibility loci from overall and subtype-specific analyses. Nat. Genet. *52*, 572–581. https://doi.org/10.1038/s41588-020-0609-2.
2. Michailidou, K., Lindström, S., Dennis, J., Beesley, J., Hui, S., Kar, S., Lemaçon, A., Soucy, P., Glubb, D., Rostamianfar, A., et al. (2017). Association analysis identifies 65 new breast cancer risk loci. Nature *551*, 92–94. https://doi.org/10.1038/nature24284.
3. Shu, X., Long, J., Cai, Q., Kweon, S.-S., Choi, J.-Y., Kubo, M., Park, S.K., Bolla, M.K., Dennis, J., Wang, Q., et al. (2020). Identification of novel breast cancer susceptibility loci in meta-analyses conducted among Asian and European descendants. Nat. Commun. *11*, 1217. https://doi.org/10.1038/s41467-020-15046-w.
4. Dorling, L., Carvalho, S., González-Neira, A., González-Neira, A., Wahlström, C., Wahlström, C., Pooley, K.A., Parsons, M.T., Fortuno, C., et al.; Breast Cancer Association Consortium (2021). Breast Cancer Risk Genes — Association Analysis in More than 113,000 Women. N. Engl. J. Med. *384*, 428–439. https://doi.org/10.1056/nejmoa1913948.
5. Wilcox, N., Dumont, M., González-Neira, A., Carvalho, S., Joly Beauparlant, C., Crotti, M., Luccarini, C., Soucy, P., Dubois, S., Nuñez-Torres, R., et al. (2023). Exome sequencing identifies breast cancer susceptibility genes and defines the contribution of coding variants to breast cancer risk. Nat. Genet. *55*, 1435–1439. https://doi.org/10.1038/s41588-023-01466-z.
6. Fernandez-Rozadilla, C., Timofeeva, M., Chen, Z., Law, P., Thomas, M., Schmit, S., Díez-Obrero, V., Hsu, L., Fernandez-Tajes, J., Palles, C., et al. (2023). Deciphering colorectal cancer genetics through multi-omic analysis of 100,204 cases and 154,587 controls of European and east Asian ancestries. Nat. Genet. *55*, 89–99. https://doi.org/10.1038/s41588-022-01222-9.
7. Liu, Q., and Tan, Y.Q. (2019). Advances in Identification of Susceptibility Gene Defects of Hereditary Colorectal Cancer. J. Cancer *10*, 643–653. https://doi.org/10.7150/jca.28542.
8. Bulik-Sullivan, B.K., Loh, P.R., Finucane, H.K., Ripke, S., Yang, J., Schizophrenia Working Group of the Psychiatric Genomics Consortium, Patterson, N., Daly, M.J., Price, A.L., and Neale, B.M. (2015). LD Score regression distinguishes confounding from polygenicity in genome-wide association studies. Nat. Genet. *47*, 291–295. https://doi.org/10.1038/ng.3211.
9. Li, T., Ning, Z., Yang, Z., Zhai, R., Zheng, C., Xu, W., Wang, Y., Ying, K., Chen, Y., and Shen, X. (2021). Total genetic contribution assessment across the human genome. Nat. Commun. *12*, 2845. https://doi.org/10.1038/s41467-021-23124-w.
10. Bulik-Sullivan, B., Finucane, H.K., Anttila, V., Gusev, A., Day, F.R., Loh, P.-R., Duncan, L., et al.; ReproGen Consortium; Psychiatric Genomics Consortium; and Genetic Consortium for Anorexia Nervosa of the Wellcome Trust Case Control Consortium 3 (2015). An atlas of genetic correlations across human diseases and traits. Nat. Genet. *47*, 1236–1241. https://doi.org/10.1038/ng.3406.
11. Guo, H., Cao, W., Zhu, Y., Li, T., and Hu, B. (2023). A genome-wide cross-cancer meta-analysis highlights the shared genetic links of five solid cancers. Front. Microbiol. *14*, 1116592. https://doi.org/10.3389/fmicb.2023.1116592.
12. Jiang, X., Finucane, H.K., Schumacher, F.R., Schmit, S.L., Tyrer, J.P., Han, Y., Michailidou, K., Lesseur, C., Kuchenbaecker, K.B., Dennis, J., et al. (2019). Shared heritability and functional enrichment across six solid cancers. Nat. Commun. *10*, 431. https://doi.org/10.1038/s41467-018-08054-4.
13. Peto, J., Collins, N., Barfoot, R., Seal, S., Warren, W., Rahman, N., Easton, D.F., Evans, C., Deacon, J., and Stratton, M.R. (1999). Prevalence of BRCA1 and BRCA2 gene mutations in patients with early-onset breast cancer. J. Natl. Cancer Inst. *91*, 943–949. https://doi.org/10.1093/jnci/91.11.943.
14. Sudlow, C., Gallacher, J., Allen, N., Beral, V., Burton, P., Danesh, J., Downey, P., Elliott, P., Green, J., Landray, M., et al. (2015). UK Biobank: An Open Access Resource for Identifying the Causes of a Wide Range of Complex Diseases of Middle

and Old Age. PLoS Med. *12*, e1001779. https://doi.org/10.1371/journal.pmed.1001779.

15. Collins, R. (2012). What makes UK Biobank special? Lancet *379*, 1173–1174. https://doi.org/10.1016/s0140-6736(12)60404-8.
16. Backman, J.D., Li, A.H., Marcketta, A., Sun, D., Mbatchou, J., Kessler, M.D., Benner, C., Liu, D., Locke, A.E., Balasubramanian, S., et al. (2021). Exome sequencing and analysis of 454,787 UK Biobank participants. Nature *599*, 628–634. https://doi.org/10.1038/s41586-021-04103-z.
17. Gardner, E.J., Kentistou, K.A., Stankovic, S., Lockhart, S., Wheeler, E., Day, F.R., Kerrison, N.D., Wareham, N.J., Langenberg, C., O'Rahilly, S., et al. (2022). Damaging Missense Variants in *IGF1R* Implicate a Role for IGF-1 Resistance in the Aetiology of Type 2 Diabetes (Cold Spring Harbor Laboratory).
18. D'Aloisio, A.A., Nichols, H.B., Hodgson, M.E., Deming-Halverson, S.L., and Sandler, D.P. (2017). Validity of self-reported breast cancer characteristics in a nationwide cohort of women with a family history of breast cancer. BMC Cancer *17*, 692. https://doi.org/10.1186/s12885-017-3686-6.
19. Biobank, U. (2024). UK Biobank Malignant Cancer Summary Report. https://biobank.ndph.ox.ac.uk/~bbdatan/CancerSummaryReport.html.
20. McLaren, W., Gil, L., Hunt, S.E., Riat, H.S., Ritchie, G.R.S., Thormann, A., Flicek, P., and Cunningham, F. (2016). The Ensembl Variant Effect Predictor. Genome Biol. *17*, 122. https://doi.org/10.1186/s13059-016-0974-4.
21. Lee, S., Lin, X., Abecasis, G., and Boehnke, M. (2014). Rare-Variant Association Analysis: Study Designs and Statistical Tests. Am. J. Hum. Genet. *95*, 5–23. https://doi.org/10.1016/j.ajhg.2014.06.009.
22. Wilcox, N., Tyrer, J.P., Dennis, J., Yang, X., Perry, J.R.B., Gardner, E.J., and Easton, D.F. (2025). Using Family History Data to Improve the Power of Association Studies: Application to Cancer in UK Biobank. Genet. Epidemiol. *49*, e22609. https://doi.org/10.1002/gepi.22609.
23. FIRTH, D. (1993). Bias reduction of maximum likelihood estimates. Biometrika *80*, 27–38. https://doi.org/10.1093/biomet/80.1.27.
24. Risch, N. (1990). Linkage strategies for genetically complex traits. I. Multilocus models. Am. J. Hum. Genet. *46*, 222–228.
25. Hemminki, K., Rawal, R., Chen, B., and Bermejo, J.L. (2004). Genetic epidemiology of cancer: From families to heritable genes. Int. J. Cancer *111*, 944–950. https://doi.org/10.1002/ijc.20355.
26. Goldgar, D.E., Easton, D.F., Cannon-Albright, L.A., and Skolnick, M.H. (1994). Systematic Population-Based Assessment of Cancer Risk in First-Degree Relatives of Cancer Probands. J. Natl. Cancer Inst. *86*, 1600–1608. https://doi.org/10.1093/jnci/86.21.1600.
27. Wilcox, N., Tyrer, J.P., Dennis, J., Yang, X., Perry, J.R.B., Gardner, E.J., and Easton, D.F. (2024). Using Family History Data to Improve the Power of Association Studies: Application to Cancer in UK Biobank. Preprint at medRxiv. https://doi.org/10.1101/2024.07.01.24309759.
28. Wang, Q., Dhindsa, R.S., Carss, K., Harper, A.R., Nag, A., Tachmazidou, I., Vitsios, D., Deevi, S.V.V., Mackay, A., Muthas, D., et al. (2021). Rare variant contribution to human disease in 281,104 UK Biobank exomes. Nature *597*, 527–532. https://doi.org/10.1038/s41586-021-03855-y.
29. McCormick, A., Donoghue, P., Dixon, M., O'Sullivan, R., O'Donnell, R.L., Murray, J., Kaufmann, A., Curtin, N.J., and Edmondson, R.J. (2017). Ovarian Cancers Harbor Defects in Nonhomologous End Joining Resulting in Resistance to Rucaparib. Clin. Cancer Res. *23*, 2050–2060. https://doi.org/10.1158/1078-0432.Ccr-16-0564.
30. Rossi, M., Pellegrini, C., Cardelli, L., Ciciarelli, V., Di Nardo, L., and Fargnoli, M.C. (2019). Familial melanoma: diagnostic and management implications. Dermatol. Pract. Concept. *9*, 10–16. https://doi.org/10.5826/dpc.0901a03.
31. Zocchi, L., Lontano, A., Merli, M., Dika, E., Nagore, E., Quaglino, P., Puig, S., and Ribero, S. (2021). Familial Melanoma and Susceptibility Genes: A Review of the Most Common Clinical and Dermoscopic Phenotypic Aspect, Associated Malignancies and Practical Tips for Management. J. Clin. Med. *10*, 3760. https://doi.org/10.3390/jcm10163760.
32. Cowey, C.L., and Rathmell, W.K. (2009). VHL gene mutations in renal cell carcinoma: role as a biomarker of disease outcome and drug efficacy. Curr. Oncol. Rep. *11*, 94–101. https://doi.org/10.1007/s11912-009-0015-5.
33. Fry, A., Littlejohns, T.J., Sudlow, C., Doherty, N., Adamska, L., Sprosen, T., Collins, R., and Allen, N.E. (2017). Comparison of Sociodemographic and Health-Related Characteristics of UK Biobank Participants With Those of the General Population. Am. J. Epidemiol. *186*, 1026–1034. https://doi.org/10.1093/aje/kwx246.
34. Lohmueller, K.E., Pearce, C.L., Pike, M., Lander, E.S., and Hirschhorn, J.N. (2003). Meta-analysis of genetic association studies supports a contribution of common variants to susceptibility to common disease. Nat. Genet. *33*, 177–182. https://doi.org/10.1038/ng1071.
35. (2024). Genomic data in the All of Us Research Program. Nature *627*, 340–346. https://doi.org/10.1038/s41586-023-06957-x.
36. Mitchell, T.J., and Beauchamp, J.J. (1988). Bayesian Variable Selection in Linear Regression. J. Am. Stat. Assoc. *83*, 1023–1032. https://doi.org/10.1080/01621459.1988.10478694.
37. Privé, F., Arbel, J., and Vilhjálmsson, B.J. (2020). LDpred2: better, faster, stronger. Bioinformatics *36*, 5424–5431. https://doi.org/10.1093/bioinformatics/btaa1029.

The American Journal of Human Genetics, Volume *112*

Supplemental information

The contribution of coding variants to the heritability of multiple cancer types using UK Biobank whole-exome sequencing data

Naomi Wilcox, Jonathan P. Tyrer, Joe Dennis, Xin Yang, John R.B. Perry, Eugene J. Gardner, and Douglas F. Easton

Supplementary Material

Table of Contents

Supplementary Methods **... 2**

Individual cancer model ... 2
Joint cancer model, method 1 ... 5
Joint cancer model, method 2 ... 8
Maximisation ... 9
Model extension ... 9

Supplementary Tables ***... 10***

Single cancer heritability results ... 11

Joint cancer heritability results ... 18

Supplementary Figures ***... 31***

Pancreatic Cancer ... 31

Endometrial Cancer ... 33

Ovarian Cancer ... 35

Oesophagus Cancer ... 37

Kidney Cancer ... 39

Bladder Cancer ... 41

Malignant Melanoma ... 43

Supplementary Methods

Individual cancer model

To derive the likelihood function we stratify the number of carriers by sex, family history, and case/control status.

	FEMALE					MALE				
	Control		Case			Control		Case		
	FH 0	FH 1	FH 0	FH 1		FH 0	FH 1	FH 0	FH 1	
**Non-carrier**	$N_{F0} - n_{F0j}$	$N_{F1} - n_{F1}$	$N_{F2} - n_{F2j}$	$N_{F3} - n_{F3j}$	$N_F - n_F$	$N_{M0} - n_{M0j}$	$N_{M1} - n_{M1j}$	N_{M2}— [illegible]	$N_{M3} - n_{M3j}$	$N_M - n_M$
**Carrier**	n_{F0j}	n_{F1j}	n_{F2j}	n_{F3j}	$n_{F0j} + n_{F1j} + n_{F2}$ [illegible] $+ n_{F3j} = n_F$	n_{M0j}	n_{M1j}	n_{M2j}	n_{M3j}	$n_{M0j} + n_{M1j} + n_{M2}$ [illegible] $+ n_{M3j} = n_M$
	N_{F0}	N_{F1}	N_{F2}	N_{F3}	$N_{F0} + N_{F1} + N_{F2} + N_{F3} = N_F$	N_{M0}	N_{M1}	N_{M2}	N_{M3}	$N_{M0} + N_{M1} + N_{M2} + N_{M3} = N_M$

For each sex, the number of carriers in each stratum can be modelled by a multinomial distribution with a probability mass function:

$$P(n_{oj}, n_{1j}, n_{2j}, n_{3j} | n_j, \beta_j) = \frac{\prod_{k=0}^{3}\left(N_k \frac{1}{2}\left(ke^{\beta_j}+2-k\right)\right)^{n_{kj}}}{\left(\sum_{k=0}^{3} N_k \frac{1}{2}\left(ke^{\beta_j}+2-k\right)\right)^{n_j}}$$

Defining $\gamma_{Fk} = log(\frac{N_{Fk}}{N_{F0}})$, $\gamma_{Mk} = log(\frac{N_{Mk}}{N_{M0}})$, and multiplying the probabilities for males and females, this simplifies to:

$$P\left(n_{oj}, n_{1j}, n_{2j}, n_{3j} \middle| n_j, \beta_j\right) = C \frac{\prod_{k=0}^{3}\left(\frac{1}{2}\left(ke^{\beta_j} + 2 - k\right)\right)^{n_{Fkj}} \prod_{k=0}^{3}\left(\frac{1}{2}\left(ke^{\beta_j} + 2 - k\right)\right)^{n_{Mkj}}}{\left(\sum_{k=0}^{3} e^{\gamma_{F_k}} \frac{1}{2}\left(ke^{\beta_j} + 2 - k\right)\right)^{n_{Fj}} \left(\sum_{k=0}^{3} e^{\gamma_{M_k}} \frac{1}{2}\left(ke^{\beta_j} + 2 - k\right)\right)^{n_{Mj}}}$$

Where $C = \binom{n_{Fj}}{n_{F0j} \quad n_{F1j} \quad n_{F2j} \quad n_{F3j}} \binom{n_{Mj}}{n_{M0j} \quad n_{M1j} \quad n_{M2j} \quad n_{M3j}} e^{\sum_{k=0}^{3}\gamma_{Fk} n_{Fkj} + \sum_{k=0}^{3}\gamma_{Mk} n_{Mkj}}$ is independent of the prior distribution.

The likelihood is integrated over the prior distribution to give the likelihood to be maximised:

$$L(\alpha, \eta) \propto \prod_{j=1}^{J} \int \frac{\prod_{k=0}^{3}\left(\frac{1}{2}\left(ke^{\beta_j} + 2 - k\right)\right)^{n_{Fkj}} \prod_{k=0}^{3}\left(\frac{1}{2}\left(ke^{\beta_j} + 2 - k\right)\right)^{n_{Mkj}}}{\left(\sum_{k=0}^{3} e^{\gamma_{F_k}} \frac{1}{2}\left(ke^{\beta_j} + 2 - k\right)\right)^{n_{Fj}} \left(\sum_{k=0}^{3} e^{\gamma_{M_k}} \frac{1}{2}\left(ke^{\beta_j} + 2 - k\right)\right)^{n_{Mj}}} f\left(\beta_j \middle| \alpha, \eta\right) d\beta_j$$

Where $f(\beta_j|\alpha, \eta)$ is the prior distribution on β_j.

Writing $L_j\left(\beta_j\right) =$

$$\left(\sum_{k=0}^{3} e^{\gamma_{F_k}}\right)^{n_{Fj}} \left(\sum_{k=0}^{3} e^{\gamma_{M_k}}\right)^{n_{Mj}} \frac{\prod_{k=0}^{3}\left(\frac{1}{2}\left(ke^{\beta_j}+2-k\right)\right)^{n_{Fkj}} \prod_{k=0}^{3}\left(\frac{1}{2}(ke^{\beta_j}+2-k)\right)^{n_{Mkj}}}{\left(\sum_{k=0}^{3} e^{\gamma_{F_k}} \frac{1}{2}\left(\left(ke^{\beta_j}+2-k\right)\right)\right)^{n_{Fj}} \left(\sum_{k=0}^{3} e^{\gamma_{M_k}} \frac{1}{2}(ke^{\beta_j}+2-k)\right)^{n_{Mj}}}$$

$L(\alpha, \eta) \propto \prod_{j=1}^{J} \int L_j\left(\beta_j\right) f\left(\beta_j \middle| \alpha, \eta\right) d\beta_j = \prod_{j=1}^{J}\left(1 - \alpha + \alpha \int L_j\left(\beta_j\right) g\left(\beta_j \middle| \eta\right) d\beta_j\right) = \prod_{j=1}^{J}\left(1 - \alpha + \alpha L_{*j}\right)$.

The posterior probability a gene is associated, given optimised estimates of $\alpha$and η is: $P\left(\beta_j \middle| Data\right) = \frac{\alpha \int L_j(\beta_j) g\left(\beta_j|\eta\right) d\beta_j}{1-\alpha+\alpha \int L_j(\beta_j) g\left(\beta_j|\eta\right) d\beta_j} = \frac{\alpha L_{*j}}{1-\alpha+\alpha L_{*j}}$.

The posterior mean β_j is: $\frac{\int \beta_j L_j(\beta_j) g\left(\beta_j|\eta\right) d\beta_j}{L_{*j}}$.

And the posterior mean relative risk e^{β_j} is: $\frac{\int e^{\beta_j} L_j(\beta_j) g\left(\beta_j|\eta\right) d\beta_j}{L_{*j}}$.

For gene j with aggregate PTV frequency, p_j, associated with relative risk e^{β_j}, the FRR is $\lambda_j = 1 + \frac{p_j(e^{\beta_j}-1)^2}{(2p_j(e^{\beta_j}-1)+1)^2}$

Using control and case data, we estimate the allele frequency based on the posterior distribution of the relative risk: $p_{Bj}(\beta_j) = \frac{n_{F0j}+n_{F1j}+n_{F2j}+n_{F3j}}{2(N_{F0}+N_{F1}+e^{\beta_j}(N_{F2}+N_{F3})}$.

Hence $\lambda_{jB} = 1 + \frac{\alpha}{1-\alpha+\alpha L_{*j}} \int L_j(\beta_j) g(\beta_j|\eta) \frac{p_{jB}(\beta_j)(e^{p_{jB}(\beta_j)}-1)^2}{(2p_{jB}(\beta_j)\left(e^{p_{jB}(\beta_j)}-1\right)+1)^2} d\beta_j$

The total FRR over all genes, assuming an additive model, is given by: $\hat{\lambda}_{TOT} = 1 + \sum_{j=1}^{J}(\lambda_j - 1)$.

Assuming that the PTVs combine multiplicatively with other genetic or familial factors, and an overall FRR of 2, the percentage contribution of each gene to the overall FRR is: $100 \times \frac{log(\hat{\lambda}_j)}{log\ (2)}$ and the total contribution of PTVs in all genes is: $100 \times \frac{log(\hat{\lambda}_{TOT})}{log\ (2)}$.

These equations simplify if there is no recorded family history information. In this case, the multinomial distribution simplifies to a binomial distribution. We set $N_{F1} = N_{F3} = N_{M1} = N_{M3} = 0$. Therefore for each sex: $P(n_{oj}, n_{2j}|n_j, \beta_j) \propto \frac{(N_0)^{n_{0j}}\left(N_2 e^{\beta_j}\right)^{n_{2j}}}{\left(N_0+N_2 e^{\beta_j}\right)^{n_j}}$

Defining $\gamma_{F2} = log(\frac{N_{F2}}{N_{F0}})$, $\gamma_{M2} = log(\frac{N_{M2}}{N_{M0}})$, as above, and multiplying the probabilities for males and females, this simplifies to:

$P(n_{oj}, n_{2j}|n_j, \beta_j) = C \frac{\left(e^{\gamma_{F2}} e^{\beta_j}\right)^{n_{F2j}}\left(e^{\gamma_{M2}} e^{\beta_j}\right)^{n_{M2j}}}{\left(1+e^{\gamma_{F2}} e^{\beta_j}\right)^{n_{Fj}}\left(1+e^{\gamma_{M2}} e^{\beta_j}\right)^{n_{Mj}}}$. Here C=$\binom{n_{Fj}}{n_{F2j}}\binom{n_{Mj}}{n_{M2j}}$.

Therefore, $L(\alpha,\eta) \propto \prod_{j=1}^{J} \int \frac{(N_{F0})^{n_{F0j}}\left(N_{F2}e^{\beta_j}\right)^{n_{F2j}}(N_{M0})^{n_{M0j}}\left(N_{M2}e^{\beta_j}\right)^{n_{M2j}}}{\left(1+e^{\gamma_{F2}}e^{\beta_j}\right)^{n_{Fj}}\left(1+e^{\gamma_{M2}}e^{\beta_j}\right)^{n_{Mj}}} f\left(\beta_j|\alpha,\eta\right) d\beta_j$

Joint cancer model, method 1

We now consider two cancers and assume distributions for effect sizes in which a proportion, α_1, of genes are associated with cancer 1, and α_2 are associated with cancer 2. The log-relative risk for cancer 1, β_1, has a density of the form $f(\beta_1|\alpha_1,\eta_1)$ and the log-relative risk for cancer 2, β_2, has a density of the form $f(\beta_2|\alpha_2,\eta_2)$. We assume that the prior probabilities can be correlated (i.e., the probability that a gene is a risk for cancer 2 is dependent on whether it is a gene for cancer 1).

There are now 4 combinations for a gene j:

		$\boldsymbol{Cancer\ 1, \beta_{1j}}$	
		**0 (not associated)**<br>$w.p\ (\mathbf{1}-\boldsymbol{\alpha_1})$	$\boldsymbol{g(\beta_1|\eta)}$ **(associated)**<br>$w.p\ \boldsymbol{\alpha_1}$
$\boldsymbol{Cancer\ 2, \beta_{2j}}$	**0 (not associated)**<br>$w.p\ (\mathbf{1}-\boldsymbol{\alpha_2})$	(0,0)<br>$w.p\ (1-\alpha_{10}-\alpha_{01}-\alpha_{11})$	$(g(\beta_1|\eta_1),0)$<br>$w.p\ \alpha_{10}$
	$\boldsymbol{g(\beta_2|\eta_2)}$ **(associated)**<br>$w.p\ \boldsymbol{\alpha_2}$	$(0, g(\beta_2|\eta_2))$<br>$w.p\ \alpha_{01}$	$(g(\beta_1|\eta_1), g(\beta_2|\eta_2))$<br>$w.p\ \alpha_{11}$

i.e.

$$(\beta_1,\beta_2)\sim\begin{cases}(0,0)\ w.p.(1-\alpha_{10}-\alpha_{01}-\alpha_{11})\\ (g(\beta_1|\eta_1),0)\ w.p.\alpha_{10}\\ (0,g(\beta_2|\eta_2))\ w.p\ \alpha_{01}\\ (g(\beta_1|\eta_1),g(\beta_2|\eta_2))\ w.p.\alpha_{11}\end{cases}$$

Where $g(\beta_1|\eta_1)\sim\eta_1\exp(-\eta_1\beta_1)$, $g(\beta_2|\eta_2)\sim\eta_2\exp(-\eta_2\beta_2)$,

For simplicity, we assume that the effect sizes β_1 and β_2 are uncorrelated.

There are five parameters to estimate: α_{10}, α_{01}, α_{11}, η_1, and η_2.

We can calculate $\alpha_1 = \alpha_{10} + \alpha_{11}$ and $\alpha_2 = \alpha_{01} + \alpha_{11}$. We note $\alpha_1, \alpha_2, \eta_1$, and $\eta_2$calculated here may differ to the values from optimising the individual cancer models.

The odds ratio $\alpha_{11}(1-\alpha_{10}-\alpha_{01}-\alpha_{11})/(\alpha_{10}\alpha_{01})$ represents the degree of enrichment of susceptibility to cancer 2 given cancer 1 (or vice versa).

The likelihood can be written as:

$$L(\alpha_{10},\alpha_{01},\alpha_{11},\eta_1,\eta_2) \propto \prod_{j=1}^{J} \iint L_j(\beta_{1j},\beta_{2j}) f(\beta_{1j},\beta_{2j}|\alpha_{10},\alpha_{01},\alpha_{11},\eta_1,\eta_2)\, d\beta_{1j} d\beta_{2j}$$

$$= \prod_{j=1}^{J} \int L_j(\beta_{1j}) f(\beta_{1j}|\alpha_1\eta) d\beta_{1j} \int L_j(\beta_{2j}) f(\beta_{2j}|\alpha_2\eta) d\beta_{2j}$$

$$= \prod_{j=1}^{J} \Big((1-\alpha_{10}-\alpha_{01}-\alpha_{11}) + \alpha_{10} \int L_{j1}(\beta_{1j}) g(\beta_{1j}|\eta_1) d\beta_{1j}$$

$$+ \alpha_{01} \int L_{j2}(\beta_{2j}) g(\beta_{2j}|\eta_2) d\beta_{1j}$$

$$+ \alpha_{11} \int L_j(\beta_{1j}) g(\beta_{1j}|\eta_1) d\beta_{1j} \int L_j(\beta_{2j}) g(\beta_{2j}|\eta_2) d\beta_{2j} \Big) =$$

$$= \prod_{j=1}^{J} \big((1-\alpha_{10}-\alpha_{01}-\alpha_{11}) + \alpha_{10} L_{*j1}(\eta_1) + \alpha_{01} L_{*j2}(\eta_2)$$

$$+ \alpha_{11} L_{*j1}(\eta_1) L_{*j2}(\eta_2) \big)$$

The posterior probability a gene is associated with cancer 1 given the estimates α_{10}, α_{01}, α_{11}, $\eta_1$and η_2, is: $\mathrm{P(Risk\ gene\ for\ cancer\ 1|Data)} = \frac{\alpha_{10}L_{1*j}+\alpha_{11}L_{1*j}L_{2*j}}{(1-\alpha_{10}-\alpha_{01}-\alpha_{11})+\alpha_{10}L_{1*j}+\alpha_{01}L_{2*j}+\alpha_{11}L_{1*j}L_{2*j}}$, and similarly for cancer 2. L_{1*} and L_{2*} both depend on $\eta_1$and η_2 respectively.

The posterior probability a gene is associated with both cancers is:

$$\mathrm{P}(\text{Risk gene for both cancers } | \text{Data}) = \frac{\alpha_{11} \mathrm{L}_{1*\mathrm{j}} \mathrm{L}_{2*\mathrm{j}}}{(1 - \alpha_{10} - \alpha_{01} - \alpha_{11}) + \alpha_{10} \mathrm{L}_{1*\mathrm{j}} + \alpha_{01} \mathrm{L}_{2*\mathrm{j}} + \alpha_{11} \mathrm{L}_{1*\mathrm{j}} \mathrm{L}_{2*\mathrm{j}}}$$

We can calculate a likelihood ratio test to test for enrichment of susceptibility to cancer 2 given cancer 1 (or vice versa), by comparing the joint likelihood above to the joint likelihood when the cancers are independent.

		$\boldsymbol{Cancer\ 1, \beta_{1j}}$	
		**0 (not associated)** $w.p\ (\mathbf{1} - \boldsymbol{\alpha_1})$	$\boldsymbol{g(\beta_1 \mid \eta_1)}$ **(associated)** $w.p\ \boldsymbol{\alpha_1}$
$Cancer\ 2, \beta_{2j}$	**0 (not associated)** $w.p\ (\mathbf{1} - \boldsymbol{\alpha_2})$	(0,0) $w.p\ (1 - \alpha_{10} - \alpha_{01} - \alpha_{11}) = (1 - \alpha_1)\ (1 - \alpha_2)$	$(g(\beta_1 \mid \eta_1), 0)$ $w.p\ \alpha_{10} = \alpha_1 (1 - \alpha_2)$
	$\boldsymbol{g(\beta_2 \mid \eta_2)}$ **(associated)** $w.p\ \boldsymbol{\alpha_2}$	$(0, g(\beta_2 \mid \eta_2))$ $w.p\ \alpha_{01} = \alpha_2 (1 - \alpha_1)$	$(g(\beta_1 \mid \eta_1), g(\beta_2 \mid \eta_2))$ $w.p\ \alpha_{11} = \alpha_1 \alpha_2$

The odds ratio $\frac{\alpha_{11}(1-\alpha_{10}-\alpha_{01}-\alpha_{11})}{\alpha_{10}\alpha_{01}} = \frac{\alpha_1\alpha_2(1-\alpha_1)\ (1-\alpha_2)}{\alpha_1\alpha_2(1-\alpha_1)\ (1-\alpha_2)} = 1$, and $\alpha_{11} = \alpha_1\alpha_2$. Therefore:

$$L(\alpha_{10}, \alpha_{01}, \alpha_{11}, \eta_1, \eta_2) = \prod_{j=1}^{J} \left(\left(\frac{\alpha_{01}\alpha_{10}}{\alpha_{11}} \right) + \alpha_{10} L_{*j1}(\eta_1) + \alpha_{01} L_{*j2}(\eta_2) + \alpha_{11} L_{*j1}(\eta_1) L_{*j2}(\eta_2) \right)$$

$$= \prod_{j=1}^{J} \frac{1}{\alpha_{11}} \left((\alpha_{01}\alpha_{10}) + \alpha_{11}\alpha_{10} L_{*j1}(\eta_1) + \alpha_{11}\alpha_{01} L_{*j2}(\eta_2) + {\alpha_{11}}^2 L_{*j1}(\eta_1) L_{*j2}(\eta_2) \right)$$

$$= \prod_{j=1}^{J} \frac{1}{\alpha_{11}} \left(\alpha_{01} + \alpha_{11} L_{*j1}(\eta_1) \right) \left(\alpha_{10} + \alpha_{11} L_{*j2}(\eta_2) \right)$$

$$= \prod_{j=1}^{J} \frac{1}{\alpha_1\alpha_2} \left(\alpha_2(1 - \alpha_1) + \alpha_1\alpha_2 L_{*j1}(\eta_1) \right) \left(\alpha_1(1 - \alpha_2) + \alpha_1\alpha_2 L_{*j2}(\eta_2) \right) =$$

$$= \prod_{j=1}^{J} (1 - \alpha_1 + \alpha_1 L_{*j1}(\eta_1))(1 - \alpha_2 + \alpha_2 L_{*j1}(\eta_2))$$

i.e., the likelihood simplifies to the product of the individual cancer likelihoods, assuming consistent $\eta_1$and η_2. Therefore, the likelihood ratio test is a comparison of the joint log-likelihood to the sum of the log-likelihoods of the separate models, using the chi-square distribution with 1 degree of freedom (df).

Joint cancer model, method 2

For some cancer pairs method 1 is difficult to optimise and we therefore consider an alternative method where we fix the marginal parameters α_1 and α_2 and estimate $\alpha_3 = P(C_2|C_1)$, η_1 and η_2. We then calculate $\alpha_{01}, \alpha_{10}, \alpha_{11}$ and α_{00}:

$$\alpha_{11} = P(C_1 \cap\ C_2) = P(C_2|C_1)P(C_1) = P(C_1|C_2)P(C_2) = \alpha_1\alpha_3$$

$$\alpha_{01} = P(\sim C_1 \cap\ C_2) = P(C_2|\sim C_1)P(\sim C_1) = P(\sim C_1|C_2)P(C_2) = \alpha_2\left(1 - \frac{\alpha_1\alpha_3}{\alpha_2}\right) = \alpha_2 - \alpha_1\alpha_3$$

$$\alpha_{10} = P(C_1 \cap\ \sim C_2) = \alpha_{11} = P(\sim C_2|C_1)P(C_1) = P(C_1|\sim C_2)P(\sim C_2) = \alpha_1(1 - \alpha_3)$$

$$\alpha_{00} = P(\sim C_1 \cap\ \sim C_2) = 1 - \alpha_1 - \alpha_2 + \alpha_1\alpha_3$$

These values should be similar to the values from joint model method 1 if the values of η_1, η_2, α_1 and α_2 are similar.

We set $P(C_2|C_1)$= α_3, and can calculate $P(C_1|C_2) = \frac{\alpha_1\alpha_3}{\alpha_2}$

The equivalent likelihood ratio test is testing $\alpha_3 = \alpha_2$, i.e., $P(C_2|C_1) = P(C_2)$.

We note that $\alpha_1\alpha_3 \leq \alpha_1$ and $\alpha_1\alpha_3 \leq \alpha_2$, i.e., $\alpha_3 \leq min\left(\frac{\alpha_2}{\alpha_1}, 1\right)$.

It therefore makes sense to fit the model with cancer 1 being the cancer with the greatest proportion of genes to be risk associated so that the upper bound of α_3 can consistently be set at 1. If not, and α_3 is estimated to be $\geq \frac{\alpha_2}{\alpha_1}$ then $P(C_1|C_2)$.

This method was used for bowel-endometrial and lung-ovarian cancer.

Maximisation

We maximise each log-likelihood to estimate the parameters using the L-BFGS-B optimisation algorithm in the optim package on R. This is a method by Byrd et. Al, 1995 for large non-linear optimisation problems which allows for multivariate estimation and parameter box constraints, i.e., $0 < \alpha < 1$[25].

Model extension

The joint model method 1 could be extended to more than 2 cancers e.g., breast, prostate and ovarian cancer which have significant overlap. For 3 cancers we would need to estimate 7 α's and 3 η's:

$$L(\alpha_{100}, \alpha_{010}, \alpha_{001}, \alpha_{110}, \alpha_{101}, \alpha_{011}, \alpha_{111}, \eta_1, \eta_2, \eta_3)$$

$$\propto \prod_{j=1}^{J} \Big((1 - \alpha_{100} - \alpha_{010} - \alpha_{001} - \alpha_{110} - \alpha_{101} - \alpha_{011} - \alpha_{111}) + \alpha_{100} L_{*j1}(\eta_1)$$

$$+ \alpha_{010} L_{*j2}(\eta_2) + \alpha_{001} L_{*j3}(\eta_3) + \alpha_{110} L_{*j1}(\eta_1) L_{*j2}(\eta_2) + \alpha_{101} L_{*j1}(\eta_1) L_{*j3}(\eta_2)$$

$$+ \alpha_{011} L_{*j2}(\eta_1) L_{*j3}(\eta_2) + \alpha_{111} L_{*j1}(\eta_1) L_{*j2}(\eta_2) L_{*j3}(\eta_2)\Big)$$

To account for n cancers, we would need to estimate 2^n-1 α's and n η's.

Supplementary Tables

LRT p-values

Table S8 | A comparison of Wald test, Firth regression, and LRT P-values for genes reaching exome-wide significance in the Wald test and with case carriers ≤5.

Cancer	Gene	Control		Case		OR (CI)	P-value		
		Non-carriers	carriers	Non-carriers	carriers		Wald	Firth regression	LRT
Pancreatic	*MEN1*	418224	2	1079	2	429 (57.4, 3200)	3.46E-09	4.17E-06	1.66E-05
	RCN2	418188	38	1078	3	31 (9.53, 101)	1.17E-08	2.32E-05	0.000136
	YPEL3	418215	11	1079	2	78 (17.2, 355)	1.73E-08	6.32E-05	0.000292
	SMC2	418177	49	1078	3	24.9 (7.74, 80.1)	7.03E-08	4.93E-05	0.000258
	GNG10	418205	21	1079	2	39.4 (9.15, 169)	8.05E-07	0.00022	0.00113
	ZNF461	418201	25	1079	2	32.8 (7.71, 139)	2.26E-06	0.000269	0.00164
Endometrial	*MLH1*	225410	9	1971	3	40.3 (10.9, 149)	3.24E-08	1.77E-05	8.2E-05
	ACRV1	225388	31	1970	4	14.6 (5.14, 41.4)	4.77E-07	6.21E-05	0.000225
	STK32C	225403	16	1971	3	21.5 (6.25, 74.2)	1.15E-06	0.000112	0.000466
	PSMC6	225417	2	1972	2	115 (16, 825)	2.37E-06	5.70E-05	0.000239
Ovarian	*IVD*	225957	64	1367	5	12.9 (5.19, 32.2)	3.84E-08	1.66E-05	6.26E-05
	JAML	226000	21	1369	3	24 (7.15, 80.7)	2.75E-07	6.72E-05	0.000322
	KCNAB2	226016	5	1370	2	68.9 (13.2, 360)	5.17E-07	8.77E-05	0.000461
	ZFP14	226016	5	1370	2	64.8 (12.5, 337)	6.92E-07	8.43E-05	0.000521
	TMEM163	226015	6	1370	2	54.1 (10.9, 269)	1.09E-06	1.20E-04	0.000716
	TMEM167A	226014	7	1370	2	47.2 (9.76, 228)	1.64E-06	1.48E-04	0.000916
	NHEJ1	225993	28	1369	3	17.7 (5.36, 58.2)	2.37E-06	1.76E-04	0.000782
Oesophagus	*KNL1*	418238	67	998	4	23.7 (8.54, 65.6)	1.17E-09	6.14E-06	3.09E-05
	IRF2BP2	418282	23	1000	2	36 (8.31, 156)	1.66E-06	0.000220	0.00138
Kidney	*FGL2*	417654	12	1639	2	49.3 (10.8, 225)	4.66E-07	0.000110	0.000768
	TTC9	417627	39	1638	3	20.4 (6.25, 66.5)	5.78E-07	0.000107	0.000493
	EXOC7	417536	130	1636	5	9.53 (3.88, 23.4)	8.45E-07	7.80E-05	0.000251
	NCK2	417652	14	1639	2	39.8 (8.94, 178)	1.34E-06	0.000188	0.00117
	TMEM174	417653	13	1639	2	39.9 (8.86, 180)	1.60E-06	0.000198	0.00118
Bladder	*DLX2*	417873	9	1423	2	99.5 (19.9, 498)	2.13E-08	3.30E-05	0.00205
	ZNF506	417873	9	1423	2	60.6 (12.4, 296)	3.98E-07	9.10E-05	0.000555
	CDCP2	417714	168	1420	5	9.81 (4, 24.1)	6.25E-07	6.72E-05	0.000220
	TMEM222	417836	46	1422	3	19.5 (5.95, 63.6)	9.11E-07	0.000129	0.000572
	KDM1A	417833	49	1422	3	18.3 (5.62, 59.7)	1.41E-06	0.000156	0.000681
	ARHGEF6	227079	2	311	1	400 (35, 4570)	1.43E-06	0.000176	0.00203
	HR	417711	171	1420	5	9.01 (3.68, 22.1)	1.53E-06	0.000104	0.000326
	NLRP10	417879	3	1424	1	293 (28.9, 2970)	1.54E-06	0.000398	0.00269
Malignant Melanoma	*MED9*	415117	11	4175	4	36.1 (11.5, 114)	8.62E-10	2.16E-06	9.20E-06
	MRPL44	415083	45	4174	5	11.6 (4.59, 29.3)	2.17E-07	3.46E-05	0.000113
	CDKN2A	415115	13	4176	3	23 (6.53, 80.8)	1.04E-06	9.56E-05	0.000413
	KLHL32	415058	70	4174	5	7.14 (2.88, 17.7)	2.24E-05	0.000371	0.00102

Single cancer heritability results

Table S9 | Heritability results for breast cancer, including the posterior probability of being disease associated, posterior mean effect sizes and the proportion of the familial relative risk (FRR) explained. Genes listed have posterior probability >0.1. This analysis uses females and males, incorporating family history data, and adjusts for CNV frequency. Results are sorted by descending posterior probability.

Gene	Posterior probability	Posterior mean β	Posterior mean e^{β}	λ	%FRR
BRCA1	1.000	2.10	8.19	1.02084	2.976
BRCA2	1.000	1.70	5.51	1.02525	3.598
CHEK2	1.000	0.85	2.35	1.00518	0.746
PALB2	1.000	1.34	3.81	1.00632	0.910
ATM	1.000	0.78	2.18	1.00176	0.254
MAP3K1	0.983	1.45	4.44	1.00076	0.109
BAP1	0.280	1.22	3.61	1.00011	0.015
PCDHGB3	0.272	0.37	1.46	1.00007	0.010
COL12A1	0.188	0.76	2.20	1.00005	0.008
RNF112	0.177	0.84	2.41	1.00005	0.007
BARD1	0.144	0.65	1.96	1.00007	0.010
KLK4	0.118	0.66	1.97	1.00003	0.004
CYBC1	0.116	1.92	8.22	1.00010	0.014
			**All genes**	**1.06300**	**8.821**

Table S10 | Heritability results for bowel cancer, including the posterior probability of being disease associated, posterior mean effect sizes and the proportion of the familial relative risk (FRR) explained. Genes listed have posterior probability >0.1. This analysis uses females and males, incorporating family history data, and adjusts for CNV frequency. Results are sorted by descending posterior probability.

Gene	Posterior probability	Posterior mean β	Posterior mean e^{β}	λ	%FRR
MSH2	1.000	1.89	6.71	1.00614	0.884
MSH6	1.000	1.71	5.57	1.00769	1.105
MLH1	1.000	2.86	17.56	1.02583	3.678
APC	1.000	2.39	11.27	1.00465	0.670
GAPDH	0.523	1.18	3.38	1.00034	0.049
FLCN	0.278	0.99	2.77	1.00021	0.030
			**All genes**	1.04750	6.697

Table S11 | Heritability results for prostate cancer, including the posterior probability of being disease associated, posterior mean effect sizes and the proportion of the familial relative risk (FRR) explained. Genes listed have posterior probability >0.1. This analysis uses females and males, incorporating family history data, and adjusts for CNV frequency. Results are sorted by descending posterior probability.

Gene	Posterior probability	Posterior mean β	Posterior mean e^{β}	λ	%FRR
BRCA2	1.000	0.72	2.05	1.00166	0.2399
CHEK2	1.000	0.44	1.56	1.00100	0.1441
ATM	0.999	0.54	1.73	1.00067	0.0970
PPP5C	0.388	0.46	1.61	1.00008	0.0109
INVS	0.338	0.39	1.50	1.00006	0.0084
BET1	0.272	0.44	1.58	1.00004	0.0060
PNLDC1	0.265	0.42	1.54	1.00004	0.0061
PPEF2	0.247	0.25	1.30	1.00004	0.0055
CHID1	0.221	0.37	1.47	1.00003	0.0041
MICB	0.219	0.32	1.39	1.00003	0.0041
MYH7	0.217	0.42	1.55	1.00003	0.0043
TRMT44	0.194	0.26	1.30	1.00003	0.0048
FSIP2	0.184	0.19	1.22	1.00004	0.0051
SNTG2	0.161	0.29	1.36	1.00002	0.0028
TMC2	0.154	0.34	1.42	1.00003	0.0050
WDR59	0.153	0.33	1.41	1.00003	0.0045
LYST	0.152	0.38	1.50	1.00002	0.0027
GEMIN2	0.148	0.66	2.11	1.00001	0.0019
MFSD8	0.135	0.49	1.71	1.00003	0.0041
C9orf50	0.128	0.53	1.81	1.00001	0.0013
NAA11	0.126	0.26	1.32	1.00001	0.0018
CCDC188	0.121	0.29	1.35	1.00001	0.0018
OAT	0.119	0.32	1.40	1.00001	0.0019
BSCL2	0.119	0.48	1.68	1.00001	0.0014
BEND5	0.118	0.39	1.52	1.00001	0.0013
PABPN1	0.117	0.34	1.44	1.00001	0.0014
MAP3K19	0.115	0.26	1.31	1.00001	0.0017
FOXR1	0.111	0.38	1.51	1.00001	0.0010
SSNA1	0.111	0.29	1.36	1.00001	0.0014
FANCM	0.107	0.17	1.19	1.00001	0.0018
OSGIN1	0.107	0.56	1.90	1.00001	0.0014
CNPY2	0.106	0.39	1.53	1.00001	0.0012
SORD	0.105	0.20	1.22	1.00001	0.0016
DPH1	0.104	0.27	1.32	1.00001	0.0013
DEPDC4	0.103	0.23	1.27	1.00001	0.0014
SPG7	0.103	0.19	1.22	1.00001	0.0015
ATP8B4	0.101	0.26	1.30	1.00001	0.0019
ADAM15	0.101	0.27	1.33	1.00001	0.0014
			**All genes**	1.00751	1.0798

Table S12 | Heritability results for lung cancer, including the posterior probability of being disease associated, posterior mean effect sizes and the proportion of the familial relative risk (FRR) explained. Genes listed have posterior probability >0.1. This analysis uses females and males, incorporating family history data, and adjusts for CNV frequency. Results are sorted by descending posterior probability.

Gene	Posterior probability	Posterior mean β	Posterior mean e^{β}	λ	%FRR
ATM	0.187	0.36	1.44	1.000055	0.00799
			**All genes**	1.000649	0.09354

Table S13 | Heritability results for pancreatic cancer, including the posterior probability of being disease associated, posterior mean effect sizes and the proportion of the familial relative risk (FRR) explained. Genes listed have posterior probability >0.1. This analysis uses females and males and adjusts for CNV frequency. Results are sorted by descending posterior probability.

Gene	Posterior probability	Posterior mean β	Posterior mean e^{β}	λ	%FRR
ATM	1.000	1.608	5.188	1.024699086	3.520
SEC14L3	0.491	1.292	4.073	1.003474889	0.500
LTV1	0.377	0.915	2.662	1.001748643	0.252
MROH6	0.239	0.887	2.631	1.000861016	0.124
MAN2A2	0.152	0.607	1.916	1.000408525	0.059
ALDH1L1	0.134	0.924	2.878	1.000642404	0.093
BRCA2	0.127	0.653	2.035	1.000302181	0.044
PCNT	0.121	0.725	2.232	1.000320685	0.046
RCN2	0.117	1.264	4.838	1.000593978	0.086
PKHD1	0.113	0.659	2.060	1.000256601	0.037
CYP20A1	0.111	0.965	3.131	1.000596335	0.086
SMC2	0.109	1.202	4.499	1.000741102	0.107
CEACAM4	0.108	0.950	3.072	1.000499133	0.072
SMTNL1	0.107	0.819	2.550	1.000219483	0.032
			**All genes**	1.0762	32.930

Table S14 | Heritability results for oesophagus cancer, including the posterior probability of being disease associated, posterior mean effect sizes and the proportion of the familial relative risk (FRR) explained. Genes listed have posterior probability >0.1. This analysis uses females and males and adjusts for CNV frequency. Results are sorted by descending posterior probability.

Gene	Posterior probability	Posterior mean β	Posterior mean e^{β}	λ	%FRR
NLRP12	0.599	0.89	2.57	1.00310	0.446
ATM	0.543	0.95	2.79	1.00302	0.435
ZGRF1	0.267	0.65	2.03	1.00078	0.113
KNL1	0.213	1.19	4.35	1.00051	0.074
CAMKMT	0.178	0.85	2.72	1.00053	0.077
CFTR	0.159	0.57	1.87	1.00031	0.045
FGF11	0.151	0.76	2.43	1.00024	0.035
PCDHA8	0.126	0.45	1.62	1.00020	0.029
VPS13A	0.122	0.60	1.97	1.00018	0.026
RTN4IP1	0.121	0.75	2.48	1.00016	0.024
TMPRSS11D	0.120	0.59	1.96	1.00016	0.022
METTL24	0.114	0.58	1.92	1.00015	0.022
DTHD1	0.111	0.70	2.31	1.00011	0.017
DFFA	0.110	0.70	2.31	1.00019	0.028
CTSF	0.106	0.68	2.24	1.00011	0.016
DIS3	0.103	0.54	1.84	1.00013	0.019
			**All genes**	1.04367	6.166

Table S15 | Heritability results for endometrial cancer, including the posterior probability of being disease associated, posterior mean effect sizes and the proportion of the familial relative risk (FRR) explained. Genes listed have posterior probability >0.1. This analysis uses females only and adjusts for CNV frequency. Results are sorted by descending posterior probability.

Gene	Posterior probability	Posterior mean β	Posterior mean e^{β}	λ	%FRR
MSH6	1.000	2.94	19.01	1.1070	14.666
			**All genes**	1.1214	16.526

Joint cancer heritability results

Table S17 | Optimisation results for cancer pairs. This includes the method used (method 1 or method 2 in methods), and optimised values of alpha10=P(C1 n C2'), alpha01=P(C1' n C2), alpha11=P(C1 n C2), eta1 and eta2, as well as the p-value from the likelihood ratio test comparing this model to the model where the cancers are independent.

Cancer 1	Cancer 2	Method	alpha10	alpha01	alpha11	eta1	eta2	LRT
**Breast**	**Prostate**	1	0.00000	0.00000	0.00220	1.49	2.51	1.53E-09
**Breast**	**Ovarian**	1	0.00000	0.00000	0.00270	1.77	1.23	2.12E-08
**Bowel**	**Endom**	2	0.00020	0.00000	0.00140	1.13	0.93	3.01E-08
**Breast**	**Pancreas**	1	0.00000	0.00000	0.00230	1.62	1.57	0.0000230
**Prostate**	**Ovarian**	1	0.00000	0.00340	0.01700	6.04	2.06	0.000151
**Prostate**	**Pancreas**	1	0.00000	0.00000	0.00840	4.56	2.17	0.000277
**Breast**	**Lung**	1	0.00013	0.00026	0.00150	1.52	6.00	0.00144
**Breast**	**Bowel**	1	0.00053	0.00027	0.00160	1.51	1.00	0.00351
**Pancreas**	**Ovarian**	1	0.00000	0.00000	0.03000	3.09	2.25	0.00540
**Breast**	**Oesophagus**	1	0.00000	0.00980	0.00240	1.64	2.95	0.00622
**Lung**	**Pancreas**	1	0.00000	0.00000	0.00460	5.93	2.05	0.00693
**Bowel**	**Ovarian**	1	0.00000	0.00000	0.00290	1.52	1.19	0.0102
**Prostate**	**Oesophagus**	1	0.00000	0.00000	0.01400	5.49	2.84	0.0106
**Pancreas**	**Oesophagus**	1	0.00000	0.00000	0.00750	2.28	2.37	0.0115
**Lung**	**Prostate**	1	0.00002	0.00002	0.00250	6.00	2.01	0.0161
**Endom**	**Ovarian**	1	0.00000	0.00000	0.00390	1.51	1.28	0.0213
**Bowel**	**Pancreas**	1	0.00000	0.00000	0.00220	1.40	1.68	0.0288
**Lung**	**Oesophagus**	1	0.00000	0.00000	0.00590	5.85	2.28	0.0339
**Bowel**	**Lung**	1	0.00000	0.00000	0.00260	1.49	6.00	0.0602
**Bowel**	**Prostate**	1	0.00000	0.00000	0.00340	1.83	2.93	0.112
**Oesophagus**	**Ovarian**	1	0.00000	0.01700	0.02600	3.36	2.51	0.311
**Lung**	**Endom**	1	0.00050	0.00005	0.00200	6.00	1.50	0.399
**Bowel**	**Oesophagus**	1	0.00000	0.01300	0.00230	1.46	2.94	0.460
**Prostate**	**Endom**	1	0.01300	0.00000	0.00430	5.76	1.91	0.498
**Lung**	**Ovarian**	2	0.00000	0.03350	0.00350	5.43	2.41	0.602
**Breast**	**Endom**	1	0.00098	0.00180	0.00150	1.52	1.51	0.639
**Pancreas**	**Endom**	1	0.00270	0.00000	0.00150	1.52	1.51	1.00
**Oesophagus**	**Endom**	1	0.02400	0.00000	0.00150	3.12	1.27	1.00

Table S18 |Genes with posterior>0.8 for being associated with both cancer 1 and cancer 2 for at least 1 cancer pair. The cancer pairs columns are the cancer pairs which had posterior probability >0.8 of the gene being associated with both cancer 1 and cancer 2.

Gene	Cancer pairs
APC	Breast-Bowel, Bowel-Lung, Bowel-Ovarian, Bowel-Pancreas, Bowel-Oesophagus
ATM	Breast-Bowel, Breast-Lung, Breast-Oesophagus, Breast-Ovarian, Breast-Pancreas, Breast-Prostate, Lung-Pancreas, Pancreas-Ovarian, Prostate-Ovarian, Prostate-Pancreas, Bowel-Lung, Bowel-Pancreas, Lung-Oesophagus, Lung-Prostate, Pancreas-Oesophagus, Prostate-Oesophagus, Bowel-Prostate, Oesophagus-Ovarian
BAP1	Breast-Prostate
BRCA1	Breast-Bowel, Breast-Lung, Breast-Oesophagus, Breast-Ovarian, Breast-Pancreas, Breast-Prostate, Pancreas-Ovarian, Prostate-Ovarian, Bowel-Ovarian, Endometrial-Ovarian, Breast-Endometrial
BRCA2	Breast-Lung, Breast-Oesophagus, Breast-Ovarian, Breast-Pancreas, Breast-Prostate, Pancreas-Ovarian, Prostate-Ovarian, Prostate-Pancreas, Bowel-Ovarian, Endometrial-Ovarian, Lung-Prostate, Prostate-Oesophagus, Bowel-Prostate
CHEK2	Breast-Bowel, Breast-Oesophagus, Breast-Ovarian, Breast-Pancreas, Breast-Prostate, Prostate-Ovarian, Prostate-Pancreas, Lung-Prostate, Prostate-Oesophagus, Bowel-Prostate
MAP3K1	Breast-Lung, Breast-Oesophagus, Breast-Ovarian, Breast-Pancreas, Breast-Prostate
MLH1	Bowel-Endometrial, Breast-Bowel, Bowel-Lung, Bowel-Ovarian, Bowel-Pancreas, Bowel-Oesophagus, Bowel-Prostate
MSH2	Bowel-Endometrial, Bowel-Lung, Bowel-Ovarian, Bowel-Pancreas, Bowel-Oesophagus, Bowel-Prostate
MSH6	Bowel-Endometrial, Bowel-Lung, Bowel-Ovarian, Bowel-Pancreas, Endometrial-Ovarian, Bowel-Oesophagus, Bowel-Prostate, Lung-Endometrial, Oesophagus-Endometrial, Pancreas-Endometrial, Prostate-Endometrial
PALB2	Breast-Lung, Breast-Oesophagus, Breast-Ovarian, Breast-Pancreas, Breast-Prostate

Table S19 | Posterior probabilities for the joint cancer modelling of breast and prostate cancer. The table includes the posterior probability genes are associated with just breast cancer, just prostate cancer, or both cancers. Results shown are for genes with any posterior probability>0.1 and are sorted by descending posterior probability.

Gene	P(C1 n C2')	P(C1' n C2)	P(C1 n C2)
BRCA2	0.000	0.000	1.000
BRCA1	0.000	0.000	1.000
PALB2	0.000	0.000	1.000
ATM	0.000	0.000	1.000
CHEK2	0.000	0.000	1.000
MAP3K1	0.000	0.000	0.972
BAP1	0.000	0.000	0.824
KLK4	0.000	0.000	0.320
PPP5C	0.000	0.000	0.293
OSGIN1	0.000	0.000	0.204
PNLDC1	0.000	0.000	0.156
GEMIN2	0.000	0.000	0.154
CYBC1	0.000	0.000	0.124
DEGS1	0.000	0.000	0.113
SEC62	0.000	0.000	0.107
SNX2	0.000	0.000	0.103

Table S20 | Posterior probabilities for the joint cancer modelling of breast and ovarian cancer. The table includes the posterior probability genes are associated with just breast cancer, just ovarian cancer, or both cancers. Results shown are for genes with any posterior probability>0.1 and are sorted by descending posterior probability.

Gene	P(C1 n C2')	P(C1' n C2)	P(C1 n C2)
BRCA2	0.000	0.000	1.000
BRCA1	0.000	0.000	1.000
PALB2	0.000	0.000	1.000
CHEK2	0.000	0.000	1.000
ATM	0.000	0.000	1.000
MAP3K1	0.000	0.000	0.979
BAP1	0.000	0.000	0.416
NHEJ1	0.000	0.000	0.300
RAD51D	0.000	0.000	0.282
BRIP1	0.000	0.000	0.259
SLC35E4	0.000	0.000	0.241
KLK4	0.000	0.000	0.206
VWA2	0.000	0.000	0.200
BARD1	0.000	0.000	0.139
IVD	0.000	0.000	0.139
RNF112	0.000	0.000	0.133
ZNHIT1	0.000	0.000	0.132
COL12A1	0.000	0.000	0.130
PCDHGB3	0.000	0.000	0.122
TGM7	0.000	0.000	0.117
CYBC1	0.000	0.000	0.113
PSRC1	0.000	0.000	0.110
TRMT10B	0.000	0.000	0.105
PLEKHG4	0.000	0.000	0.104

Table S21 | Posterior probabilities for the joint cancer modelling of bowel and endometrial cancer. The table includes the posterior probability genes are associated with just bowel cancer, just endometrial cancer, or both cancers. Results shown are for genes with any posterior probability>0.1 and are sorted by descending posterior probability.

Gene	P(C1 n C2')	P(C1' n C2)	P(C1 n C2)
MSH6	1.92E-42	0	1.000
MLH1	0.019	0.000	0.981
MSH2	0.020	0.000	0.980
APC	0.481	0.000	0.519
GAPDH	0.475	0.000	0.182
FLCN	0.291	0.000	0.106
RDX	0.012	0.000	0.128
NPNT	0.122	0.000	0.051
ACRV1	0.001	0.000	0.116
MPPE1	0.113	0.000	0.029

Table S22 | Posterior probabilities for the joint cancer modelling of breast and pancreatic cancer. The table includes the posterior probability genes are associated with just breast cancer, just pancreatic cancer, or both cancers. Results shown are for genes with any posterior probability>0.1 and are sorted by descending posterior probability.

Gene	**P(C1 n C2')**	**P(C1' n C2)**	**P(C1 n C2)**
BRCA2	0.000	0.000	1.000
BRCA1	0.000	0.000	1.000
PALB2	0.000	0.000	1.000
ATM	0.000	0.000	1.000
MAP3K1	0.000	0.000	0.980
BAP1	0.000	0.000	0.393
RNF112	0.000	0.000	0.130
PCDHGB3	0.000	0.000	0.146
KLK4	0.000	0.000	0.192
COL12A1	0.000	0.000	0.131
CYBC1	0.000	0.000	0.122
CHEK2	0.000	0.000	1.000
RCN2	0.000	0.000	0.144

Table S23 | Posterior probabilities for the joint cancer modelling of prostate and ovarian cancer. The table includes the posterior probability genes are associated with just prostate cancer, just ovarian cancer, or both cancers. Results shown are for genes with any posterior probability>0.1 and are sorted by descending posterior probability.

Gene	P(C1 n C2')	P(C1' n C2)	P(C1 n C2)
BRCA2	0	1.47E-12	1.000
CHEK2	0	3.49E-08	1.000
ATM	0	5.50E-06	1.000
BRCA1	0	0.194	0.806
IVD	0	0.119	0.468
FKBP6	0	0.021	0.421
ANO2	0	0.031	0.372
PPP5C	0	0.002	0.297
BET1	0	0.003	0.292
SLC35E4	0	0.102	0.263
PLEKHG4	0	0.076	0.261
ESYT1	0	0.032	0.260
REXO5	0	0.089	0.254
NEK11	0	0.029	0.250
DPH1	0	0.008	0.238
SORD	0	0.008	0.223
OIP5	0	0.018	0.214
PNLDC1	0	0.002	0.203
CHID1	0	0.003	0.201
PNLIP	0	0.032	0.198
BRIP1	0	0.038	0.195
INVS	0	0.001	0.188
PALB2	0	0.009	0.175
WDFY4	0	0.010	0.170
JAML	0	0.034	0.169
MSH6	0	0.025	0.162
MICB	0	0.002	0.161
R3HCC1L	0	0.026	0.161
TMC2	0	0.004	0.161
ADH1B	0	0.033	0.151
PPEF2	0	0.002	0.147
WDR59	0	0.003	0.147
CEACAM20	0	0.025	0.146
ELP4	0	0.050	0.146
RELT	0	0.022	0.146
TRMT44	0	0.002	0.146
C9orf50	0	0.004	0.145
UBOX5	0	0.022	0.144
ERCC3	0	0.006	0.144
MYH7	0	0.002	0.143
SSNA1	0	0.004	0.139
DOCK2	0	0.019	0.136

PABPN1	0	0.004	0.133
ITGB3	0	0.006	0.132
TRIT1	0	0.015	0.130
GEMIN2	0	0.003	0.126
TMPRSS6	0	0.007	0.118
POLE	0	0.020	0.113
FSIP2	0	0.002	0.113
BEND5	0	0.003	0.112
NHEJ1	0	0.030	0.111
IFNL1	0	0.007	0.111
IFT57	0	0.009	0.110
PIGV	0	0.006	0.109
BRD1	0	0.023	0.108
LMF2	0	0.005	0.107
MARK3	0	0.025	0.107
SNX2	0	0.005	0.106
SPG7	0	0.004	0.105
MXD3	0	0.007	0.105
ZNHIT1	0	0.027	0.104
MFSD8	0	0.003	0.103
PITPNM1	0	0.019	0.103
RAD51D	0	0.028	0.101
FAM161A	0	0.011	0.100
ZRANB3	0	0.028	0.100

Table S24 | Posterior probabilities for the joint cancer modelling of prostate and pancreatic cancer. The table includes the posterior probability genes are associated with just prostate cancer, just pancreatic cancer, or both cancers. Results shown are for genes with any posterior probability>0.1 and are sorted by descending posterior probability.

Gene	P(C1 n C2')	P(C1' n C2)	P(C1 n C2)
BRCA2	0.000	0.000	1.000
ATM	0.000	0.000	1.000
CHEK2	0.000	0.000	1.000
SEC14L3	0.000	0.000	0.745
INVS	0.000	0.000	0.481
GEMIN2	0.000	0.000	0.232
PPP5C	0.000	0.000	0.210
BET1	0.000	0.000	0.183
C9orf50	0.000	0.000	0.148
PALB2	0.000	0.000	0.147
LYST	0.000	0.000	0.145
PNLDC1	0.000	0.000	0.128
SMC2	0.000	0.000	0.114
PACSIN3	0.000	0.000	0.112
MYH7	0.000	0.000	0.110
RCN2	0.000	0.000	0.110
SAG	0.000	0.000	0.105

Table S25 | Posterior probabilities for the joint cancer modelling of breast and lung cancer. The table includes the posterior probability genes are associated with just breast cancer, just lung cancer, or both cancers. Results shown are for genes with any posterior probability>0.1 and are sorted by descending posterior probability.

Gene	**P(C1 n C2')**	**P(C1' n C2)**	**P(C1 n C2)**
ATM	0.000979	2.74E-16	0.999
BRCA2	0.00443	4.25E-150	0.996
BRCA1	0.0760	7.27E-77	0.924
MAP3K1	0.0734	5.77E-06	0.906
PALB2	0.145	1.58E-45	0.855
CHEK2	0.290	9.24E-43	0.710
BAP1	0.0160	0.000272	0.270
PCDHGB3	0.0141	0.000199	0.149
LZTR1	0.00194	0.00108	0.104

Table S26| Posterior probabilities for the joint cancer modelling of breast and bowel cancer. The table includes the posterior probability genes are associated with just breast cancer, just bowel cancer, or both cancers. Results shown are for genes with any posterior probability>0.1 and are sorted by descending posterior probability.

Gene	P(C1 n C2')	P(C1' n C2)	P(C1 n C2)
ATM	0.0158	2.62E-16	0.984
BRCA1	0.0218	7.39E-77	0.978
MLH1	4.84E-17	0.149	0.851
APC	3.67E-11	0.183	0.817
CHEK2	0.198	1.01E-42	0.802
BARD1	0.00672	0.00211	0.791
BRCA2	0.310	2.84E-150	0.690
MSH6	1.74E-31	0.346	0.654
MSH2	3.24E-20	0.346	0.654
MAP3K1	0.453	3.18E-06	0.516
PALB2	0.491	9.12E-46	0.509
GAPDH	0.000227	0.0825	0.491
BAP1	0.0610	0.000268	0.274
NPNT	0.000825	0.0162	0.188
CTTNBP2NL	0.00265	0.00390	0.134

Table S28 | Posterior probabilities for the joint cancer modelling of breast and oesophagus cancer. The table includes the posterior probability genes are associated with just breast cancer, just oesophagus cancer, or both cancers. Results shown are for genes with any posterior probability>0.1 and are sorted by descending posterior probability.

Gene	P(C1 n C2')	P(C1' n C2)	P(C1 n C2)
BRCA2	0.000	1.14E-148	1.000
BRCA1	0.000	2.21E-75	1.000
PALB2	0.000	4.74E-44	1.000
CHEK2	0.000	3.15E-41	1.000
ATM	0.000	6.57E-15	1.000
MAP3K1	0.000	0.000	0.982
NLRP12	0.000	0.399	0.043
BAP1	0.000	0.007	0.268
RNF112	0.000	0.010	0.199
PCDHGB3	0.000	0.005	0.171
KLK4	0.000	0.014	0.168
COL12A1	0.000	0.007	0.156
KNL1	0.000	0.146	0.012
BARD1	0.000	0.009	0.146
ZGRF1	0.000	0.130	0.003
CYBC1	0.000	0.009	0.126

Table S29 | Posterior probabilities for the joint cancer modelling of lung and pancreatic cancer. The table includes the posterior probability genes are associated with just lung cancer, just pancreatic cancer, or both cancers. Results shown are for genes with any posterior probability>0.1 and are sorted by descending posterior probability.

Gene	P(C1 n C2')	P(C1' n C2)	P(C1 n C2)
ATM	0.000	0.000	1.000
BRCA2	0.000	0.000	0.438
LTV1	0.000	0.000	0.163
SEC14L3	0.000	0.000	0.161
FANCM	0.000	0.000	0.106

Supplementary Figures

Pancreatic Cancer

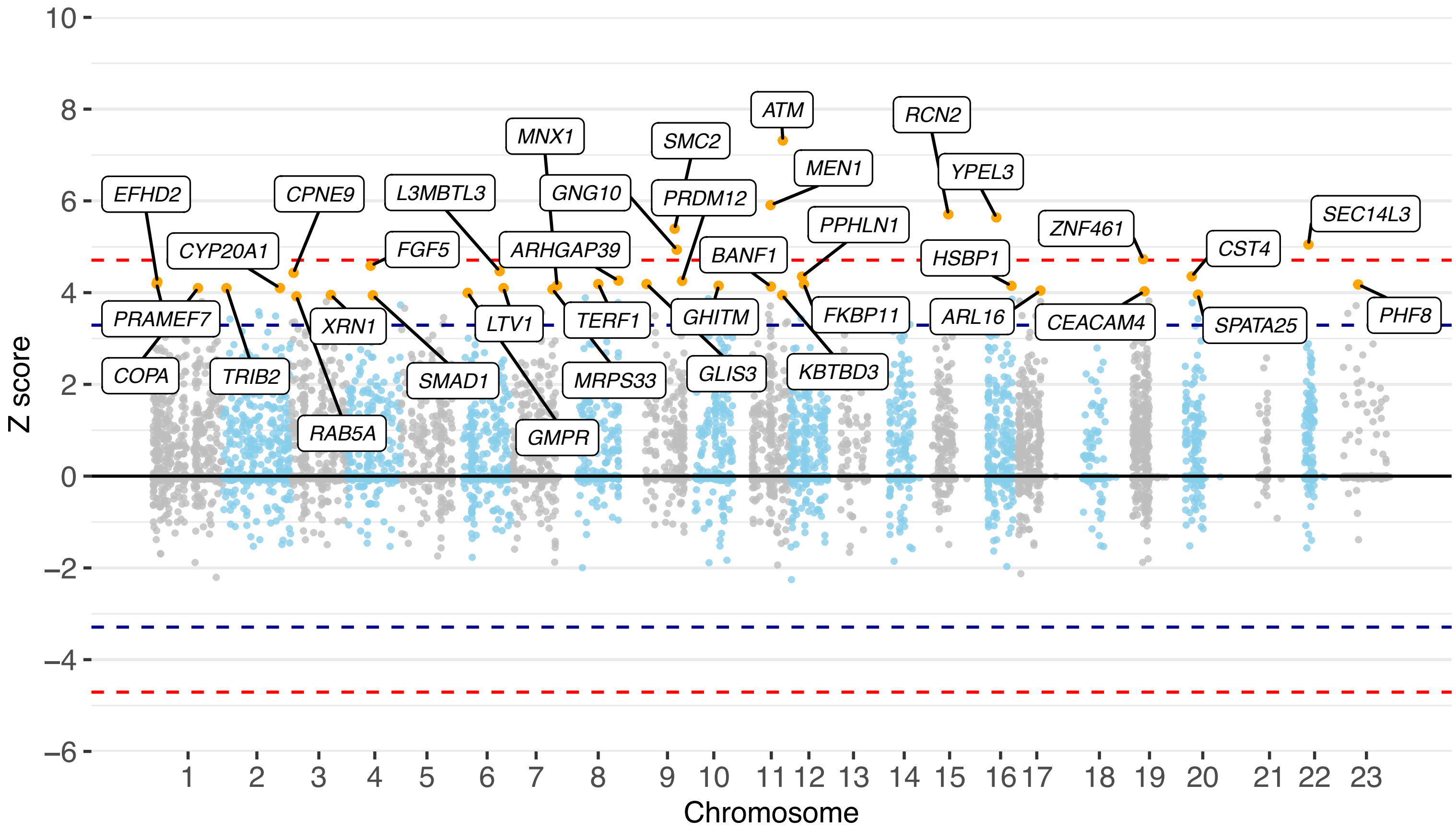

Figure S1 | Manhattan plot of z scores from assessing the association between protein-truncating variant carriers within genes and pancreatic cancer risk, using model 2. The x axis is the chromosomal position, and the y axis is the z score from testing H0: $\beta = \ln(OR) = 0$ (two-tailed) by LRT to the null model. The blue lines correspond to $z = \pm 3.29$, $P = 0.001$, the red lines correspond to $z = \pm 4.71$, $P = 2.5 \times 10{-}6$. All labelled genes are those with $P < 0.001$. All P-values are unadjusted for multiple testing.

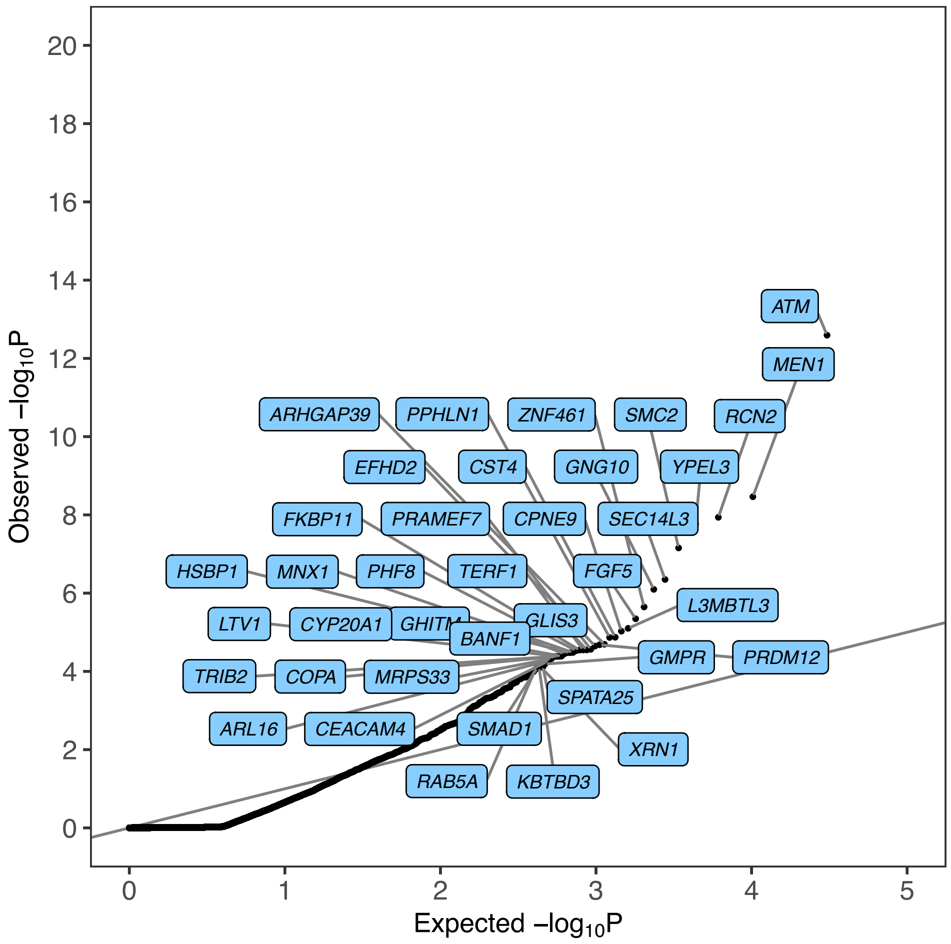

Figure S2 | Quantile–quantile plot of P values from assessing the association between protein-truncating variant carriers and pancreatic cancer risk. P-values are from testing H0: β = ln(OR) = 0 by LRT to the null model (two-tailed). The x-axis is the expected log10 P values from the null hypothesis, the y-axis is the observed log10 P value. Highlighted genes have P < 0.0001. Highlighted genes in blue are associated with an increased risk of pancreatic cancer and highlighted genes in cream are associated with decreased risk of pancreatic cancer. All P-values are unadjusted for multiple testing.

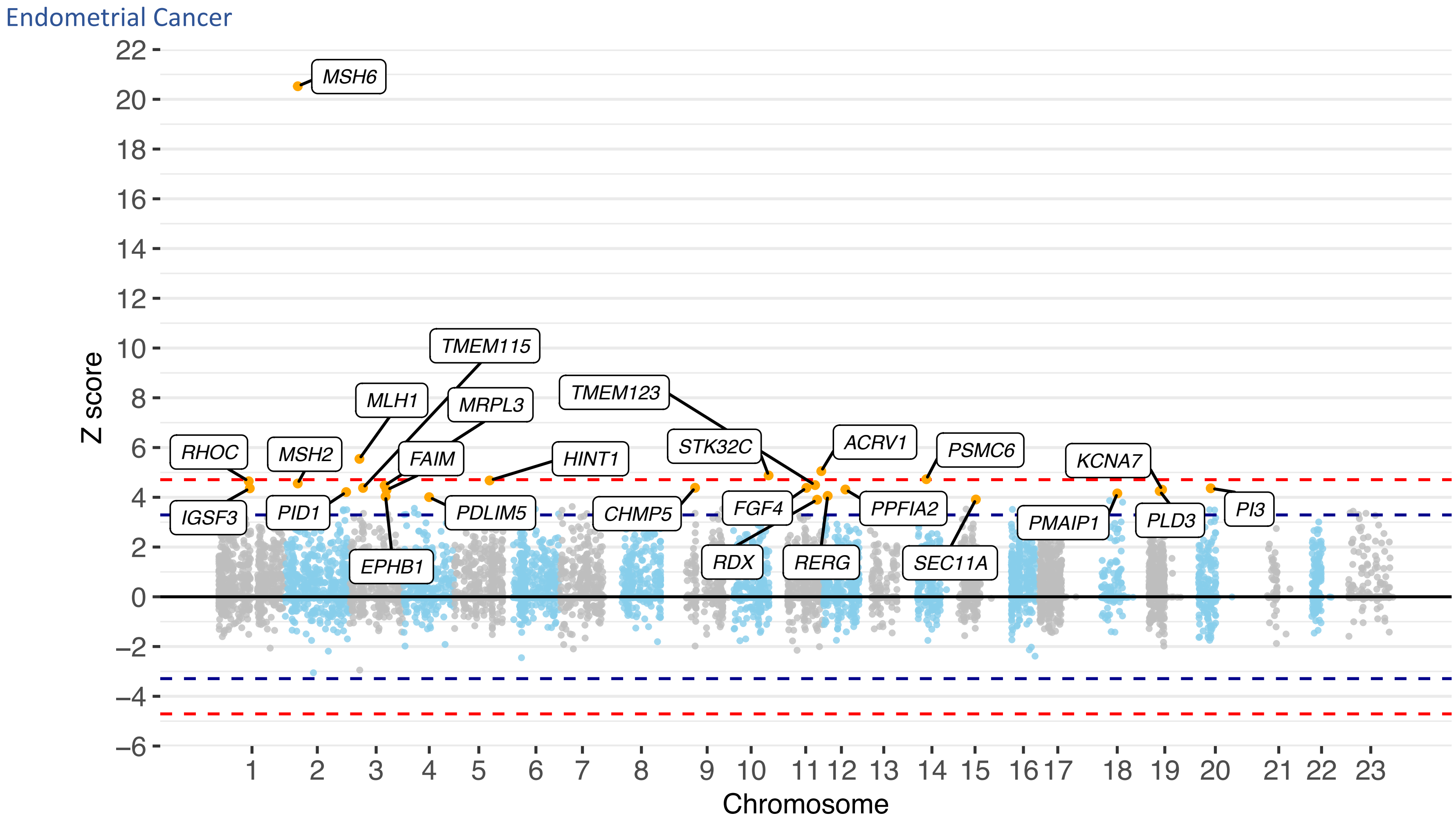

Figure S3 | Manhattan plot of z scores from assessing the association between protein-truncating variant carriers within genes and endometrial cancer risk, using model 1. The x axis is the chromosomal position, and the y axis is the z score from testing H0: β = ln(OR) = 0 (two-tailed) by LRT to the null model. The blue lines correspond to z = ±3.29, P = 0.001, the red lines correspond to z = ±4.71, P = 2.5 × 10−6. All labelled genes are those with P < 0.001. All P-values are unadjusted for multiple testing.

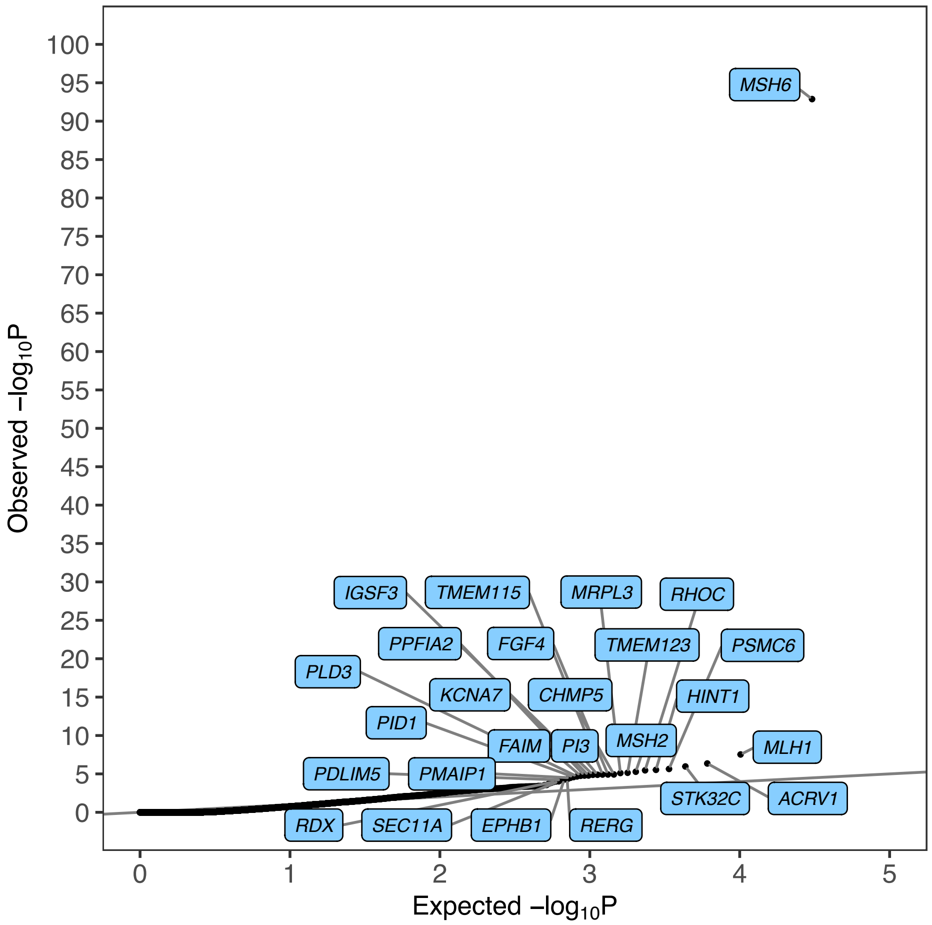

Figure S4 | Quantile–quantile plot of P values from assessing the association between protein-truncating variant carriers and endometrial cancer risk. P-values are from testing H0: $\beta = \ln(OR) = 0$ by LRT to the null model (two-tailed). The x-axis is the expected log10 P values from the null hypothesis, the y-axis is the observed log10 P value. Highlighted genes have $P < 0.0001$. Highlighted genes in blue are associated with an increased risk of endometrial cancer and highlighted genes in cream are associated with decreased risk of endometrial cancer. All P-values are unadjusted for multiple testing.

Ovarian Cancer

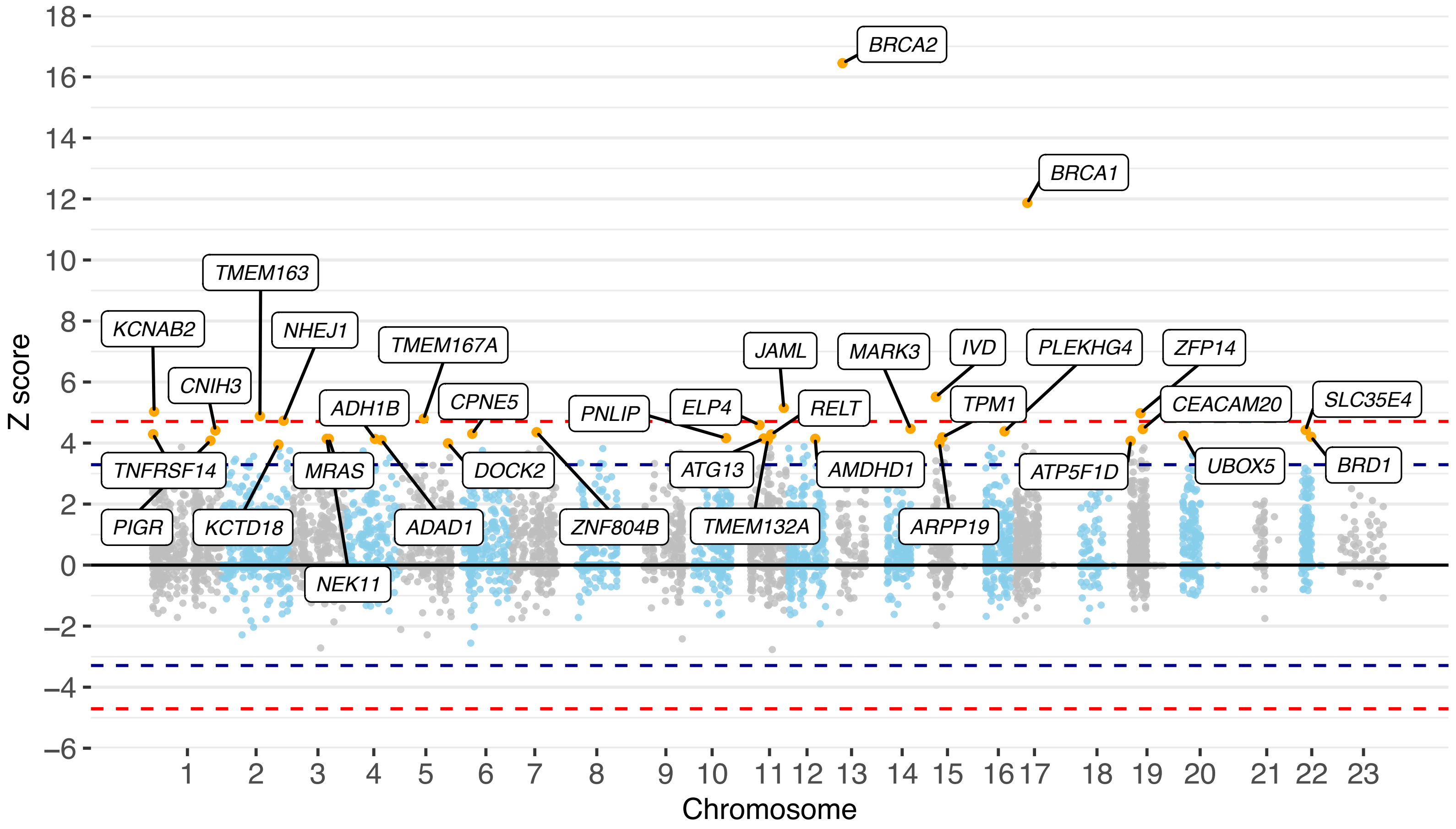

Figure S5 | Manhattan plot of z scores from assessing the association between protein-truncating variant carriers within genes and ovarian cancer risk, using model 1. The x axis is the chromosomal position, and the y axis is the z score from testing H0: β = ln(OR) = 0 (two-tailed) by LRT to the null model. The blue lines correspond to z = ±3.29, P = 0.001, the red lines correspond to z = ±4.71, P = 2.5 × 10−6. All labelled genes are those with P < 0.001. All P-values are unadjusted for multiple testing.

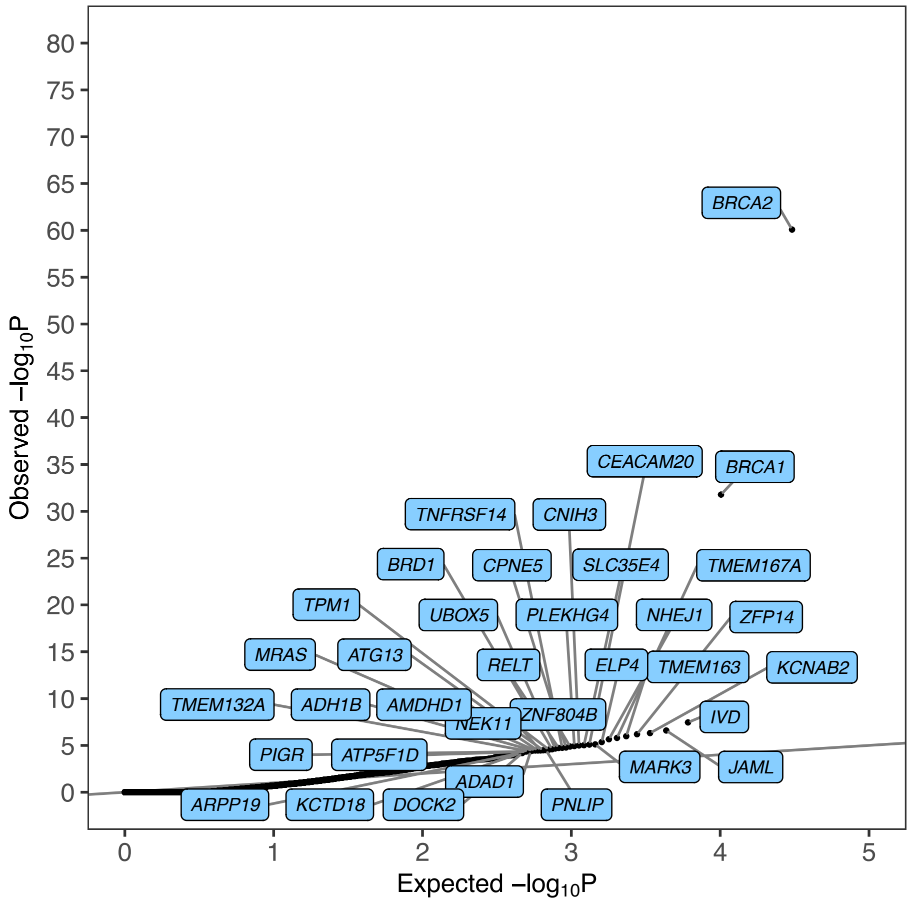

Figure S6 | Quantile–quantile plot of P values from assessing the association between protein-truncating variant carriers and ovarian cancer risk. P-values are from testing H0: $\beta = \ln(OR) = 0$ by LRT to the null model (two-tailed). The x-axis is the expected log10 P values from the null hypothesis, the y-axis is the observed log10 P value. Highlighted genes have $P < 0.0001$. Highlighted genes in blue are associated with an increased risk of ovarian cancer and highlighted genes in cream are associated with decreased risk of ovarian cancer. All P-values are unadjusted for multiple testing

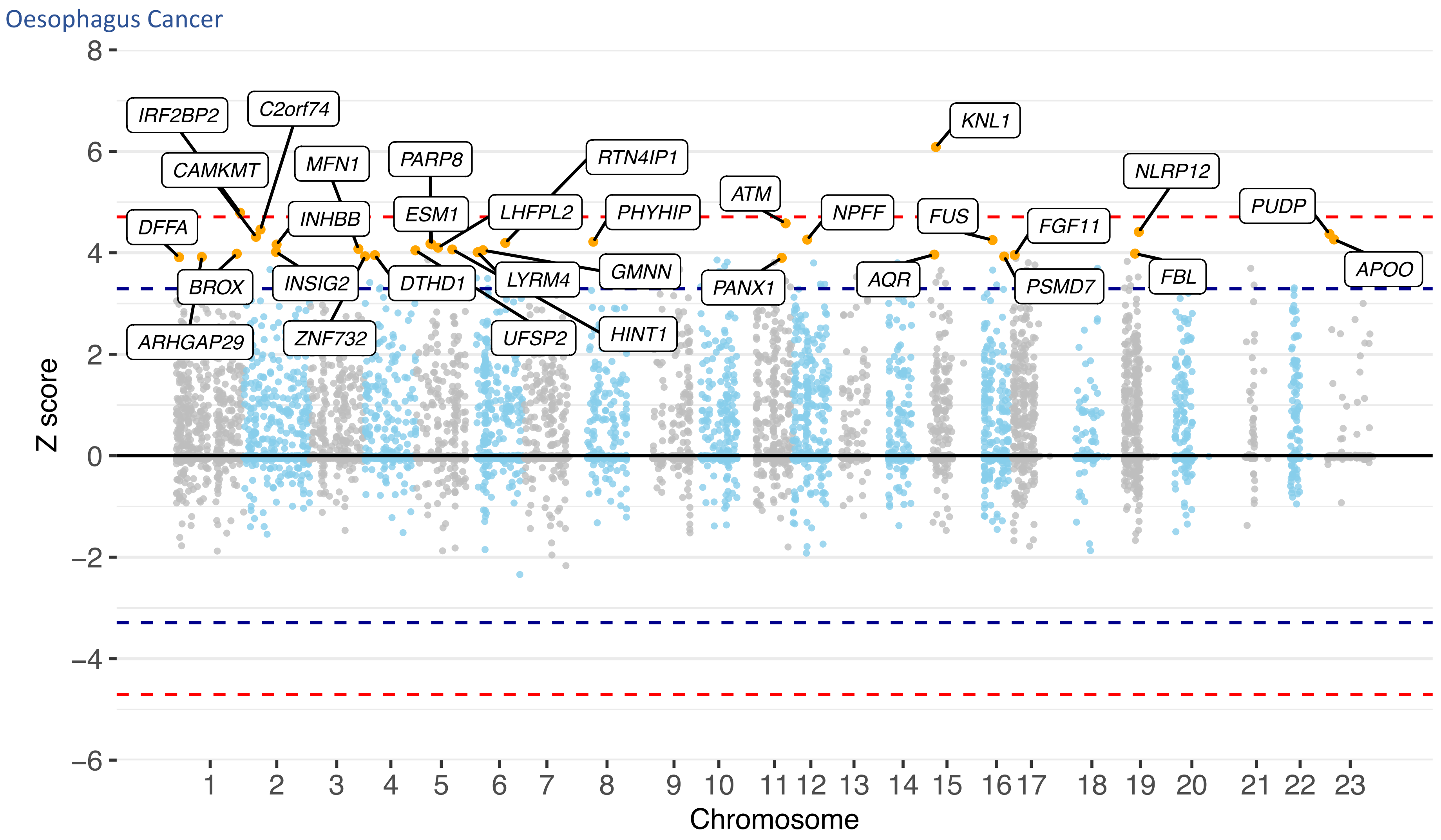

Figure S7 | Manhattan plot of z scores from assessing the association between protein-truncating variant carriers within genes and oesophagus cancer risk, using model 2. The x axis is the chromosomal position, and the y axis is the z score from testing H0: β = ln(OR) = 0 (two-tailed) by LRT to the null model. The blue lines correspond to z = ±3.29, P = 0.001, the red lines correspond to z = ±4.71, P = 2.5 × 10−6. All labelled genes are those with P < 0.001. All P-values are unadjusted for multiple testing.

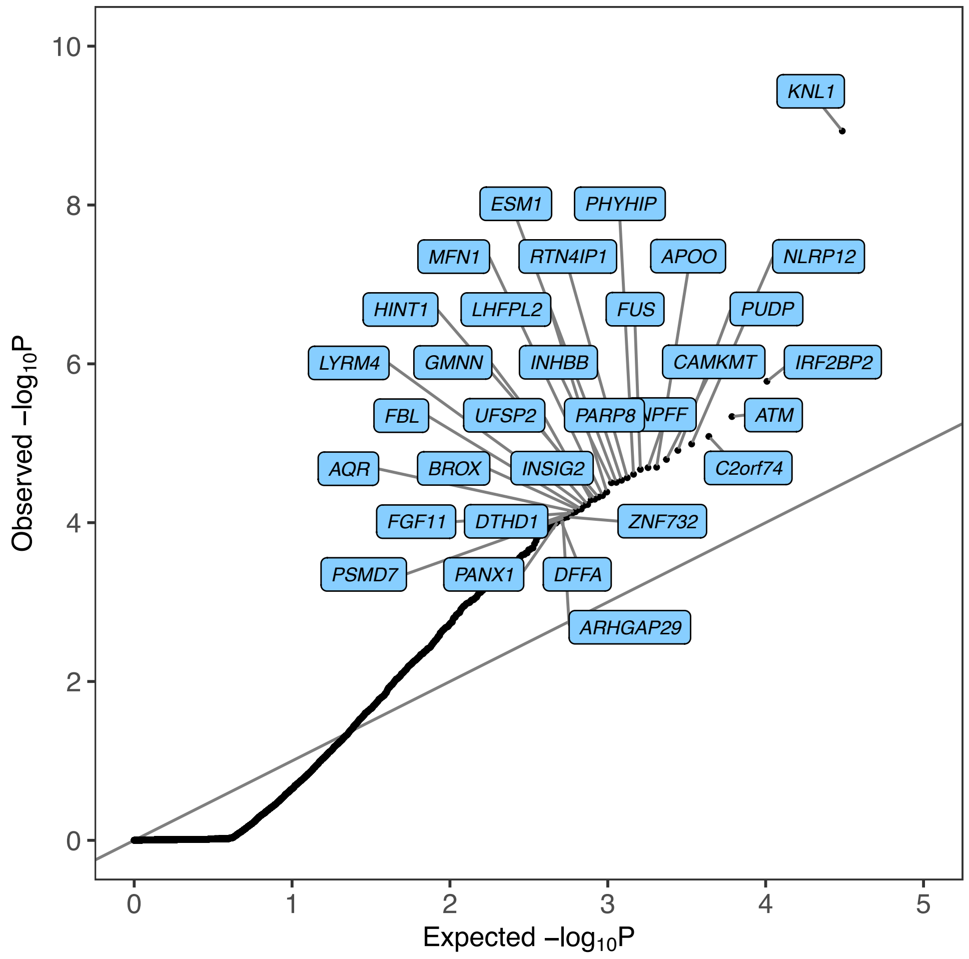

Figure S8 | Quantile–quantile plot of P values from assessing the association between protein-truncating variant carriers and oesophagus cancer risk. P-values are from testing H0: β = ln(OR) = 0 by LRT to the null model (two-tailed). The x-axis is the expected log10 P values from the null hypothesis, the y-axis is the observed log10 P value. Highlighted genes have P < 0.0001. Highlighted genes in blue are associated with an increased risk of oesophagus cancer and highlighted genes in cream are associated with decreased risk of oesophagus cancer. All P-values are unadjusted for multiple testing.

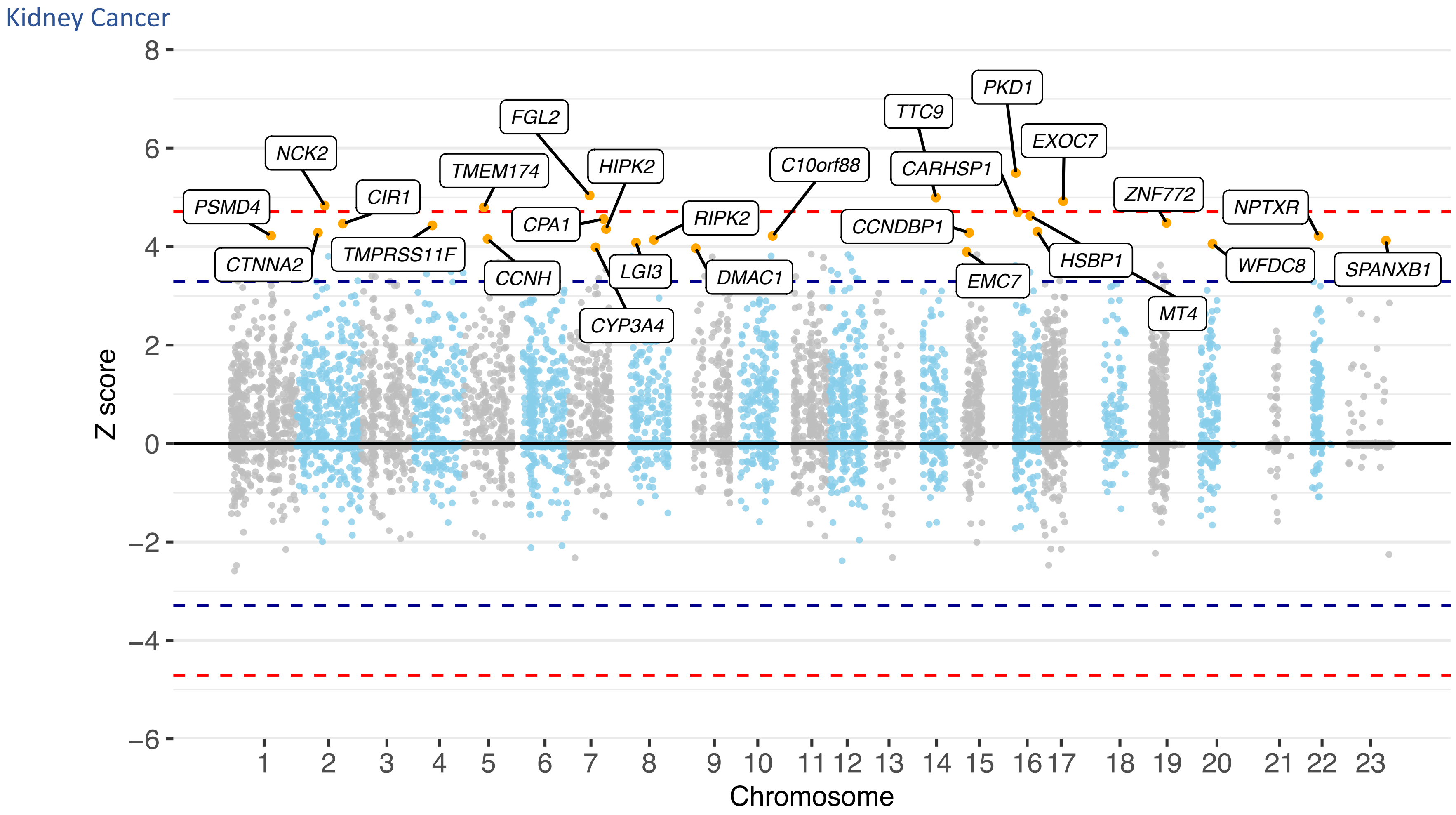

Figure S9 | Manhattan plot of z scores from assessing the association between protein-truncating variant carriers within genes and kidney cancer risk, using model 2. The x axis is the chromosomal position, and the y axis is the z score from testing H0: $\beta = \ln(OR) = 0$ (two-tailed) by LRT to the null model. The blue lines correspond to $z = \pm 3.29$, $P = 0.001$, the red lines correspond to $z = \pm 4.71$, $P = 2.5 \times 10{-}6$. All labelled genes are those with $P < 0.001$. All P-values are unadjusted for multiple testing.

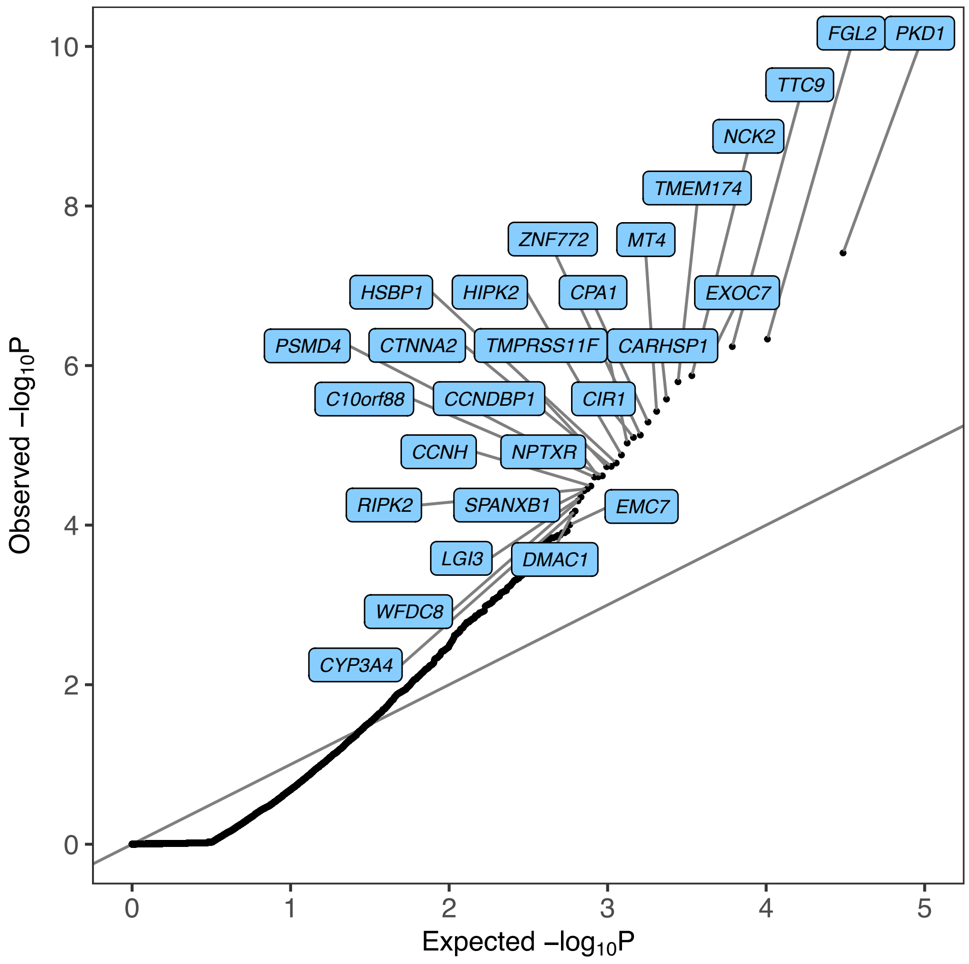

Figure S10 | Quantile–quantile plot of P values from assessing the association between protein-truncating variant carriers and kidney cancer risk. P-values are from testing H0: β = ln(OR) = 0 by LRT to the null model (two-tailed). The x-axis is the expected log10 P values from the null hypothesis, the y-axis is the observed log10 P value. Highlighted genes have P < 0.0001. Highlighted genes in blue are associated with an increased risk of kidney cancer and highlighted genes in cream are associated with decreased risk of kidney cancer. All P-values are unadjusted for multiple testing.

Bladder Cancer

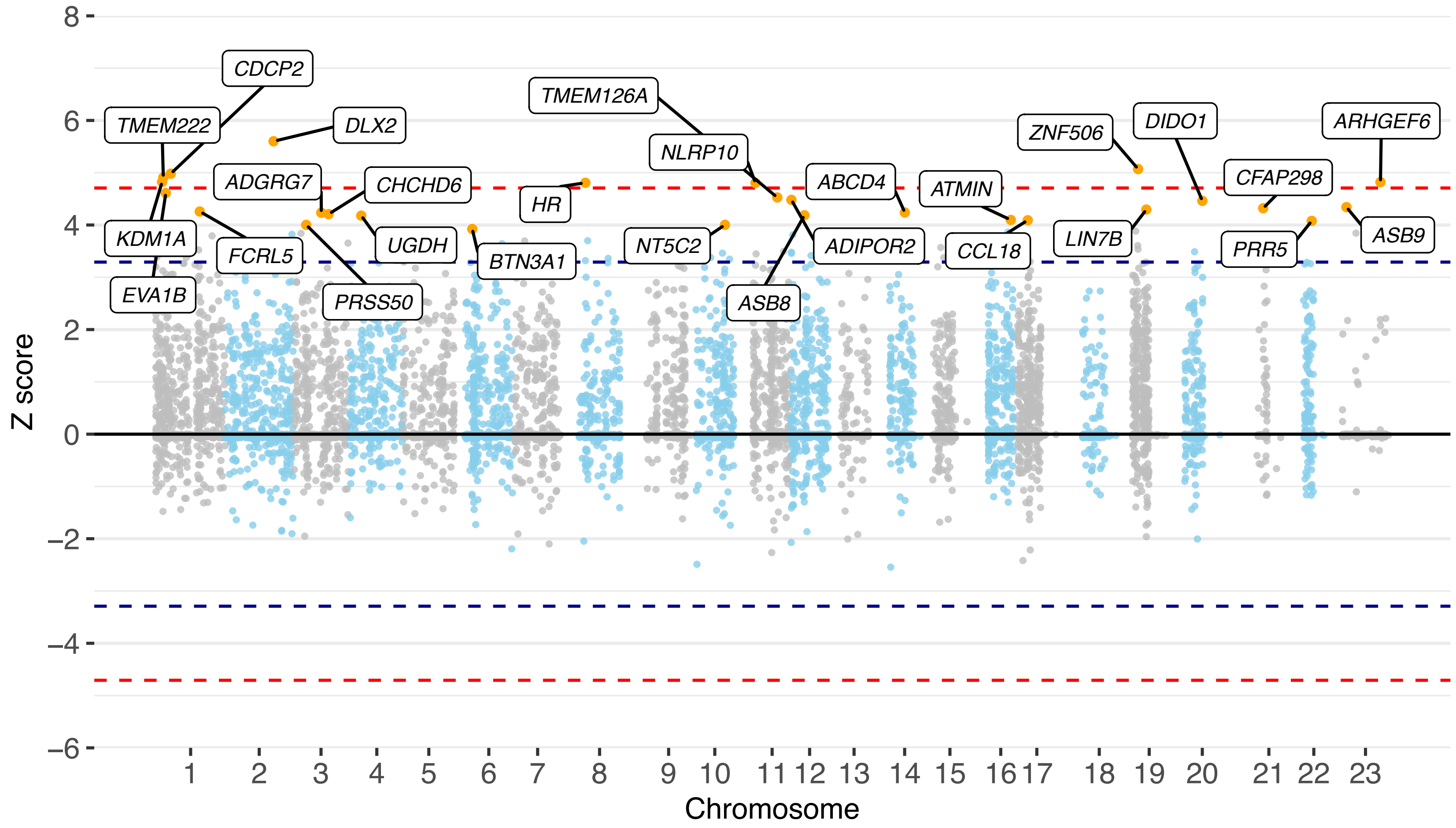

Figure S11 | Manhattan plot of z scores from assessing the association between protein-truncating variant carriers within genes and bladder cancer risk, using model 2. The x axis is the chromosomal position, and the y axis is the z score from testing H0: β = ln(OR) = 0 (two-tailed) by LRT to the null model. The blue lines correspond to z = ±3.29, P = 0.001, the red lines correspond to z = ±4.71, P = 2.5 × 10−6. All labelled genes are those with P < 0.001. All P-values are unadjusted for multiple testing.

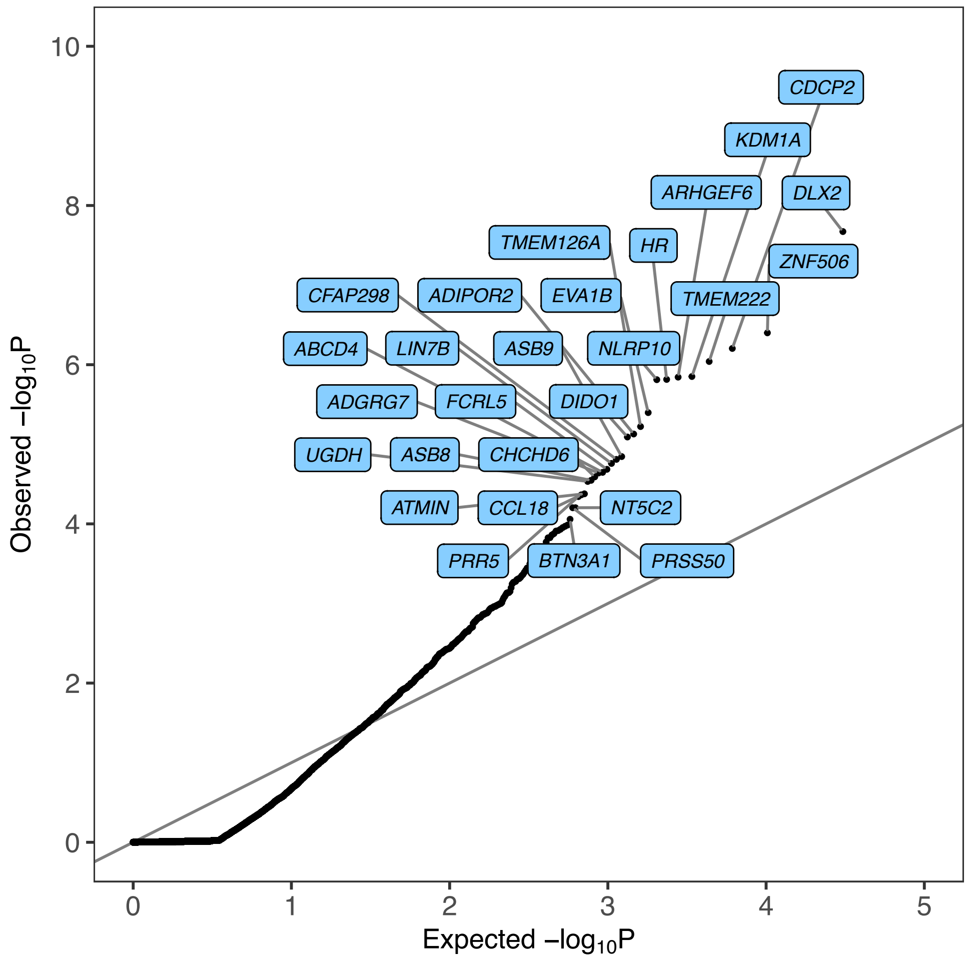

Figure S12 | Quantile–quantile plot of P values from assessing the association between protein-truncating variant carriers and kidney cancer risk. P-values are from testing H0: $\beta = \ln(OR) = 0$ by LRT to the null model (two-tailed). The x-axis is the expected log10 P values from the null hypothesis, the y-axis is the observed log10 P value. Highlighted genes have $P < 0.0001$. Highlighted genes in blue are associated with an increased risk of kidney cancer and highlighted genes in cream are associated with decreased risk of kidney cancer. All P-values are unadjusted for multiple testing.

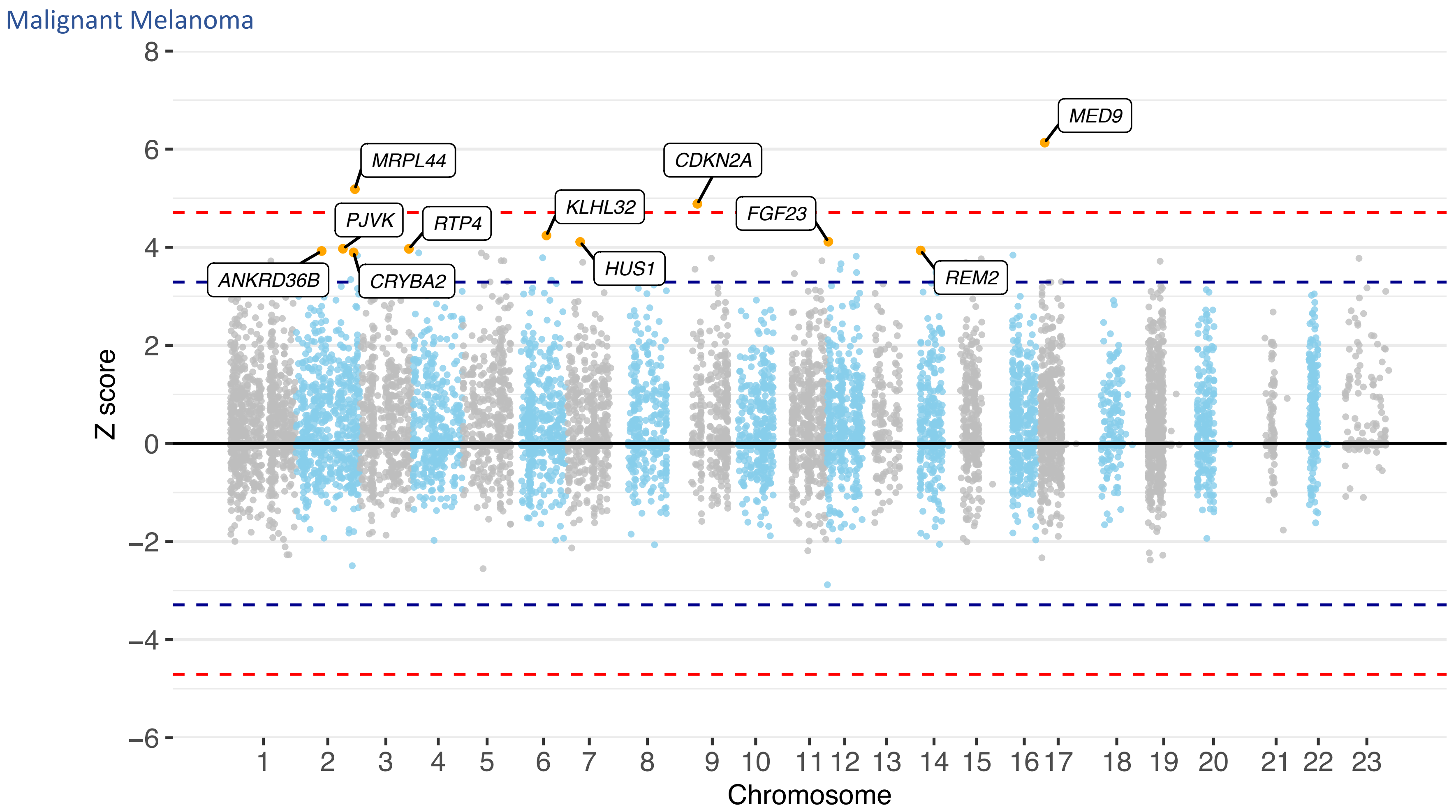

Figure S13 | Manhattan plot of z scores from assessing the association between protein-truncating variant carriers within genes and malignant melanoma risk, using model 2. The x axis is the chromosomal position, and the y axis is the z score from testing H0: β = ln(OR) = 0 (two-tailed) by LRT to the null model. The blue lines correspond to z = ±3.29, P = 0.001, the red lines correspond to z = ±4.71, P = 2.5 × 10−6. All labelled genes are those with P < 0.001. All P-values are unadjusted for multiple testing.

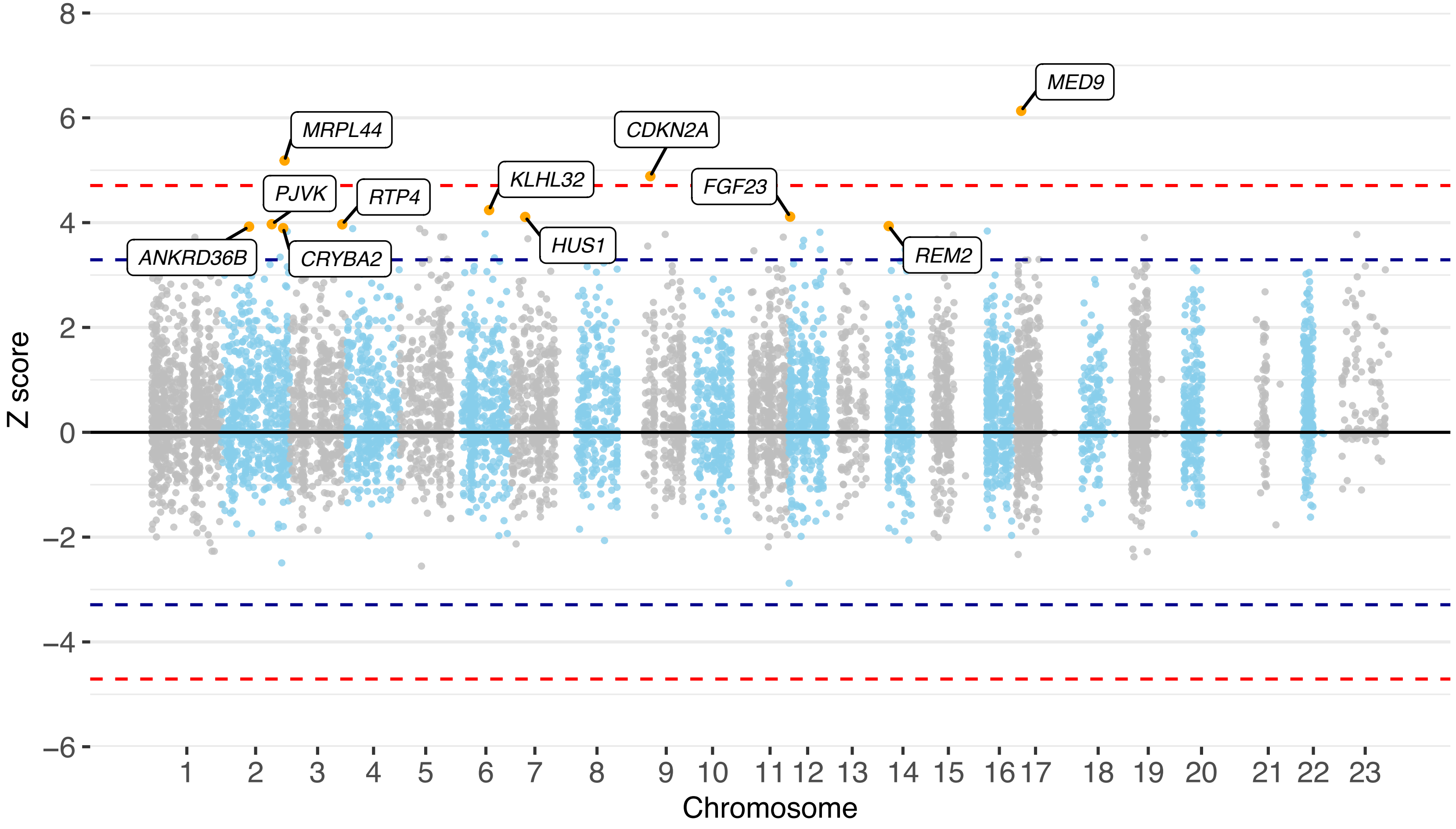

Figure S14 | Manhattan plot of z-scores from assessing the association between protein-truncating variant carriers within genes and malignant melanoma risk, using model 2. The x-axis is the chromosomal position, and the y-axis is the z-score from testing H0: $\beta = \ln(OR) = 0$ (two-tailed) by LRT to the null model. The blue lines correspond to $z = \pm3.29$, $P = 0.001$, the red lines correspond to $z = \pm4.71$, $P = 2.5 \times 10{-}6$. All labelled genes are those with $P < 0.001$. All P-values are unadjusted for multiple testing.

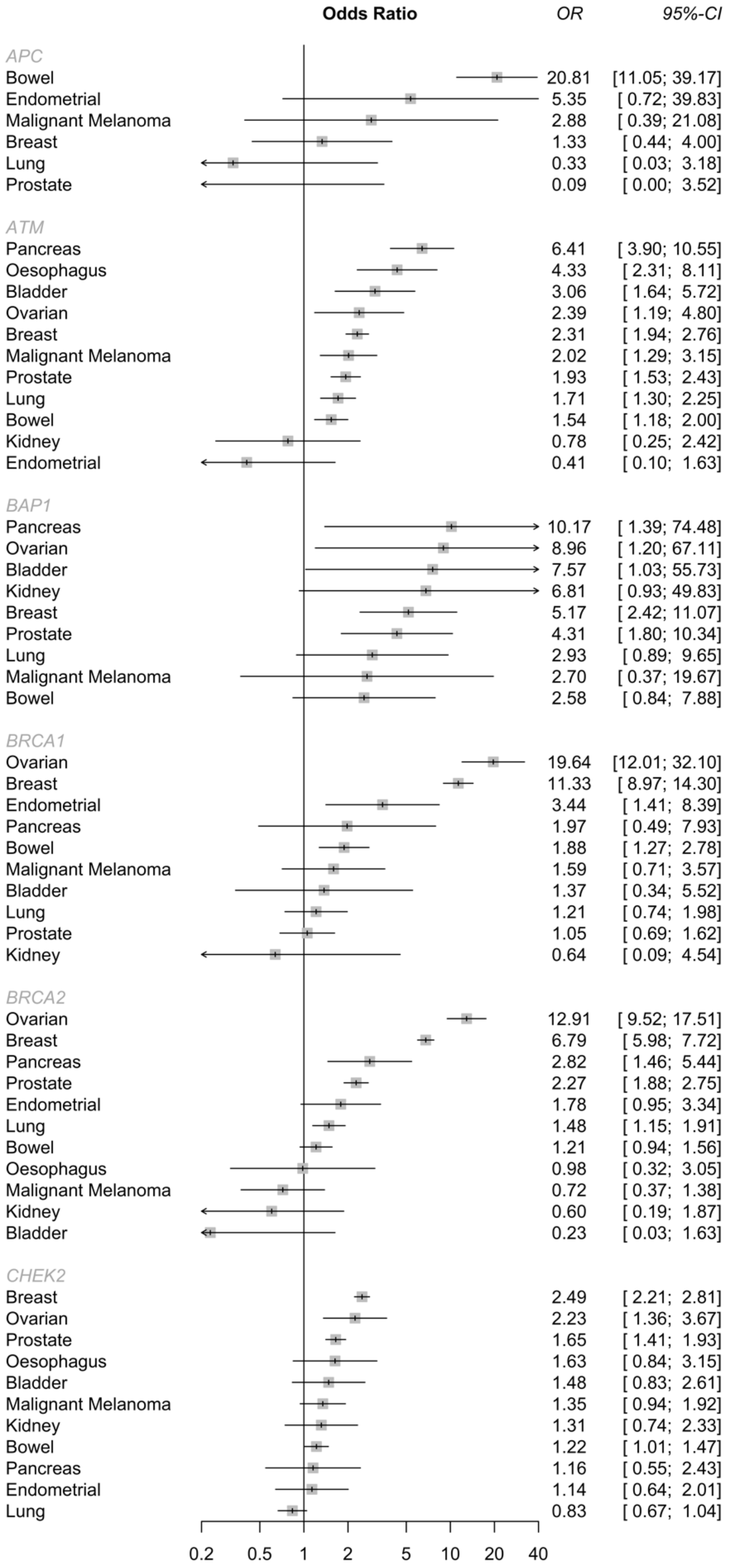

Figure S15a | Forest plot of PTV burden results across each cancer for the 11 genes with posterior probability > 0.8 in any analysis, and *NHEJ1*. For each gene, cancers with 0 case carriers were removed.

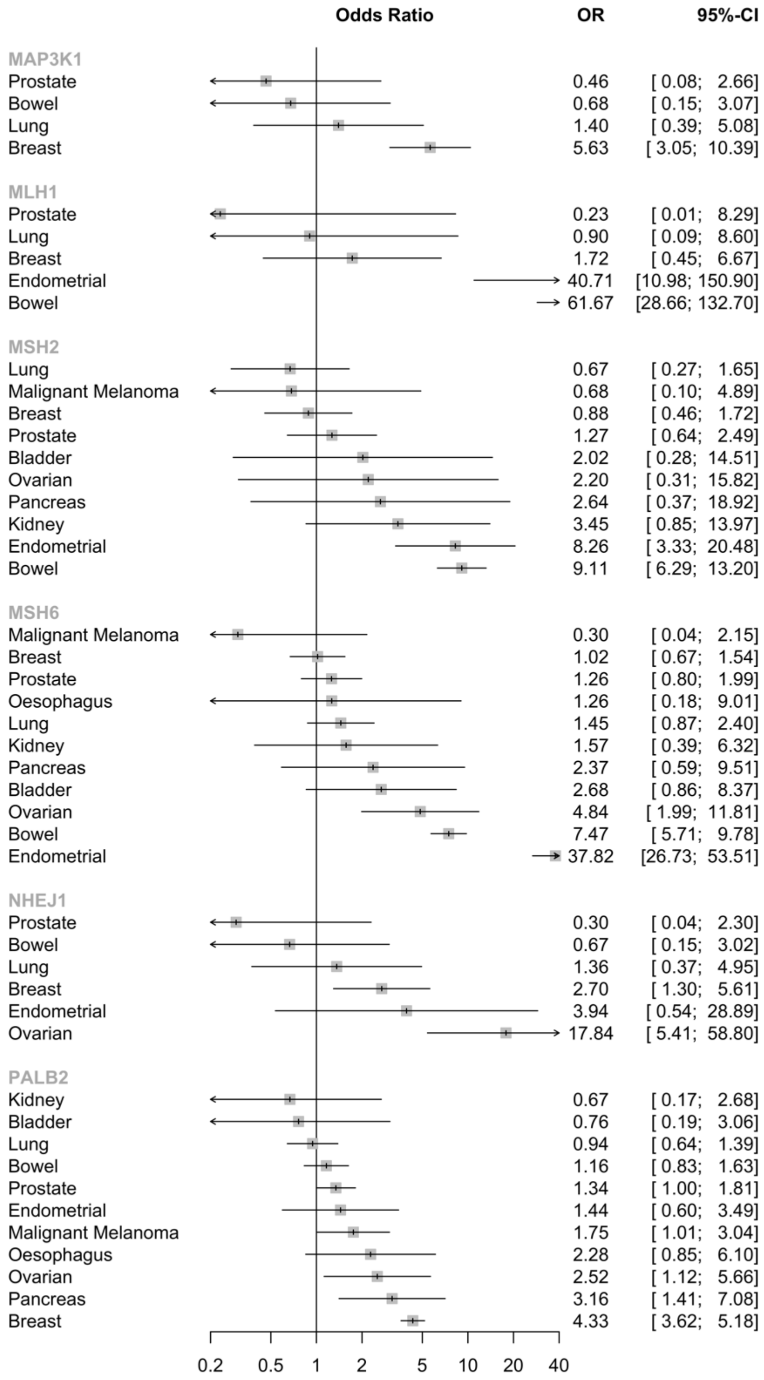

Figure S15b | Forest plot of PTV burden results across each cancer for the 11 genes with posterior probability > 0.8 in any analysis, and *NHEJ1*. For each gene, cancers with 0 case carriers were removed.
